# Modified electroconvulsive therapy for perinatal depression: scoping review

**DOI:** 10.3389/fpsyt.2025.1619098

**Published:** 2025-08-18

**Authors:** William V. Bobo, Owen Moore, Catherine B. Hurley, Robyn Rosasco, Emily E. Sharpe, Alyssa M. Larish, Katherine M. Moore, Hannah K. Betcher

**Affiliations:** ^1^ Department of Behavioral Sciences & Social Medicine, Florida State University College of Medicine, Tallahassee, FL, United States; ^2^ Center of Excellence for Perinatal Mood & Anxiety Disorders, Florida State University College of Medicine, Tallahassee, FL, United States; ^3^ Center for Medicine & Public Health Policy and Practice, Florida State University College of Medicine, Tallahassee, FL, United States; ^4^ Florida State University College of Medicine, Tallahassee, FL, United States; ^5^ Charlotte Edwards Maguire Medical Library, Florida State University College of Medicine, Tallahassee, FL, United States; ^6^ Department of Anesthesiology and Perioperative Medicine, Mayo Clinic, Rochester, MN, United States; ^7^ Department of Obstetrics & Gynecology, Mayo Clinic, Rochester, MN, United States; ^8^ Department of Psychiatry & Psychology, Mayo Clinic, Rochester, MN, United States

**Keywords:** perinatal depression, peripartum depression, postpartum depression, prenatal depression, electroconvulsive therapy, non-invasive brain stimulation

## Abstract

**Background:**

Modified electroconvulsive therapy (mECT), the administration of ECT under general anesthesia with muscular relaxation, is indicated for perinatal depression complicated by high severity, psychosis, catatonia, or resistance to conventional therapeutics; however, knowledge gaps remain regarding its effectiveness and safety in depressed patients and its fetal/neonatal risk profile.

**Materials and methods:**

We conducted a scoping review of the literature describing the effectiveness and safety (maternal, fetal, and neonatal) of mECT for perinatal depression. Online databases were searched (inception to December 31, 2024) to identify clinical trials, observational studies, case series, and case reports that were topically relevant. Information on key methodological details, clinical characteristics, interventions, and outcomes from each report was extracted by all investigators working in pairs, using an electronic abstraction form.

**Results:**

A total of 82 reports (with information on >1,300 pregnancies/deliveries) were included, consisting mainly of case reports (n=57) and case series (n=14), with the remaining citations being non-randomized or retrospective studies. The reviewed reports collectively described a broad spectrum of effectiveness and safety outcomes associated with predominantly acute mECT across multiple forms of perinatal depression, multiple trimesters of pregnancy, and the postpartum. mECT conferred rapid benefit for depressive, psychotic, and catatonic symptoms in severely depressed perinatal patients when effectiveness outcomes were described. The most frequent adverse events were generally mild and transient. However, cases of placental abruption (n=1), premature delivery (n=21), congenital malformations (n=6), and stillbirth (n=4) were also reported across the reviewed reports. Due to limited information, causal links between mECT and many adverse events were difficult to establish and inferences about differential effectiveness and safety between important patient subgroups or variations in mECT technique could not be drawn.

**Conclusion:**

mECT appears to be an effective acute phase treatment for severely ill perinatally depressed patients. Although the maternal safety profile of mECT appears reassuring, the available data are far from comprehensive. Moreover, fetal and neonatal safety risks are even less-well-characterized. mECT should be regarded as an important therapeutic option for severe cases of perinatal depression. Informed consent practices should reflect the knowledge gaps highlighted in this review in addition to the well-known side-effects of mECT and the substantial adverse consequences of untreated or undertreated maternal depression.

**Systematic Review Registration:**

This project was registered on Open Science Forum, 10.17605/OSF.IO/KB67J.

## 1 Introduction

Depression is among the most common complications in the perinatal period, spanning pregnancy through the first postpartum year. A 2005 systematic review estimated prevalence rates of 8.5%-11% for antenatal depression and 6.5%-12.9% for postpartum depression (PPD) in the U.S., including cases of unipolar major depression and minor depression (1). Other reviews have documented even higher average prevalence rates of 17% for antenatal depression and 13% for PPD (2). Beyond high prevalence, the public health importance of perinatal depression is highlighted by its association with increased maternal, neonatal, and early childhood morbidity (including negative effects on language, motor, and emotional development), poor obstetric outcomes, economic loss, and early maternal mortality including death by suicide (3–6). Indeed, perinatal depression presents across a broad severity spectrum, ranging from mild symptoms to behavioral emergencies requiring psychiatric hospitalization (7, 8).

In non-perinatal patients, modified electroconvulsive therapy (ECT), the administration of ECT under general anesthesia with muscular relaxation, is a high-priority treatment for refractory unipolar or bipolar depression, psychosis, refractory catatonias, and other psychiatric conditions for which the customary lag times to therapeutic benefit with conventional antidepressive treatments would be unacceptable, including cases with high suicide risk, evidence of medical or nutritional compromise, and others (9, 10). mECT is also indicated for perinatal depression complicated by high severity, psychosis, catatonia, or resistance to conventional approaches (11).

Previous reviews on the effectiveness and safety of mECT during the perinatal period have broadly supported the utility of mECT for severe perinatal depression but have arrived at mixed conclusions regarding the interpretation of obstetric and fetal/neonatal risks (12–20). And with relatively few exceptions (11), adaptations to standard mECT technique for perinatal patients were often not summarized. We thus conducted a scoping review to provide an updated survey of the published literature on mECT for perinatal unipolar or bipolar depression and to identify important but underdeveloped areas in need of further study.

## 2 Materials and methods

### 2.1 Search strategy

We conducted a scoping review of the literature regarding mECT for perinatal depression, guided by the Preferred Reporting Items for Systematic Reviews and Meta-Analyses Extension for Scoping Reviews (PRISMA-ScR) (21). A comprehensive literature search was conducted on December 31, 2024, by a research librarian (RR), in collaboration with the investigative team, using nine databases and registries (see **Supplementary Table S1** and **Appendix**). The reference sections of reviewed papers and selected systematic and meta-analytic reviews were also be used to locate potentially relevant papers. The search strategy facilitated the retrieval of both published and grey literature references.

### 2.2 Inclusion and exclusion criteria

We selected relevant reports of the effects of mECT for human perinatal depression, published in English, based on the following population/problem, intervention, comparators/controls, and outcome(s) (PICO) elements:

- Population/problem: Perinatal depression was defined as clinically significant depression occurring during pregnancy and/or within the 12 months following the date of delivery. We included papers with unipolar or bipolar depressed patients, any subtype or severity level, with or without psychotic features. Perinatal catatonia or psychosis cases were considered if a mood disorder diagnosis was specified as an underlying cause, presumed or established. Cases where an underlying mood disorder diagnosis was unspecified could still be included if there was enough detail to suggest the presence of an acute episode of depression, based on agreement between two independent reviewers. Although we did not specify an age range, the inclusion of reproductive-aged persons was presumed given our focus on perinatal depression.
- Intervention: We required the use of mECT as a treatment intervention (with or without co-interventions), using any stimulus parameters or electrode placement montages. Reports describing acute-, continuation-, or maintenance-phase mECT treatments were included.
- Comparators/controls: We included both controlled and non-controlled studies. For controlled research, we did not define acceptable or non-acceptable comparator groups or conditions, as adequate control group design for randomized trials of ECT for depression is debated (22, 23).
- Efficacy or Effectiveness Outcome(s): Efficacy/effectiveness outcomes included acute-phase reduction (improvement) in the severity of depressive symptoms, acute-phase categorical treatment outcome (e.g., remission/full response, partial response, non-response, etc.), duration of clinical response, and maintenance phase effectiveness (e.g., time to relapse, recurrence, or loss of remission or response).
- Safety/Tolerability Outcome(s): Safety/tolerability outcomes included acceptability of mECT as an acute or maintenance treatment (estimated using all-cause dropout rates), cognitive effects based on neuropsychological tests or bedside measures, non-cognitive adverse maternal effects and obstetric safety endpoints (e.g., acute hyper or hypotension, placental abruption, uterine contractions, preterm labor or premature rupture of membranes, difficulty with airway management, aspiration, etc.), and fetal, neonatal, and childhood developmental complications (e.g., intrauterine fetal demise, fetal growth restriction, changes in fetal heart rate, congenital malformations, respiratory depression, low Apgar scores at birth, developmental delay, etc.).

### 2.3 Study selection

After excluding duplicate articles, five investigators (WVB, OM, KMM, AML, HKB) worked in pairs to screen the titles and abstracts to exclude irrelevant papers. The remaining articles were then subjected to full-text review by six investigators (WVB, OM, KMM, EES, AML, HKB) who worked in pairs to exclude reports that failed to meet inclusion/exclusion criteria. Discrepancies at each step were resolved by discussion and consensus.

### 2.4 Data extraction and analysis

Data extraction was performed by all investigators, who worked independently in pairs, using a standard electronic extraction form. Disagreements were resolved via discussion and consensus. When necessary, an additional team member with specific domain expertise served a tie-breaking role. In accordance with PRISMA reporting guidelines for scoping reviews (PRISMA-ScR) (21), methodological quality and risk of bias assessments were not reported.

The following information was extracted from the individual studies (see **Supplementary Table S2**): (1) Study characteristics including publication year, authors, study design/report type, and treatment setting; (2) Subject/enrollee details including mood disorder diagnoses, mECT indication(s), definitions of treatment resistance (if applicable), maternal age, multiple gestation status, obstetric and general medical morbidities, pre-ECT medications, and use of assisted reproductive technology; (3) Treatment details including ECT electrode placement, pulse width, frequency of mECT administration, and ECT dose; (4) Anesthesia technique including anesthetic induction agent(s), pharmacological adjuncts to anesthesia, and airway management approach; (5) Adaptations to standard mECT technique including maternal and fetal surveillance methods; and (6) Effectiveness and safety measures and outcomes. Selected characteristics were summarized as proportions and were presented in table or graphical form.

## 3 Results

### 3.1 Format and design characteristics of the included reports

**Figure 1:**
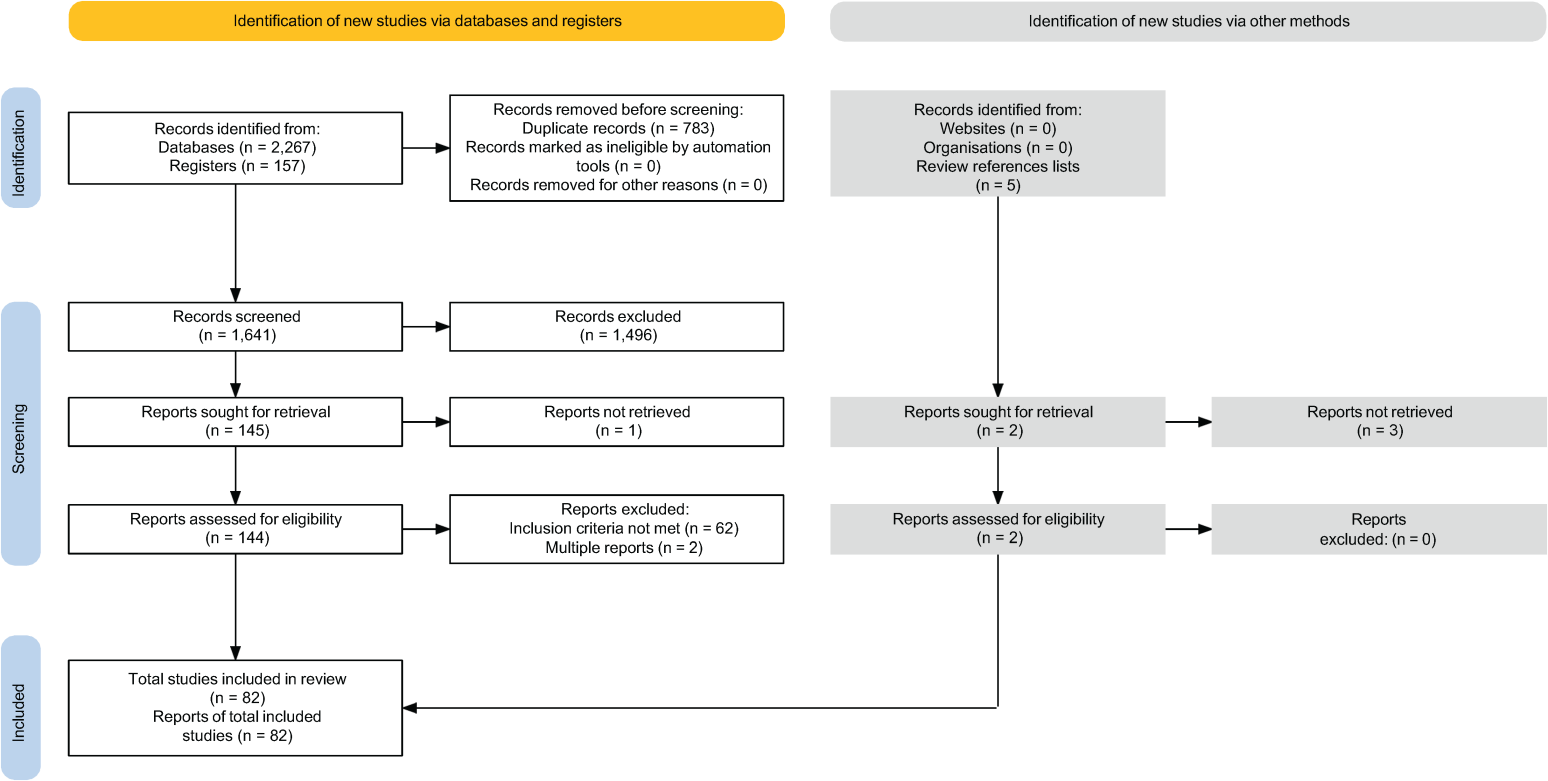
PRISMA flow diagram.

A total of 2,424 citations were identified from the initial literature searches across 14 registers and databases. After removing duplicates, 1,643 records underwent title/abstract screening, 146 of which were subjected to full-text review to determine eligibility for inclusion. On full text review, one case was found in both a published abstract and a published single case report, the former of which was excluded. We also included one of two full-length reports that described the same case. The remaining 82 reports (published between 1974 and 2024) met inclusion/exclusion criteria (**Figure 1**).

**Figure 2:**
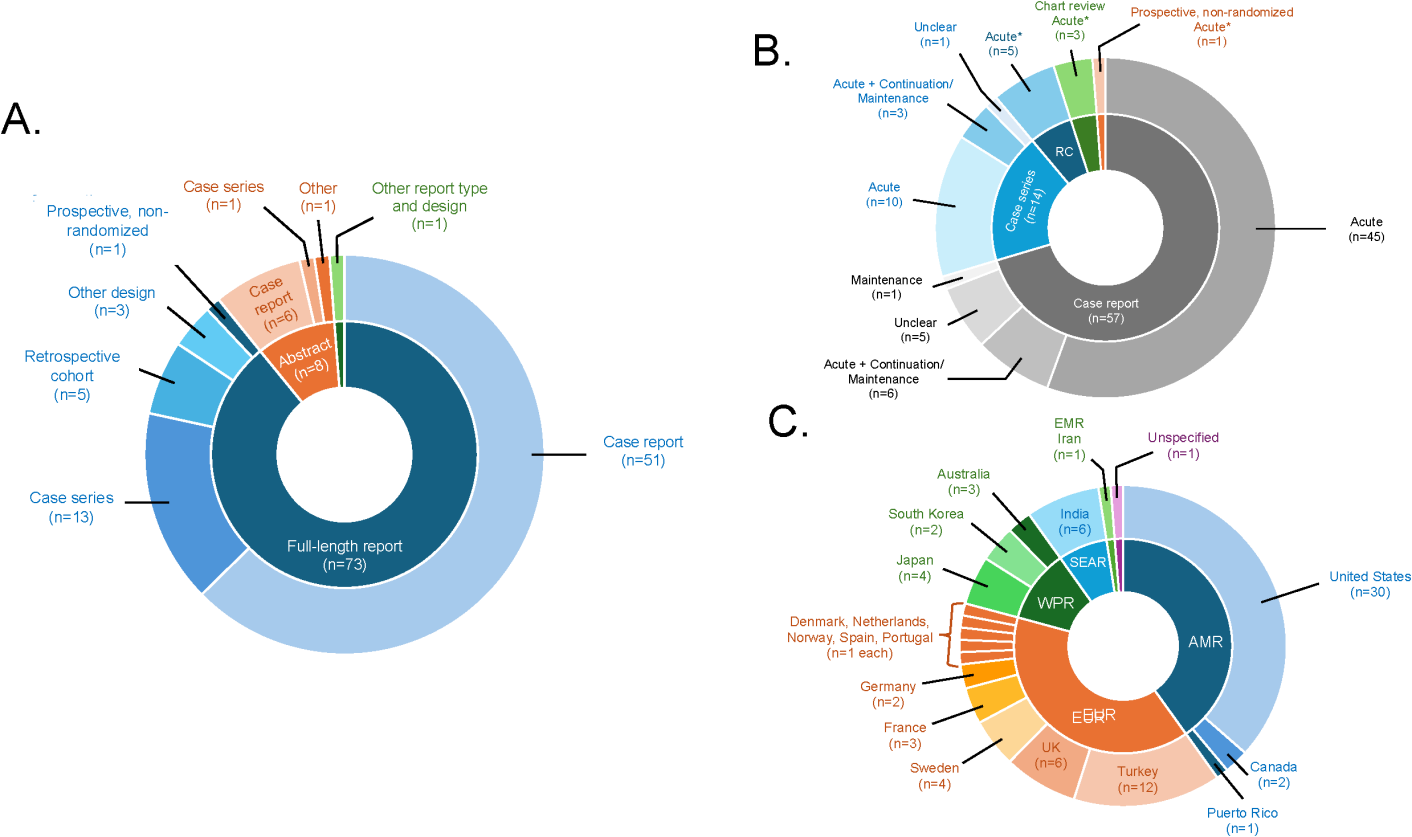
Graphical summary of reviewed studies. **(A)** displays the information on study or report design according to publication status (full-length report, conference abstract, other). **(B)** displays the information on phase(s) of modified ECT (mECT) treatment, by study or report design (including case reports, case series, chart review studies, prospective/non-randomized studies, and retrospective cohort studies [RC], absent one first-person account of postpartum mECT treatment and one qualitative mECT study). **(C)** displays the information on the countries in which individual studies or reports were conducted by World Health Organization region (including the Americas [AMR], the Eastern Mediterranean Region [EMR], the European Region [EUR], the South-East Asia Region [SEAR], and the Western Pacific Region [WPR]).

As shown in **Figure 2A**, 73 reports were published as full-length reports, while the remaining reports were published as abstracts (n=8) or other formats (n=1). Most reviewed citations were from case reports (n=57) and case series (n=14), with the remaining citations being from retrospective cohort, non-randomized prospective studies, or other designs. Sample sizes ranged from single case reports (n=1) to 793 individuals. Most of the included reports were from North America, followed by Europe, Western Pacific, and South-East Asian regions (**Figure 2B**).

### 3.2 Age and clinical characteristics of mECT-treated patients

Mean or median ages from case series and observational studies ranged from 23.0 to 37.0 years. The age range of individual cases was 16.5 to 48 years. As shown in **Tables 1** and **2**, several reports included mixed samples of patients with severe mood disorders, psychotic disorders, unspecified postpartum psychoses, or unspecified catatonia. The most common perinatal mood disorder diagnoses were unspecified nonpsychotic unipolar depression (36 reports), unspecified psychotic unipolar depression (17 reports), nonpsychotic unipolar or bipolar major depression (12 reports each), unspecified bipolar disorder (11 reports), and unspecified unipolar depression with catatonic symptoms or bipolar mixed episodes with severe depression or suicidality (6 reports each).

**Table 1:** Characteristics of observational and retrospective studies of modified electroconvulsive therapy (mECT) for perinatal depression.

Reference	Country/Data source	Study design (n)	Exposure group(s)	Main endpoint(s)	Confounding management	Main effective-ness results	Main safety/tolerability results
Arinson et al. (24)	Sweden/ Population-based registries	Retrospective cohort (n=793)^a^	Main exposure group, ECT for psych-iatric disorder during pregnancy (pregnant ECT group) Control group, other pregnancies from the pregnant ECT group that did not involve ECT treatment (non-ECT additional pregnancy group) Control group, severe prenatal psychiatric disorders requiring hospitalization who did not receive ECT (non-ECT pregnant inpatient group)	Response (CGI-I scores of 1 or 2^b^) within 7 days of finishing index ECT series) Adverse events (premature births, 5-minute Apgar score <7, Cesarean delivery, post-delivery SGA or LGA delivery, congenital malformations, etc.)	Propensity score matching on age, parity, concurrent psychotropic medications, comorbid anxiety disorder, preeclampsia, diabetes	Response rates were similar for the pregnant ECT group and 216 non-pregnant women who received ECT (74% vs 65%, OR 1.61 [95% CI 0.79, 3.27]).	Rates of most reported AE’s were similar between the main exposure group and the two control groups.^c^ Premature delivery: Significantly higher risk in main exposure group than non-ECT pregnant inpatient group (14.4% vs 9.0%, OR 2.33 [1.15, 4.73]) but not the non-ECT additional pregnancy group (10.9%; OR 2.16 [0.75, 6.22]). Low 5-minute Apgar score: Significantly higher risk in main exposure group than non-ECT pregnant inpatient group (9.3% vs 2.1%, OR 3.68 [1.58, 8.55]) but not the non-ECT additional pregnancy group (2.2%, OR 2.53 [0.59, 10.90]). Stillbirths reported in two (2.1%) pregnancies in the main exposure group, and in one pregnancy each in the control groups.
Babu et al. (25)	India/Consecutively hospitalized subjects	Prospective non-randomized (n=78)^d^	ECT for PPP No ECT for PPP	CPRS and BFCRS scores Hospital LOS Selected maternal AE’s from ECT	All comparisons were unadjusted	CPRS and BFCRS scores were not specified. CPRS scores were described as significantly decreased from baseline at discharge in each group, with no significant between-group difference. Duration of admission was lower in ECT group (19 vs 23 days), with no significant between-group difference	Most common AE’s with ECT were antero- grade amnesia (n=6 [17.6%]) and prolonged seizures (n=4 [11.7%]). Fifteen women who receive ECT were admitted with their infants, 10 of whom con-tinued to breast-feed their infants without AE’s.
Hauge et al. (26)	Denmark/Population-based registries	Retrospective cohort (n=91)^e^	ECT for incident postpartum mood or psychotic disorders^e^	Description of acute and post-acute treatment Risk of hospital readmission after 6 months	Not applicable	Acute phase response to ECT was not described. 28 (30.8%) of 91 cohort members were readmitted (median time to readmission was 16 days [IQR 7, 51]). ECT-specific results were not reported.	Safety outcomes were not reported.
Haxton et al. (27)	UK/Scottish ECT Accreditation Network database	Retrospective cohort (n=35)^f^	Postnatal ECT while hospital-ized on mother-baby unit ECT in hospital-ized non-perinatal women	Reduction (improvement) in MADRS scores between baseline and after ECT	Matching (1:2) on age (within 5 years) and initial MADRS score (within 5 points)	Trend towards greater reduction in mean MADRS scores with ECT in the postnatal group than in the non-perinatal group (Δ = -10.1, p=0.06).	Safety outcomes were not reported.
Raghuraman et al. (28)	India/Hospital records	Retrospective chart review (n = 31)^g^	BT ECT for “severe mental illness” during the perinatal period BF ECT for similar indications	Reduction (improvement) in EPDS and CGI-S scores	All analyses unadjusted	Significantly lower post-treatment EPDS score with BF than BT (trend level difference for CGI-S score)	Headache reported with BT ECT (1 patient), “cognitive difficulties” reported with BF ECT (1 patient).
Reed et al. (29)	UK/Hospital-based ECT register	Retrospective chart review (n=114)^h^	ECT received for “puerperal psychosis” while admitted to mother-baby unit Inpatients <45 years of age who received ECT for “non-puerperal psychosis”	Ratings of clinical recovery ranging from 0 (no improve-ment or worsen-ing) to 3 (asymp-tomatic)	Multiple regression models (with stepwise covariate selection)	In analyses restricted to patients with depression, there were significantly higher (better) clinical recovery scores in the puerperal ECT group, both at the end of ECT and 1 month after ECT.	Safety outcomes were not reported.
Ronnqvist et al. (30)	Sweden/Population-based registers	Retrospective cohort (n=360)^i^	ECT received for PPD and/or PPP ECT received for non-postpartum indication	Relapse (defined as rehospitalization for psychiatric reasons or suicide) after 6 months, 1 year, and 2 years	Multivariable Cox regression models	Relapse rates were lower in the postpartum group at 6 months (28% vs 39%), 1 year (31% vs 50%), and 2 years (40% vs 55%). The risk of relapse was significantly lower in the postpartum group in univariable analyses (p=0.001) and was nearly so in multivariable analyses (p=0.051).^i^	Safety outcomes were not reported.
Rundgren et al. (31)	Sweden/Population-based registers	Retrospective cohort (n=370)^j^	ECT received for PPD and/or PPP ECT received for non-postpartum indication	Response (CGI-I score of 1 or 2)^b^ within 1 week after ECT treat-ment Remission (post-treatment CGI-S score of 1 or 2)	Matching of comparator group on age, diagnosis, prior antidepressant medication, and CGI-S score before ECT; statistical adjustment of logistic regression models	Significantly higher response rate (87.0% vs 73.5%) and remission rate (45.4% vs 29.9%) in the postpartum group.	Safety outcomes were not reported.
Saluja et al. (32)	Australia/Hospital records	Retrospective chart review (n=74)^k^	Descriptive study of treatments re-ceived on a mother-baby unit	Medications used and ECT treatments provided at 3 time points (on admission, half-way through admission, and at discharge)	Not applicable	Not applicable. Eight of 57 women with depression received ECT during their hospitalizations. All were trialed on ADs during admission prior to ECT.	Safety outcomes were not reported for any inter-vention, including ECT.

Key: AD, antidepressant medication; BFCRS, Bush-Francis Catatonia Rating Scale; BF, bifrontal ECT; BT, bitemporal ECT; CGI-I, Clinical Global Impression improvement subscale; CGI-S, Clinical Global Impression Scale severity subscale; CPRS, Comprehensive Psychopathological Rating Scale; ECT, electroconvulsive therapy; EPDS, Edinburgh Postnatal Depression Scale; IQR, inter-quartile range; LGA, large for gestational age; LOS, length of stay; MADRS, Montgomery-Asberg Depression Rating Scale; mECT, modified electroconvulsive therapy; PPD, postpartum depression; PPP, postpartum psychosis; SGA, small for gestational age; UK, United Kingdom.

^a^A total of 793 cases from population-based registers were described. There were 97 pregnancies in which ECT was administered, 54 of which had non-missing CGI-I values. The 97 ECT pregnancies were propensity score (PS)-matched to 388 pregnancies occurring in women who were psychiatrically hospitalized but received no ECT. The 54 ECT-treated pregnancy cases (with complete CGI-I data) were PS-matched to 216 non-pregnant cases (women) who also received ECT. ^b^Response was defined based on CGI-I score of 1 (very much improved) or 2 (much improved) within 7 days after finishing the index series of ECT treatments. ^c^None of the reported preterm births or other severe pregnancy outcomes occurred in close time proximity to ECT, weakening causal links between ECT and these outcomes. ^d^The study sample consisted of 78 consecutively hospitalized women with PPP, 34 of whom received ECT. Twenty-four women were diagnosed with depression, 32 were diagnosed with mania, and 22 were diagnosed with a “non-affective psychosis.” ^e^The cohort consisted of 91 women with evidence of new-onset (newly diagnosed) postpartum mood or psychotic disorder identified in population-based registers (39 with depression, 17 with a bipolar spectrum disorder/manic episode, and 35 with a psychotic disorder). Seventeen (18.7%) of 91 cohort members received ECT. ^f^A total of 12 patients received ECT for postnatal unipolar depression (8 patients), bipolar depression (2 patients), bipolar mixed episode (1 patient), or no specified diagnosis (1 patient). Controls consisted of 24 women matched 2:1 with postnatal ECT subjects on age and pre-ECT MADRS scores. ^g^Results were presented as a conference abstract. Thirty-one patients received ECT (BT in 18 patients, BF in 13 patients), 4 during the prenatal period and 27 during the postpartum period. The mixed sample included 3 patients with unspecified depression, 14 with bipolar disorder, 4 with postpartum psychosis, 6 with “acute transient psychosis,” and 4 with “psychosis NOS.” ^h^The cohort consisted of 58 women in the puerperal ECT group (42 diagnosed with a “depressive illness”) and 56 women in the non-puerperal ECT group (33 diagnosed with a “depressive illness”). ^I^The cohort consisted of 180 women with PPD/PPP who received ECT within 6 months following delivery and an equal number of controls who received ECT but for a non-postpartum psychiatric condition. ^j^The cohort consisted of 185 women with PPD/PPP who received ECT within 6 months following delivery and an equal number of matched controls who received ECT for non-postpartum depression/psychosis. ^k^The cohort consisted of 74 patients admitted to a mother-baby unit, 57 with depression, 8 of whom received ECT.

**Table 2:** Characteristics of case reports and case series describing modified electroconvulsive therapy (mECT) for perinatal depression.

Reference	Country	Diagnos(es), n cases	Trimester(s) of pregnancy or weeks postpartum	ECT delivery characteristics	Main effective-ness outcomes	Obstetric safety outcomes	Fetal/neonatal or nursing infant safety outcomes
Bak et al. (33)	Turkey	Mixed sample, 4 cases total Prenatal depression, n=1 Prenatal BP, n=2 Prenatal “atypical psychosis”, n=1	Mean gestational age 23 weeks, individual level data unspecified	ECT electrode placement unspecified; mean of 10 ECT applications (individual level data unspecified)	Not reported.	No maternal complications.	No complications in newborns. Normal development through first 1 month of life.
Balki et al. (34)	Canada	Prenatal BP-D, suicidal ideation, n=1	Second trimester	Acute RUL ECT, three stimuli given during session 1 owing to inadequate seizure activity	Not reported.	SE after third stimulus requiring high-dose BZD, thiopental, propofol, and DPHD, ICU transfer, and prolonged MV	Fetal death followed by spontaneous vaginal delivery
Ballone et al. (35)^a^	Unspecified	PPD, n=1	8 weeks postpartum	Acute RUL/UB ECT, 15 treatments, delivered in an ambulatory setting to permit breastfeeding	Conference abstract, outcomes unspecified	Not applicable.	Conference abstract, outcomes unspecified
Bergink et al. (36)^a^	Netherlands	Postpartum BP, n=7	Postpartum week(s) unspecified	Acute ECT, lead placement and frequency of treatments unspecified	Remission in one patient who received ECT owing to poor response to pharmacotherapy	Not applicable	Conference abstract, outcomes unspecified
Bulut et al. (37)	Turkey	Prenatal MDD, n=3 Prenatal MDD, psychotic features, n=3 Prenatal BP, n=5 Other prenatal depression, n=1	First trimester, n=6 Second trimester, n=3 Third trimester, n=3	Acute BT ECT treatments (all 12 cases), range 3–20 treatments Maintenance BT ECT (2 cases, one with MDD), 3 treatments each	Mean CGI score for all 12 cases reduced from 6.0 (baseline) to 2.6 (end of ECT sessions). Diagnosis-specific results were not provided.	One pregnancy was terminated early. Remaining pregnancies were un-complicated. Diagnosis-specific results were not provided.	One neonate with pes ekinovarus deformity. The remaining 10 new-borns were described as healthy after delivery.
Bhatia et al. (38)	USA	Prenatal MDD, n=1 (Case 1) Other prenatal depression, n=1 (Case 2)	Third trimester, n=2	Case 1 – Acute BT ECT, 3x/week, 6 treatments total Case 2 – Acute BT ECT, 5 treatments total	Case 1 – improvement after 6 treatments, remission at 6-month contact. Case 2 – improvement after 5 treatments, remission at 6-month contact.	Case 1 – transient mild uterine contractions Case 2 – transient uterine contractions, preterm labor on post-ECT day 7 (31 weeks EGA)	Case 1 – delivery of healthy infant at 39 weeks EGA. Case 2 – premature delivery of otherwise healthy infant at 35 weeks EGA, unspecified reason for delivery.
Bozkurt et al. (39)	Turkey	Prenatal psychotic depression, n=1	Second trimester, n=1	Acute BT ECT, 3x/week, 10 treatments Maintenance BT ECT, once monthly, 3 treatments (until 31 weeks EGA)	Remission, as evidence by reduced (improved) HAM-D score from baseline (33) through the 10^th^ treatment (7).	Pelvic pain after the 8^th^ and 9^th^ treatment.	Delivery of healthy infant at 38 weeks EGA.
Brown et al. (40)	USA	Prenatal psychotic depression, n=1	Second trimester, n=1	ECT electrode placement unspecified, 8 treatments	Not reported.	Patient required the use of a supraglottic airway owing to difficult ETI, and completed 8 ECT treatments without apparent adverse events.	Not reported.
Bulbul et al. (41)^b^	Turkey	Mixed sample, 33 cases total Prenatal MDD, n=19 Prenatal BD^c^, n=12 Schizophrenia, n=2	Multiple trimesters of pregnancy^b^	Acute ECT, electrode placement and treatment frequency unspecified	16 (84.2%) of 19 patients with MDD had a decrease (improvement) in CGI-S scores to ≤ 2 (borderline ill or not ill) 11 (91.7%) of 12 patients with BP^c^ had a decrease in CGI-S scores to ≤ 2	Diagnosis-specific information was not available. Three patients had transient uterine contractions during ECT requiring no specific intervention.	Diagnosis-specific information was not available. There was one stillbirth (cause not determined) and once case each of congenital hip dysplasia and SVT after myocarditis.
Bulbul et al. (42)^d^	Turkey	Mixed sample, 68 cases total Prenatal unipolar depression, n=43 Prenatal BP, n=20 (5 with BP-D, 5 with BP-MX, and 10 with BP-M)	First trimester, n=17 (unipolar depression), n=5 (BP) Second trimester, n=22 (unipolar depression), n=9 (BP) Third trimester, n=4 (unipolar depression), n=6 (BP)	ECT electrode placement and treatment frequency unspecified	Remission (HAM-D <7 or CGI-S ≤ 2) in 93% of patients with unipolar depression. Phase-specific information on treatment response was unavailable for patients with BP.	Outcomes unspecified apart from no cases of preterm delivery among 26 women with unipolar depression for whom birth information was available.	No medical problems reported in 30 infants born to mothers with unipolar depression for whom birth information was available. Of 17 infants born to mothers with BP, 16 were normal and 1 had a cardiac disease that healed with treatment.
Chase et al. (43)	USA	Perinatal MDD, psychotic features, n=1	Postpartum week(s) unspecified	Acute BT ECT, 16 treatments	Partial response after 5 treatments, remission after 16 treatments	Not reported.	Not reported.
Choi et al. (44)	S. Korea	Perinatal depression, n=1	Approximately 12 weeks postpartum	Acute BT ECT, stopped after one treatment	Not reported.	ECT stopped owing to treatment-emergent T6 vertebral fracture.^e^	Not reported.
(45)	UK	PPD, n=1^f^	Postpartum week(s) unspecified	Acute ECT, electrode placement unspecified, 6 treatments	Full response after 6 treatments	Not applicable.	Not reported.
DeAsis et al. (46)	USA	Prenatal BP-D, passive suicidal ideation, n=1	Second trimester, n=1	Acute RUL ECT, 10 treatments Continuation RUL ECT, 4 treatments	Remission	Prolonged seizure after 2^nd^ treatment with no recurrence after switching anesthesia induction agent to propofol.	FHR deceleration after 2^nd^ treatment, but non subsequently. Delivery of healthy full-term infant.
DeBattista et al. (47)	USA	Prenatal MDD, n=1	First trimester, n=1	Acute BT ECT, 5 treatments	Remission (HAM-D score improved from 31 at baseline to 7 following the 5^th^ treatment)	No significant maternal adverse events.	FHR deceleration after 4^th^ and 5^th^ treatments, each followed by rapid return to baseline. Delivery of healthy infant at 38 weeks EGA.
Dorn et al. (103)	USA	Prenatal BP-D, psychotic features, n=1	First trimester, n=1	Acute BT ECT, 9 treatments	Remission after 9 treatments	No significant maternal adverse events, though “mildly hypomanic” symptoms were described.^g^	No significant fetal or neonatal adverse events.
Echevarria Moreno et al. (48)	Spain	Psychotic depression, n=1	First trimester, n=1	Acute BT ECT, 9 treatments	Remission	Moderate memory loss for the time period around the acute ECT series. “Minimal” vaginal bleeding after the 2^nd^ treatment and “profuse” vaginal bleeding after the 3^rd^ treatment.	Miscarriage after the 3^rd^ treatment.
Erturk et al. (49)	Turkey	Prenatal depression with suicidal ideation, n=1	Second trimester, n=1	Acute BF ECT, 2x/week, 10 treatments	Remission	Not reported.	Delivery of healthy infant at 38 weeks EGA by cesarean section.
Forray & Ostroff (50)	USA	Mixed sample, 5 cases total PPD, psychotic features, n=2 PPP (bipolar I disorder), n=1 Postpartum BP-MX, n=1 Postpartum mood disorder, NOS, n=1	3 weeks to 11 months postpartum	Acute BT ECT, 6 to 9 treatments Continuation BT ECT, 4 to 11 treatments	Case 1 (postpartum mood disorder NOS) – Significant improvement by 3^rd^ treatment and eventual remission.^h^ Case 2 (PPP, bipolar I disorder) – “marked response” by 2^nd^ treatment and eventual remission.^h^ Case 3 (PPD with psychotic features) – significant improvement by 5^th^ treatment with eventual remission.^h^ Case 4 (BP-MX) – Significant improvement by 3^rd^ treatment and eventual remission.^h^ Case 5 (PPD with psychotic features) – significant improvement by 2^nd^ treatment with eventual remission.^h^	Transient memory disturbance in 3 of 5 cases.	Not reported.
Gahr et al. (51)	Germany	Prenatal depression, suicidal ideation, resistant to medication and L-DLPFC TMS, n=1	First trimester, n=1	Acute RUL ECT, 13 treatments	Remission (reduction in baseline BDI score [56] before ECT to 4 [1 week after final ECT treatment])	No apparent maternal complications during the acute ECT series. Unimpaired gestation at 24 weeks EGA (2 months after final ECT treatment).	No fetal trauma based on sonographic data.
Gannon et al. (52)	USA	Prenatal BP-D, passive suicidal ideation, n=1	Third trimester, n=1	Acute BT ECT, 7 treatments	Remission noted after delivery	Prolonged seizure, transient uterine contractions (first treatment only), nausea, mild headaches, transient urinary retention. Hypomanic symptoms^i^ while receiving ECT and taking sertraline and lurasidone, necessitating discontinuation of sertraline and increasing the dose of lurasidone.	Delivery of healthy infant at 38 weeks EGA.
Gonzales et al. (53)	USA	Depression with catatonia, n=1	Second trimester and in early postpartum, n=1^j^	Acute RUL ECT during the second trimester, 10 treatments Acute ECT during the early postpartum, electrode placement unspecified, 12 treatments	“Notable improvement” in mood and catatonic symptoms after 10 treatments initiated in the second trimester. Improvement in depressive symptoms with residual impoverished speech after 12 treatments (response plateaued after the 10^th^ treatment) given in the postpartum.	Not reported.	Delivery of healthy infant at 40^+1^ weeks.
Gressier et al. (13)	France	PPD, suicidal ideation, n=1	Approximately 12 weeks postpartum	Acute BT ECT, 29 treatments total	Remission, with improvement in HAM-D, QIDS-C, and EPDS scores from baseline (32, 28, and 23) to the end of ECT (3, 2, and 3). No depressive relapse at 6 month follow-up.	Not reported.	Not reported.
Griffiths et al. (54)	USA	Prenatal MDD, suicidal ideation, n=1	Second trimester, n=1	Acute BT ECT, 11 treatments total	Initial series of 6 ECT treatments (given between 23 and 26 weeks EGA) were provided “with good results.” Hospital admission was required at 28 weeks EGA, where 5 additional ECT treatments were provided over 3 weeks.	No abnormalities in VS, SaO_2_, or uterine activity	No abnormalities in FHR. Delivery of healthy infant at 40 weeks.
Grover et al. (55)^k^	India	Mixed sample, 13 cases total. PPD, n=3 PPD, psychotic features, n=4 Postpartum BP-D, n=1 Postpartum manic episode, n=2 Schizophrenia, n=2 “Acute and transient” psychotic disorder, n=1	For PPD and BP-D cases, 2 to 21 weeks postpartum.^k^	Acute ECT, electrode placement unspecified, 5 to 12 treatments for PPD and BP-D cases.	Remission for all PPD and BP-D cases based on post-ECT HAM-D score ≤ 7.	Body aches, memory disturbances	All babies were breastfed during the postpartum without observed or reported adverse effects.
Grover et al. (56)^l^	India	Mixed sample, 10 cases total. PPD, n=6 Postpartum manic episode, n=1 “Acute and transient” psychotic disorder, n=2 Organic psychosis, n=1	Mean duration of episode(s) at the time of ECT con-sideration was 3.8 months. Diagnosis-specific results were not provided.	Acute BT ECT, mean number of effective ECT treatments (all cases) was 6.7 (range 2 to 12). Diagnosis-specific results were not provided.	9 or 10 patients had at least a partial response to ECT (“overall improvement >50%”). Diagnosis-specific results were not provided.	“No immediate complications” during the ECT procedure. Two patients developed “delayed complications” (delirium, seizures).	Not reported.
Grover et al. (57)^m^	India	Mixed sample, 5 cases total. Prenatal depression, suicidal ideation, n=2 Manic episode, n=1 Schizophrenia, n=2	Second trimester, n=2	Acute BT ECT, 6 treatments were provided in both prenatal depression cases.	Remission in one prenatal depression case (78.6% reduction in HAMD score), partial response in the other prenatal depression case (65.7% reduction in HAMD score).	No ECT-related complications in either prenatal depression case.	Delivery of healthy infant (both prenatal depression cases). One delivery was by cesarean section at 35 weeks owing to PIH. The other was by NSVD at 38 weeks EGA.
Guillet et al. (58)	France	Prenatal depression, suicidal ideation, in patient with dopamine-responsive dystonia, n=1	Unspecified	Acute ECT, electrode placement unspecified, 13 acute treatments followed by consolidation treatments occurring once every 2 months.	“Clear improvement” in mood symptoms, dyskinesia, and dystonia after 13^th^ treatment	Not reported.	Not reported.
Gunduz et al. (59)^a^	Turkey	Prenatal MDD with psychotic features, n=1	Third trimester, n=1	ECT electrode placement unspecified, 4 acute treatments.	Not reported.	Transient uterine contractions that increased in intensity after each ECT treatment despite tocolytic therapy.	FHR fluctuations on continuous fetal monitoring (90 bpm to 140 bpm), each lasting about 5 minutes before returning to baseline. No delivery outcomes reported.
Herzog et al. (60)	USA	Mixed sample, 13 cases total. PPD, n=5 BP, n=5 Schizophrenia, n=3	Unspecified	Acute ECT was provided for three rapid cycling patients diagnosed with BP. ECT electrode placement was unspecified. Four to 8 treatments were provided in these cases.	“Good response” after 4–8 treatments.	Not reported.	Not reported.
Howe et al. (61)	UK	Psychotic depression, n=1	Third trimester, n=1	Acute BT ECT, 4 treatments	“Rapid and sustained recovery.” Was noted as being well after 2 years of follow up.	Not reported.	Not reported.
Isik et al. (62)	Turkey	Psychotic depression, n=1	Second trimester, n=1	Acute ECT, electrode placement unspecified, 6 treatments	Not reported.	TMJ dislocation after 2^nd^ treatment in the setting of a prior TMJ dislocation 4 years previously, leading to switch from plastic bite block to cotton bit block and no further complications.	Not reported.
Iwasaki et al. (63)	Japan	Prenatal depression, n=1	Second trimester, n=1	ECT electrode placement unspecified, 14 treatments	Gradual improvement reported.	Not reported.	Transient FHR decrease with propofol but not thiamylal anesthesia. Delivery of healthy infant. Normal development through first three years of life.
Reveles Jensen et al., (64)	Denmark	PPD, psychotic features, n=1	24 weeks postpartum	Acute BT ECT, 26 treatments	Improved depressive symptoms between admission (HAMD score 21) and hospital discharge (HAMD score 16).	Objectively documented improvement in red-green color-blindness. No other side-effects from ECT occurred.	Not reported.
Kasar et al. (65)	Turkey	Prenatal MDD with psychotic features, n=1	Third trimester, n=1	Acute BT ECT, 4 treatments	“Marked improvement” in depressive symptoms (and reduction in HAMD score from 47 to 15) was noted after the 3^rd^ treatment.	Onset of “birth pain” one day after the 4^th^ treatment.	Premature delivery at 34 weeks, with normal newborn development and normal development over the first 6 months of life.
Kisa et al. (66)	Turkey	PPD, n=1	Approximately 8 weeks postpartum	Acute BF ECT, 8 treatments	“Substantial improvement” in depressive symptoms after 8^th^ treatment.	Prolonged seizure terminated with midazolam during the 2^nd^ treatment, thought to be influenced by ciprofloxacin. No prolonged seizures after resuming ECT without ciprofloxacin.	Not reported.
Leite et al. (67)^a^	Portugal	PPD with catatonia, n=1	24 weeks postpartum	Acute ECT, electrode placement and treatment frequency unspecified	Remission of catatonic symptoms, “improvement” of depressive symptoms.	Not reported.	Not reported.
Levy et al. (68)	Australia	Mixed sample, 3 cases total PPD, suicidal ideation, n=1 BP-D, suicidal ideation, n=2	Unspecified	Acute RUL/UB ECT, 3x/week. Case 1–10 treatments. Case 2–20 treatments. Case 3–9 treatments.	Cases 1 and 2 had clinically significant improvement in depressive symptoms (EPDS scores 21–22 at baseline, 2–4 at discharge). Clinical outcome was unclear for Case 3.	Not reported.	Not reported.
Livingston et al. (69)	USA	Prenatal psychotic depression, n=1	Second trimester of twin pregnancy, n=1	Acute ECT, electrode placement unspecified, 8 treatments	After delivery, the patient’s psychiatric status deteriorated, requiring “multiple medications and regular ECT.”	Spontaneous preterm labor at 35 weeks EGA.	One episode of transient FHR deceleration during the 3^rd^ treatment. Preterm delivery at 35 weeks EGA. Twin A was diagnosed with transposition of the great vessels; however, died of postoperative complications after successful surgical repair. Twin B was diagnosed with anal atresia, a small sacral defect, and coarctation of the aorta.
Maletsky et al. (70)	USA	Mixed sample, 27 cases total. Prenatal MDD, n=7 Prenatal MDD, psychotic features, n=7 Prental MDD, catatonia, n=5 Unspecified catatonia, n=8	One pregnancy case was described in detail. Trimesters of pregnancy unspecified.	One pregnancy case was presented in detail and described two acute BT ECT series, the first occurring 3x/week over two weeks (6 treatments).	All four pregnant patients “showed marked improvement.” The case described in detail had a partial acute response and declined continuation ECT. She experienced a relapse at 4 weeks postpartum and “responded fully” to a second series of BT ECT.	Not described.	Delivery of a healthy infant.
Malhotra et al. (71)	India	Mixed sample, 2 cases total. Prenatal depression, suicidal ideation, n=1 Unspecified catatonia, n=1	Second trimester, n=2	Acute ECT, electrode placements and treatment frequencies unspecified.	Not reported.	No apparent complications.	Normal real-time US findings (pre- and post-procedure).
Martinez-Sosa et al. (72)	USA	Prenatal depression with catatonia, n=1	First trimester, n=1	Acute BT ECT, 7 treatments	Significant improvement after 4^th^ treatment and eventual resolution of mood and catatonic symptoms.	Premature labor/PPROM at 31 weeks	Normal spontaneous premature delivery at 31 weeks. Infant diagnosed remained hospitalized for first 50 days of life with hyaline membrane disease and prematurity associated apnea, retinopathy, and anemia.
May et al. (73)	USA	PPD, suicidal ideation, n=1	ECT offered during third hospitalization within the 1 year after delivery.	ECT electrode placement unspecified, 7 acute treatments followed by maintenance ECT.	Acute remission based on improvement in MADRS score from 45 to 9. Clinical response sustained while receiving maintenance treatments.	None described during acute ECT. Severe unilateral myalgias after one maintenance ECT treatment, “possibly due to insufficient paralysis.”	Not reported.
Morris et al. (74)^n^	Puerto Rico	PPD, suicidal ideation, n=1	Unspecified	Acute ECT, electrode placement and treatment frequency unspecified.	Full response achieved after several exacerbations of depressive symptoms	Headache	Not reported.
Mynors-Wallis et al. (75)	UK	Prenatal depression, catatonia, n=1	Third trimester, n=1	Acute ECT, electrode placement and treatment frequency unspecified.	“Good response.”	Not reported.	Not reported.
O’Reardon et al. (76)	USA	Prenatal MDD, suicidal ideation, n=1	Second trimester, n=1	Acute BT-BF ECT, 18 treatments, followed by 13 continuation ECT treatments over 6 months.	Positive response based on 50% improvement in HAM-D scores after 3^rd^ acute treatment. Depressive symptoms eventually remitted. Continuation treatments extended into the postpartum were effective.	Not reported.	Delivery of a healthy infant at 37 weeks by scheduled cesarean section. Normal development through 18^th^ month of life.
Ozgul et al. (77)	Turkey	Prenatal depression, suicidal ideation, n=1	First trimester, n=1	Acute BT ECT, 3x/week, 10 treatments	Not reported.	No significant hemodynamic changes.	Condition of the fetus was evaluated by obstetrician after each treatment, but outcomes were not reported.
Patel et al. (78)	USA	Prenatal MDD, suicidal ideation, n=1	Third trimester, n=1	Acute BT ECT, 3x/week, 8 treatments	Remission.	Transient asymptomatic contractions relieved with IV fluids.	Transient decreases in FHR.
Pesiridou et al. (79)	USA	Prenatal BP-D, borderline personality disorder, PTSD, SUDs, suicidal ideation, n=1	Third trimester, n=1	Acute RUL ECT, 6 treatments, followed by continuation ECT	Acute remission of suicidal ideations, “significant decreases” in depressive and anxious symptoms.	Disorientation, confusion, and memory disturbances after increasing stimulus strength owing to short seizures.° Painful contractions at 32 weeks EGA following 6^th^ treatment, responsive to tocolytics. Experienced periodic contractions until 37 weeks EGA.	Delivery of healthy newborn at 37 weeks EGA.
Pierre et al. (80)	France	Prenatal BP-MX, suicidal ideation, n=1	Second trimester, n=1	Acute BT ECT, 5 treatments	Depression scores (HAMD 25) “entirely improved” after ECT. YMRS scores improved from 26 to 3.	Uterine contractions after 1^st^ treatment.	Normal fetal development on ultrasound scanning. No development of fetal bradycardia.
Pinette et al. (81)	USA	Prenatal MDD, n=1	ECT administered throughout pregnancy	Maintenance ECT was continued into and throughout pregnancy	Good response to maintenance ECT was documented.	Induction of labor owing to pre-eclampsia at 37^+1^ weeks.	Small left cerebellar, bihemispheric deep white matter, and cortical infarcts on CT/MRI.
Rabie et al. (82)	USA	Mixed sample, 5 cases total. Prenatal depression, n=3 (1 with suicidal ideation) Prenatal BP/BP-D, n=2 (1 with suicidal ideation)	Trimesters at ECT initiation unspecified.	Acute RUL ECT administered with continuous FHR monitoring, range 2 to 23 treatments	All patients reported improvement, one with “limited improvement” and another rehospitalized due to depressive relapse.	Headaches, muscle soreness, nausea, fatigue, memory disturbances, transient uterine contractions.	Reassuring FHR tracings before and during ECT (data on 32 of 34 treatments that included continuous FHR monitoring). Transient decelerations noted for 4 treatments, none requiring intervention or additional monitoring. Healthy term deliveries in all 5 cases.
Ratan et al. (83)	UK	Perinatal depression with psychotic features, n=1	Postpartum week(s) unspecified	Acute ECT, electrode placement and frequency of treatment unspecified.	Remission	Not reported.	Not reported.
Ray-Griffith et al. (84)	USA	Mixed sample, 8 cases total Prenatal depression, n=3 Prenatal depression, suicidal ideation, n=2 Prenatal BP-D, n=1 Prenatal BP-MX with suicidal ideation, n=1 Unspecified prenatal mood disorder with suicidal ideation, n=1	Second trimester, n=5 Third trimester, n=3	Acute RUL ECT, 30 treatments total, ranging from 1 to 7 treatments individually.	Remission of suicidality in 5 patients who presented with acute suicidal ideation. “Clinical improvement” of depression for 6 of 8 patients.	Case 5 - ECT discontinued due to treatment-emergent hypomanic symptoms after the 1^st^ treatment at 21^+2^ weeks. PPROM/preterm labor at 30^+1^ weeks. Case 8 – ECT stopped after asymptomatic episode of complete heart block requiring atropine and intensive care observation, deemed secondary to anesthesia with methohexital.	Uncomplicated term deliveries except for Cases 3 and 8 (did not deliver at investigators’ institution) and Case 7 (below). Case 7 – infant born with right club foot and right 5^th^ toe displacement, detected on ultrasound before ECT.
Repke et al. (85)	USA	Prenatal depression with psychotic features, n=1	Second trimester, n=1	Acute ECT, electrode placement unspecified, 5 treatments	Positive response	Reduction in blood pressure, though secondary to low intravascular volume, prevented with pre-hydration during treatments 2-5.	Delivery of healthy infant.
Richardson et al. (86)^a^	UK	Prenatal BP-MX, n=1	Third trimester, n=1	ECT electrode placement unspecified, 8 treatments. ECT resumed on a Q 2 week basis following delivery, then extended to monthly maintenance ECT.	Positive acute response and positive maintenance response at 6 months postpartum.	No maternal complications.	No fetal complications. Delivery at 38 weeks EGA by elective cesarean section.
Rineh et al. (87)	Iran	Prenatal MDD, n=1	Third trimester, n=1	Acute ECT, electrode placement unspecified, 3x/week, 6 treatments.	Significant improvement in depression after 3^rd^ treatment and remission after the 6^th^ treatment.	Intermittent uterine contractions, eventually requiring prophylactic magnesium sulfate	Single transient FHR reduction after 2^nd^ treatment.
Sahan et al. (88)	Turkey	Prenatal depression with psychotic features, catatonia, and urinary bladder overdistension, n=1	First trimester, n=1	Acute ECT, electrode placement unspecified, 3x/week, 7 treatments	Patient was able to urinate after 3^rd^ treatment. Negativism resolved. Discharged from the hospital with full recovery. Experienced relapse during third trimester, managed with pharmacotherapy.	Not reported.	Delivery of healthy infant, with normal development through first year of life.
Salzbrenner et al. (89)	USA	Prenatal BP-D with history of IVF and pre-eclampsia, suicidal ideation, n=1	Third trimester, n=1	Acute BT ECT, electrode placement unspecified, 3x/week, 9 treatments	Positive response, improvement in MADRS score from 32 at baseline to 12 after 8^th^ treatment. Psychiatrically stable at 9 month follow-up.	Hypertensive responses to ECT controlled with IV labetalol initially (switched to remifentanil). ECT had to be stopped owing to cognitive side effects (MMSE 19/30 after 9^th^ treatment; 30/30 at baseline). MMSE scores steadily improved to 30/30 over the following 4 months.	No fetal complications. Delivery of healthy infant at 38 weeks EGA by cesarean section. Normal development through first 9 months of life.
Sandal et al. (90)	Turkey	Prenatal depression, n=1	Second trimester, n=1	ECT electrode placement unspecified, 6 treatments	Not reported.	Not reported.	Infant diagnosed with Mobius syndrome, not believed to be caused by ECT.
Sarma et al. (91)	Australia	PPD with suicidal ideation and type 1 Chiari malformation, n=1	8 weeks postpartum	Acute BF ECT, 3x/week, reduced in frequency to 2x/week after 2 weeks, totaling 12 treatments	Remission of suicidal ideation and significant improvement in depressive symptoms (MADRS and EPDS 32 and 23 at baseline, 11 and 4 after ECT). Mild reduction in MoCA scores (from 29 to 25).	Mild headaches, concentration and memory disturbances.	Not reported.
Serim et al. (92)	Turkey	Prenatal depression with psychotic features, n=1	Third trimester, n=1	Acute BT ECT, 3x/week, 10 treatments	“Significant” improvement after 5^th^ treatment. Relapse occurred 2 weeks after 10^th^ acute treatment, eventually responsive to medication only.	Brief uterine contractions after one treatment that was responsive to tocolytic therapy.	Brief (2–3 second) FHR deceleration during one treatment. Delivery of healthy infant at 39 weeks by cesarean section.
Shea et al. (93)	USA	PPD with psychotic features in patient with Turner syndrome, n=1	4 weeks postpartum	Acute BT, 6 treatments	Resolution of infanticidal thoughts, “steady” improvement of depressive/neurovegetative symptoms.	Not reported.	Not reported.
Sherer et al. (94)	USA	Prenatal depression with psychotic features, n=1	Third trimester, n=1	Acute BT, 7 treatments	Not reported.	Transient uterine contractions (eventually requiring tocolytic therapy), vaginal bleeding, abruptio placentae diagnosed after delivery with unremarkable postoperative course.	Delivery of healthy infant at 37 weeks by cesarean section.
Strain et al. (95)	USA	Diagnosed postpartum depression presenting acutely with psychotic features and catatonia, n=1	5 months postpartum	Acute ECT, electrode placement unspecified, 6 treatments	Positive response to ECT, with early improvement noted after the 1^st^ treatment. Stable at 18 month follow up.	Not reported.	Not reported.
Takubo et al. (96)	Japan	PPD, suicidal ideation, n=1	Postpartum week(s) unspecified	ECT electrode placement and frequency of treatment unspecified.	Partial response.	Not reported.	Not reported.
Walker et al. (97)	USA	Prenatal depression with psychotic features, n=1	Second trimester of twin pregnancy, n=1	Acute BT ECT, 6 treatments initially, 7 treatments after relapse. Two continuation treatments given once-weekly.	Remission of presenting symptoms after initial acute series, followed by relapse 3 weeks later. This was followed by 7 additional treatments that resulted in remission. Two weekly continuation treatments were given.	Occasional uterine contractions on tocodynamometry	Twin B – nonreactive NST after 2^nd^ treatment with normal contraction stress test and normal subsequent NSTs. Delivery at 35 weeks EGA. Twin A - transposition of the great vessels, death 2 weeks after surgery. Twin B- born with imperforate anus, hemivertebra of the sacral vertebra, atrial septal defect, and coarctation of the aorta.
Watanabe et al. (98)	Japan	Prenatal MDD, suicidal ideation, n=1	Third trimester, n=1	Acute BT ECT, 2x/week, 6 treatments	Partial response (improvement in HAM-D score from 36 to 26) with resolution of suicidal ideation and restoration of appetite.	Uterine contractions from the 3^rd^ treatment onward	Persistent fetal tachycardia (180–200 bpm >30 minutes), presumed secondary to maternal apnea during ECT, with no recurrence after the reinitiation of oxygenation just after electrical stimulus delivery. Delivery of healthy infant at 38 weeks EGA.
Wise et al. (99)	USA	Prenatal depression with psychotic features, n=1	Third trimester, n=1	Acute RUL ECT, 8 treatments	Remission after the initial acute series, with good response to 4 additional treatments to address return of depressive symptoms that occurred twice before delivery.	One brief episode of SVT that required no intervention.	Oxytocin-induced vaginal delivery of healthy infant at 37 weeks EGA, with normal development through first 10 months of life.
Yamada et al. (100)^a^	Japan	Prenatal BP-D, n=1	Third trimester, n=1	ECT electrode placement unspecified, 2-3x/week, 10 treatments	Unspecified improvement in depressive symptoms.	FGR diagnosed by ultrasound, with fetal growth delay starting at 29 weeks EGA, possibly caused by thrombosis of the umbilical vein.	FGR-delivery by emergent cesarean section at 32 weeks.
Yang et al. (101)	S. Korea	Prenatal BP-D with psychotic features, n=1	Third trimester, n=1	Acute ECT, electrode placement unspecified, 7 treatments	Positive response to initial acute series followed by relapse 3 weeks after hospital discharge.	Transient uterine contractions/possible preterm labor, responsive to tocolytic therapy, with no apparent reoccurrence.	FGR fetus diagnosed before ECT initiation. Premature delivery at 35^+4^ by emergency cesarean section. Infant was diagnosed with hyaline membrane disease and congenital hypertrophic pyloric stenosis.
Ying et al. (102)^a^	Unspecified	Prenatal depression, suicidal ideation, n=1	Third trimester, n=1	Acute ECT, electrode placement and frequency of treatments unspecified.	Resolution of depression.	Prolonged neuromuscular blockade that delayed extubation, due to pseudocholinesterase deficiency owing to pregnancy. Rocuronium replacement of succinylcholine allowed completion of ECT series.	Prolonged transient FHR deceleration after 1^st^ treatment.

Key: BP, bipolar spectrum disorder; BP-D, bipolar depression; BP-M, bipolar mania; BP-MX, bipolar mixed episode; BF, bifrontal ECT; BT, bitemporal ECT; BZD, benzodiazepine; CGI, Clinical Global Impression scale; CGI-S, Clinical Global Impression severity subscale; CT, computed tomography scan; DPHD, diphenylhydantoin; EGA, estimated gestational age; EPDS, Edinburgh Postnatal Depression Scale; GFR, fetal growth restriction; FHR, fetal heart rate; IV, intravenous; IVF, *in vitro* fertilization; L-DLPFC, left dorsolateral prefrontal cortex; MADRS, Montgomery Asberg Depression Rating Scale; MDD, major depressive disorder; MMSE, Folstein Mini-Mental Status Examination; MoCA, Montreal Cognitive Assessment; MRI, magnetic resonance imaging scan; MV, mechanical ventilation; NST, non-stress test; NSVD, normal spontaneous vaginal delivery; PIH, pregnancy induced hypertension; PPD, postpartum depression; PPROM, preterm premature rupture of membranes; PTSD, posttraumatic stress disorder; QIDS-C, clinician-rated Quick Inventory of Depressive Symptomatology scale; RUL, right unilateral ECT; RUL/UB, right unilateral ultrabrief pulse ECT; SE, status epilepticus; SUD, substance use disorder; SVT, supraventricular tachycardia; TMS, transcranial magnetic stimulation; UK, United Kingdom; USA, United States of America.

^a^Citation is from a published conference abstract. ^b^Case series described ECT in 33 pregnant patients, 19 with MDD, 12 with BP, and 2 with schizophrenia. Among all 33 cases, ECT was performed starting in the first, second, and third trimesters for 13, 15, and 5 patients, respectively. ^c^Mood polarity was unspecified. ^d^A total of 68 cases were presented, including 43 patients with “unipolar depression,” 20 with a bipolar spectrum disorder, 3 with obsessive-compulsive disorder, and 2 with schizophrenia. ^e^This 29-year-old patient was subsequently found to have osteoporosis based on a dual-energy X-ray absorptiometry (DEXA) scan z-score of -2.5. ^f^This was a single case report of ECT for PPD in a patient who attended a day center program for individuals with learning disabilities. ^g^“Mildly hypomanic” symptoms were observed for 1 week after the final (9^th^) ECT treatment that included being “talkative, with elevated mood.” Maintenance ECT was planned. ^h^All 5 cases were described as achieving remission of acute symptoms with no subsequent relapses in 4 subjects who received continuation ECT treatments. ^i^Hypomanic symptoms included increased energy, increased goal-directed behavior, and “slight” decreased need for sleep after the 4^th^ treatment. There was “mild mood elevation” noted after hospital discharge. Euthymia was documented within 2 weeks following delivery. ^j^This patient received an acute inpatient series of RUL ECT for depression with catatonic features while pregnant; however, 2 months after discharge, she was hospitalized with a relapse of depressive and catatonic symptoms. Labor was induced at 40^+1^ weeks. ECT was resumed during the early postpartum. ^k^A total of 13 postpartum ECT cases are described consisting of a mixed sample of patients with PPD (7 cases), BP (3 cases total, 1 with BP-D), schizophrenia (2 cases), and “transient psychotic disorder” (1 case). The mean total duration of psychiatric symptoms during the postpartum period in the entire case series was 57.8 days, or 8.3 weeks. ^l^Ten postpartum ECT cases are summarized, composing a mixed sample of patients with PPD (6 cases), acute mania (1 case), PPP without underlying diagnosis specified (2 cases), and “organic psychosis” (1 case). ^m^Mixed sample included 2 cases of recurrent prenatal depression, 1 case with BP-M, and 2 cases with schizophrenia. ^n^This was a first-person account of having undergone ECT for PPD. ° Switching anesthesia induction agent from methohexital to etomidate resulted in enhanced seizure durations.

As shown in **Figure 2C**, most reviewed papers described acute-phase mECT while just 10 reports described continuation- or maintenance-phase treatment, with or without an acute phase (37, 39, 46, 50, 73, 76, 79, 81, 86, 97). The most common indication(s) for mECT, when specified, were treatment resistance (n=31) followed by psychotic symptoms/features (n=21) and high suicide risk (n=21). Indications for mECT were unspecified in 14 reports. Nearly all the 30 reports that addressed treatment-resistant depression defined treatment resistance as poor response to prior treatments (n=29), including one TMS-resistant case (51). One report identified intolerance of medications as the principal indication for mECT (35). Fourteen reports described mECT for catatonia, including 3 reports that explicitly identified benzodiazepine-resistant catatonia as the intended indication (67, 72, 95). ECT was the preferred treatment or treatment because of pregnancy in 10 reports (34, 37, 39, 52, 54–56, 66, 70, 97).

In terms of obstetric information, most reports described mECT in the setting of pregnancy (n=64) while 23 reports included cases of postnatal mECT delivery, with or without a prenatal treatment phase. When gestational information was provided, nearly all such reports involved singleton pregnancies/deliveries, whereas 2 reports included twin pregnancies/deliveries (69, 97). The pre-ECT use of *in vitro* fertilization was reported in one case (89).

General medical comorbidity was often not reported. For example, of the 9 observational studies, two provided information on the frequency of diabetes and one provided details on the frequency of underweight, overweight, and obesity based on BMI ranges (24, 26, 32). Only limited information on comorbid conditions was available from individual case reports and small case series. However, 15 such reports documented comorbid conditions including obesity, non-gestational hypertension, gestational and non-gestational diabetes, hyperthyroidism, chronic musculoskeletal pain syndromes, migraine headache, congenital neurological diseases, and others. Acute injuries (skeletal fractures) and intentional poisonings (acetaminophen toxicity) related to suicide attempts were described in three reports (13, 50, 61), while anorexia, weight loss, or other conditions related to nutritional compromise in severely depressed patients were documented five reports (45, 49, 62, 70, 99).

Medications taken on or around the time of ECT administration was more thoroughly documented than medical comorbidities. Summary data on the frequency of concurrently prescribed antidepressants, mood stabilizers, antipsychotic drugs, or benzodiazepines were provided for six of nine observational studies. Forty-nine of the 73 individual case reports and small case series included information on individual drugs falling within these same broad categories. In 8 reports, only past failed medication trials were reported (35, 60, 67, 69, 70, 75, 86, 103). The discontinuation of psychotropic medications during or in anticipation of pregnancy or the absence of psychotropic medications at the time of ECT was specified in 6 reports (44, 46, 47, 54, 66, 89).

### 3.3 ECT treatment characteristics

Of the 56 reports where electrode placement was clearly described, the most common types of ECT electrode placement were bitemporal (34 reports) and right unilateral (12 reports) using a brief pulse width. The use of right unilateral ultrabrief pulse ECT was described in 3 reports (35, 68, 82) and bifrontal ECT was described in 9 reports (13, 28, 30, 31, 41, 49, 66, 77, 81). The number of weekly sessions of acute ECT was often not provided in the reviewed reports. When the number of weekly sessions was specified, thrice-weekly ECT sessions were most described (25, 51, 52, 65, 68, 71, 78, 88, 89), although twice-weekly ECT sessions were also reported (49, 80). Information on ECT stimulus parameters in conjunction with electrode placement was provided in only 27 reports. For summary dose metrics, stimulus train energy values ranged from 29.6 J - 43.6 J, 124.7 mC - 436 mC, or 10% - 75% of maximal charge. Individual ECT dose elements from 23 individual reports included current amplitudes (170 V or 500–842 mA), pulse widths (0.5 msec - 1.6 msec), pulse or pulse-pair frequencies (20 Hz - 90 Hz or 125 pulses/sec), and stimulus train durations (1 sec - 8 sec). Seizure threshold titration or determination was mentioned in 6 reports; however, the exact parameters at each step were not specified (25, 26, 34, 68, 79, 80, 90).

### 3.4 Anesthesia technique

The most common anesthetic induction agents in the reviewed papers were propofol (21 reports), thiopental (16 reports), and methohexital (14 reports). One report described the use of ketamine augmentation of propofol anesthesia for ECT in the setting of third trimester pregnancy in hopes of enhancing antidepressive efficacy (78). Among the 9 observational studies, only one provided complete information on the anesthetic (thiopental 3–4 mg/kg) and neuromuscular blocking agent (succinylcholine 0.5-0.75 mg/kg) with doses (25). From case reports and small case series, 26 included complete information on anesthetic and neuromuscular blocking agents. Specifically reported anesthetic drugs (with dose ranges in total mg administered or mg/kg infused) included methohexital (50–170 mg or 1 mg/kg), propofol (140 mg or 1 mg/kg), thiopental (100–300 mg or 3–4 mg/kg), thiamylal (4 mg/kg), succinylcholine (40–120 mg or 1–2 mg/kg). However, anesthetic doses, neuromuscular blocking drugs, airway management techniques, pharmacological adjuncts to anesthetic induction agents, and modifications of anesthetic technique for perinatal safety were unspecified in most reports. In one case report, methohexital was switched to propofol to shorten seizure length after a prolonged ECT seizure that led to an episode of fetal bradycardia (46). Another case report referenced switching from thiopental to etomidate to increase seizure duration and based on “favorable experiences with the drug in obstetrical patients” (47). A third case report described adjusting the dose of methohexital in response to seizure duration and quality but not perinatal safety reasons (79).

### 3.5 Adaptations to standard ECT technique

Specific maternal and fetal monitoring procedures to accommodate pregnancy or postpartum status were described in fewer than half of the reviewed reports. Still, as shown in **Supplementary Table S3**, several adaptations to standard ECT technique were documented in case reports and case series. Twenty-three reports provided details on airway management techniques, including 12 that used endotracheal intubation during second- or third-trimester pregnancies and 10 reports of mask airway. One case report described the use of a supraglottic airway for subsequent ECT treatments due to difficult intubation under direct laryngoscopy during her first treatment at 20 week’s gestation (40). When described, maternal monitoring techniques included tocometry/tocodynamometry, ultrasound assessments, obstetrician attendance during the procedure, and ready access to emergent cesarean section capabilities in specific cases (**Supplementary Table S3**). Additional precautions applied in later stages of pregnancy (after the first trimester) included elevation of the right hip to prevent aortocaval compression, cricoid pressure to reduce the risk of regurgitation and aspiration of stomach contents, pre-hydration/pre-oxygenation, and endotracheal intubation. Fetal heart rate monitoring, non-stress tests/biophysical profiling, and ultrasonography for fetal morphology, fetal heart rate and uterine contractility monitoring were also described (**Supplementary Table S3**).

### 3.6 Effectiveness

Main effectiveness results from observational studies and from case series/reports are outlined in **Table 1** and **Table 2**, respectively. Treatment effects were reported as categorical outcomes (e.g, remission, partial response, non-response, hospital readmission, etc.) in 35 (42.7%) reports, as continuous outcomes (absolute or relative change in rating scale scores) in 16 (19.5%) reports, and as narrative descriptions of outcomes (“clear,” “full,” or “complete” response, unspecified improvement in mood symptoms, etc.) in 17 (20.7%) reports. The most used rating scales were the Hamilton Depression Rating Scale (HAM-D, 12 reports), the Clinical Global Impression scale (-severity [CGI-S] or -improvement [CGI-I] subscales, 8 reports), the Montgomery Asberg Depression Rating Scale (MADRS, 5 reports) (104), the Bush Francis Catatonia Rating Scale (BFCRS, 3 reports) (105), and the Quick Inventory of Depressive Symptomatology (QIDS) (106) and the 9-item Public Health Questionnaire (PHQ-9, 1 report each) (107). Therapeutic outcomes were not assessed in 12 reports. One study each focused only on descriptions of interventions provided on a specialized mother-baby unit (32) and on qualitative outcomes (108).

#### 3.6.1 Observational and chart review studies

##### 3.6.1.1 ECT for perinatal vs non-perinatal mental health disorders

Controlled investigations of acute responses to mECT for perinatal depression collectively involved a variety of comparisons, mainly involving ECT for perinatal mental health conditions vs ECT for non-perinatal mental health disorders. For example, a retrospective cohort study of 793 pregnant patients identified in linked registers, including a population-based ECT registry, documented numerically higher positive response rates (CGI-I scores of 2 [much improved] or 1 [very much improved]) with ECT in patients with a perinatal psychiatric diagnosis than with ECT in non-pregnant patients with a psychiatric diagnosis (74% vs 65%) who were matched on propensity scores (24); however, between-group differences in response rates were not statistically significant. Using the same population-based ECT registry, significantly higher rates of response (using the definition above, assessed within 7 days after receiving ECT, 87.0% vs 73.5%) and remission (CGI-S scores of 2 [borderline ill] or 1 [not ill], 45.4% vs 29.9%) were also observed in patients who received ECT for PPD or postpartum psychosis than patients who received ECT for a non-postpartum indication (31).

In another retrospective cohort study, a population-based ECT database and records from an inpatient mother-baby unit were used to identify a cohort of severely depressed patients with postnatal depression/psychiatric disorders (baseline mean MADRS score 43.1, n=12) and patients who received ECT for non-perinatal psychiatric disorders (baseline mean MADRS score 41.3, n=23) (27). After ECT, the mean reduction in MADRS scores was 10.1 points greater for perinatal depression cases than non-perinatal controls (-30.8 vs. -20.7, p=0.06). At baseline, the proportion of individuals with severe depression (based on MADRS scores) was 83% in both the perinatal ECT group and the non-perinatal ECT group. After ECT, the proportion of severe depression cases was 8% in the perinatal ECT group and 22% in the non-perinatal ECT group.

In a university hospital register-based study, clinical recovery ratings (based on a 4-point Likert scale) were compared between patients who received ECT for puerperal mental health conditions and patients (<45 years of age) who received ECT for non-puerperal mental health conditions (29). Complete records were available for 114 of the 137 patients who received ECT, including 58 patients in the puerperal ECT group and 56 patients in the non-puerperal group. Analyses restricted to patients with depression showed a significantly higher proportion of patients rated as either “asymptomatic” or having achieved “marked improvement” in the puerperal depressed group than in the non-puerperal depressed group at the end of ECT (66.7% vs 27.3%, p<0.001) and at reassessment one month after ECT (61.9% vs 24.2%, p=0.003).

##### 3.6.1.2 Comparisons of bitemporal and bifrontal ECT

One study retrospectively compared the effects of bitemporal (BT, n=18 patients) and bifrontal (BF, n=13 patients) ECT in a mixed cohort of 31 patients (28). Edinburgh Postnatal Depression Scale (EPDS) and CGI-S scores were compared between groups at hospital discharge. Mean numerical pre- and post-treatment EPDS and CGI-S scores were not provided; however, post-treatment EPDS scores were reported as being significantly lower with BF than BT ECT (p=0.004). CGI-S scores were reported to be lower with BF than BT ECT at the level of statistical trend (p=0.06).

##### 3.6.1.3 ECT vs no ECT

Another study prospectively compared clinical responses in a mixed cohort of 78 patients with postpartum psychosis (24 with depression) who received acute ECT and those with similar indications who did not receive ECT (25). Data were analyzed for the entire cohort, without diagnosis-specific results. Psychopathology was assessed in all 78 patients, 34 of whom received ECT, using the Comprehensive Psychopathological Rating Scale (CPRS) (109). CPRS scores were similar between both groups at baseline (ECT 41.8 vs. no ECT, 39.5) and were similarly improved in each group at the end of follow-up (ECT, 4.5, no ECT, 4.2). Duration of admission was lower in the ECT group (19 days vs 23 days); however, there were no significant between-group differences in therapeutic outcomes or hospital lengths of stay.

##### 3.6.1.4 Relapse and rehospitalization after acute ECT

Other reports focused on relapse rates after an acute response to ECT, with relapse generally defined as rehospitalization for a psychiatric indication. For example, in a retrospective cohort study, rates of relapse (defined as rehospitalization for psychiatric reasons or suicide) were lower for patients who received ECT for PPD or postpartum psychosis (n=180) after 6 months (28% vs 39%), 1 year (31% vs 50%), and 2 years (40% vs 55%), as compared with control patients (n=180) who were <46 years of age and received ECT for a non-postpartum indication (30). The mean time to relapse (621 ± 548 days vs 440 ± 475 days) was numerically higher and the risk of relapse was significantly lower for patients who received ECT for PPD or postpartum psychosis (vs control patients) in unadjusted analyses (HR 0.61 [0.45, 0.83]). Statistical differences in relapse risk were nearly significant after adjusting for education level, unemployment, selected comorbidities, CGI-I score one week after treatment, drug treatment, and prior psychiatric admission history (HR 0.72 [0.52, 1.00]).

In a subsequent population-based study, linked registers were used to identify a mixed cohort of 91 patients with evidence of postpartum mood or psychotic disorders and no prior mood or psychotic disorder diagnoses and no history of ECT prior to giving birth (26). Cohort members were all psychiatrically hospitalized within the 6 weeks following delivery, 43% of whom were diagnosed with unipolar depression, 19% with bipolar disorder, and 38% with an unspecified psychotic disorder. A total of 17 (18.7%) patients received ECT. Rehospitalization (readmission to a psychiatric hospital within 6 months of the index hospital admission discharge date) occurred in 28 (30.8%) of the cohort members after a mean post-discharge interval of 16 days; however, ECT-specific results were not reported, nor were they compared with non-ECT treatment outcomes.

#### 3.6.2 Case reports and case series

As shown in **Table 2**, all but 11 case reports or case series provided details on therapeutic outcomes of mECT. Most cases focused on acute-phase treatment, while 11 reports described continuation or maintenance ECT outcomes. Positive therapeutic responses to mECT were documented in 60 (84.5%) case reports or case series; however, as noted earlier, there were disparate methods for describing treatment outcome. For instance, positive treatment outcomes were narratively described (without the use of psychopathology measures) as “remission” (11 reports); “complete response,” “resolution” of symptoms,” or “recovery” (7 reports); “improvement” or “partial response” (20 reports); “good response” or “good results” (3 reports); or “well controlled” after ECT (1 report). Improvements in specific symptoms were narratively described in 2 reports. When psychopathology measures were used in single and double case reports, remission was documented in 6 reports (based on HAM-D score <7, QIDS-C <5, MADRS <6, PHQ-9 ≤4, Beck Depression Inventory <9, or CGI-S <2), response was documented in 1 report (based on a 50% improvement in 24-item HAM-D score), and non-response was documented in 2 reports (<50% improvement in 24- and 17-item HAM-D scores).

Patient follow-up was usually confined to the acute treatment phase, which often ended at hospital discharge. Several reports documented recurrences of psychiatric illness after an initial acute treatment series resulting in rehospitalization, with or without additional acute mECT treatments (54, 70, 82, 88, 92, 97, 101). When timelines were provided, symptom relapses were noted to occur within 2–3 weeks following the final ECT treatment or hospital discharge. The extension of a positive acute phase response with continuation or maintenance treatments was documented in 2 reports (73, 76). In one case, additional mECT treatments were provided to address two depressive recurrences following initial symptomatic remission in a patient who required prolonged hospitalization for high-risk pregnancy (99).

### 3.7 Adverse maternal, fetal, and neonatal events

#### 3.7.1 Observational and chart review studies

Two observational and one chart review study reported safety or tolerability results in patients who received mECT for perinatal depression (and other indications in mixed cohorts). The largest of the three was a previously reviewed retrospective cohort study that used linked population-based registers to compare response rates in ECT-treated patients with a perinatal psychiatric diagnosis, non-ECT pregnancies from the same group of ECT-treated patients (non-ECT additional pregnancy group), and psychiatrically ill non-pregnant patients who received ECT (24). Control groups were matched with the main exposure group using propensity scores. Registry data included reports for specific complications including preeclampsia, diabetes, congenital malformations, stillbirth, Apgar scores (at 1-, 5-, and 10 minutes), birthweight, large/small for gestational age (LGA/SGA) status, and other maternal and neonatal complications. Most complications, including fetal malformations, LGA deliveries, and SGA deliveries, were comparable across exposure groups. As shown in **Table 1**, the risks of premature delivery and low 5-minute Apgar scores (< 7) were significantly higher for the pregnant ECT group than the non-ECT pregnant inpatient group, but not the non-ECT additional pregnancy group. Stillbirths occurred in 2 (2.1%) pregnancies in the pregnant ECT group, 1 (0.3%) pregnancy in the non-ECT pregnant inpatient group, and 1 (1.1%) pregnancy in the non-ECT additional pregnancy group.

A retrospective chart review study of 31 patients who received either BT or BF ECT during the prenatal (4 patients) or postpartum (27 patients) periods was presented as a conference abstract (28). Indications for ECT were described as “severe” cases with clinical diagnoses unspecified bipolar spectrum disorder (n=14), unspecified depression (n=3), unspecified psychotic disorder (n=4), unspecified postpartum psychosis (n=4), and “acute transient psychosis” (n=6). Maternal adverse effects of ECT included headache and cognitive difficulties. Fetal and neonatal safety outcomes, however, were not reported.

Preliminary maternal adverse effects and lactational safety data with ECT were provided in a prospective study 78 hospitalized patients with postpartum psychosis, 34 of whom received ECT (25). Clinical diagnoses assigned to cohort members included unspecified depression (n=24), acute manic episodes (n=32), and “non-affective” psychoses (n=22). The most common maternal adverse effects associated with ECT were memory disturbances (anterograde amnesia) in 6 (17.6%) patients and prolonged seizures in 4 (11.7%) patients, the latter managed with additional doses of sodium thiopental. Fifteen ECT-treated patients were hospitalized with their infants and continued breastfeeding without complications or apparent adverse effects.

#### 3.7.2 Case reports and case series

##### 3.7.2.1 Maternal and obstetric adverse events

As shown in **Table 2**, several reports documented common adverse effects known to be associated with ECT, including headache, nausea, myalgias, transient arrhythmias, hypertensive responses to ECT, cognitive disturbances, and post-ECT confusion. The most frequently reported perinatal adverse effects in patients were transient uterine contractions (14 reports), nearly all of which were transient (did not progress to preterm labor), although some required tocolytic therapy or prophylaxis. There were 5 reports of preterm labor with preterm deliveries occurring between 30^+1^ and 35^+4^ weeks EGA. Prolonged seizures were described in 4 reports, including one case of status epilepticus requiring aggressive doses of anesthetic medications to achieve seizure control, resulting in severe hypotension, the need for pressor support, and fetal demise (34). Other reported adverse events included pelvic pain, blood pressure reduction (owing to low intravascular volume), fetal growth restriction (presumed secondary to umbilical vein thrombosis), and prolonged neuromuscular blockade from administration of succinylcholine in a patient with pseudocholinesterase deficiency. Eleven reports specified no maternal complication with mECT, while information on adverse effects of mECT was not provided in 19 reports. There was one case of confirmed placental abruption diagnosed after Cesarean delivery at 37 weeks in a 35-year-old patient with severe depression and panic attacks who experienced the onset of regular uterine contractions, subsequent hypertonic-tetanic contractions, and blood pressure elevations followed by transient uterine bleeding during mECT at 34 weeks (94).

##### 3.7.2.2 Fetal/neonatal adverse events

The most frequently reported fetal/neonatal adverse events occurring on or around the time of ECT administration were fetal heart rate decelerations (10 reports), the majority of which were transient and without long-term impact on fetal or delivery outcomes. There were 7 cases of premature deliveries occurring between 31- and 35-weeks’ gestation, 5 of which also included preterm labor occurring days (3 reports) to weeks (3 reports) following ECT treatment. In general, numerous risk factors for premature delivery were present including severe mood disorder symptoms (all cases), twin pregnancy (2 cases), infectious disease complication during pregnancy (1 case), substance exposures (1 case), food refusal or weight loss during pregnancy (2 cases), maternal age >35 years (1 case), and threatened abortion in the current pregnancy prior to ECT (1 case). In one case, preterm labor occurred 11 weeks after receiving just a single ECT administration (84). There were 6 reports of congenital malformations. Specific congenital defects included cardiovascular (atrial septal defect, coarctation of the aorta, transposition of the great vessels), musculoskeletal (equinovarus foot deformity, congenital hip dysplasia, hemivertebrae, fifth toe displacement), and gastrointestinal (anal atresia). The two reports documenting adverse events in ECT-treated patients with twin pregnancies involved multiple co-occurring congenital malformations and the neonatal demise of one twin, each, following subsequent surgeries (69, 97). There were two reports of fetal death/stillbirth (one occurring in the setting of severe drug-resistant status epilepticus and another from an undetermined cause) and one report of first trimester miscarriage occurring shortly after a third acute ECT treatment. Direct causal links between ECT and these adverse events were unclear. Normal deliveries or child development outcomes were specified in 21 reports, while no information on these outcomes was provided in 9 reports.

## 4 Discussion

The current report presents an updated scoping review of the literature describing the broad effectiveness and maternal, fetal, and neonatal safety of mECT for perinatal depression as well as details on mECT technique and technical adaptations that may bear on its safety or effectiveness in that population. We abstracted information from 82 reports that included information on over 1,300 pregnancies or deliveries. Our review was limited to mainly individual cases and case series, which accounted for 85% of the reviewed reports, with only a handful of controlled observational studies. The collected literature described a broad spectrum of effectiveness and safety outcomes with predominantly acute mECT for multiple forms of perinatal depression that presented across multiple trimesters of pregnancy and in the postpartum. As with non-puerperal depression, mECT appears to confer rapid benefit for depressive, psychotic, and catatonic symptoms in severely depressed perinatal patients including those with treatment-resistant illness. Reported adverse events, many with uncertain etiologic links to the procedure itself, are discussed further below.

To our knowledge, there have been 7 systematic reviews of the safety and/or effectiveness of ECT administered during the perinatal period (110, 111 12–15, 17). Most reviews focused on ECT effects during pregnancy and generally supported ECT as a rapidly beneficial alternative to conventional antidepressive medications, particularly for pharmaco-resistant cases or instances in which levels of clinical acuity are so high that therapeutic lag times associated with most antidepressive treatments would be unacceptable. Reports of postpartum mECT were fewer in number (about 25% of included papers in our review) than those addressing prenatal mECT, as was also the case with prior reviews. As such, the conclusions from this literature review, which addresses mECT for mainly the acute treatment of depression in pregnancy and postnatal samples, are broadly consistent with those of prior reviews in terms of efficacy as an acute-phase treatment modality.

Although there appears to be a reasonably consistent signal for rapid, acute phase antidepressive efficacy in perinatally depressed patients, several questions remain. The first pertains to who the reviewed evidence applies to. Although most reviewed papers included clearly defined cases of perinatal unipolar or bipolar depression, many of the reviewed reports (a little more than 25% of the total) involved mixed cohorts of individuals, not all of whom received ECT, with clinical diagnoses outside of unipolar or bipolar major depression. This included all 9 observational studies and 12 case series, with diagnoses ranging from unspecified catatonic syndromes to unspecified postpartum psychoses and diagnosed primary psychotic illnesses. In studies with mixed samples, diagnosis-specific outcomes were often not provided—an important limitation considering that, although ECT has robust broad spectrum efficacy across most mood and psychotic disorders (112, 113), general and obstetric adverse event risks from mECT may be disproportionately increased in patients with primary psychotic disorders due to especially high rates of general medical comorbidity (114); substance use (115); pregnancy and delivery complications (116, 117); smoking, obesity, and other negative lifestyle factors (118, 119); and lower rates of preventive or obstetric care seeking/medical follow-up (120, 121). Additionally, an essential core treatment for patients with chronic psychoses, antipsychotic drugs, may increase the risk of gestational diabetes (122), an independent risk factor for numerous pregnancy and neonatal complications (123). While the inclusion of papers with mixed samples and incomplete or disparate approaches to the reporting of diagnosis-specific outcomes are clear limitations, their inclusion in the paper is consistent with our objective of conducting a scoping literature review.

Following an acute phase response to mECT, the continuation (extension) of antidepressive effects becomes most relevant, given the possibility of depressive symptom relapse once a course of ECT has ended. In a meta-analysis of 32 studies of non-puerperal depressive patients who responded to an acute course of ECT, estimated relapse rates were 37% at 6 months and 51% at one year (124), highlighting the need for continuation and maintenance ECT for a significant number of acutely treated patients. The estimated reduction in relapse risk with maintenance ECT in non-puerperal depressed patients may be substantial, even when compared to pharmacologically treated controls (RR for relapse, 0.8 to 0.5) (125); however, to our knowledge, no such estimates have been established for perinatally depressed patients. Very few reports included in this scoping review provided detailed information on the duration of acute responses and only 9 case reports (39, 46, 50, 73, 76, 79, 81, 86, 97) and one small case series (37) described the delivery of continuation of maintenance ECT. A smaller number of reports described early relapses of severe depressive symptoms after an initial acute ECT series (82, 88, 92, 101), some of which required additional ECT treatment sessions (54, 70, 97, 99). Although the case literature reviewed here and existing literature from non-puerperal samples of depressed patients raises the strong possibility of benefit, the effectiveness of continuation and maintenance mECT for perinatal depression remains understudied, constituting an important and persisting clinical knowledge gap.

For decades, ketamine has been used as an alternative anesthesia induction agent when ECT seizures become too short or too difficult to elicit when using first- and second-line agents such as methohexital, propofol, or etomidate (126). Ketamine is not an anesthetic of choice for mECT owing to the potential for increasing seizure duration and for enhanced hemodynamic responses with associated elevations in intracranial pressure (127). However, ketamine and its S-enantiomer, esketamine, have since become established, rapidly acting antidepressive agents in their own right (128), which has raised interest in the therapeutic potential of ketamine-augmented ECT. Only one of the reviewed reports described the clinical effects of ketamine-augmented ECT for an acutely suicidal depressed patient during pregnancy (78). In this case, remission occurred after 8 treatments. However, time course to remission was not described, and there was no viable means of determining if ketamine augmentation was required to achieve that outcome. As of this writing, the evidence base for non-puerperal depression does not show an advantage of prioritizing ketamine over other anesthesia induction agents for the purposes of maximizing therapeutic efficacy (129). Furthermore, ketamine is contraindicated in pregnancy given a lack of sufficient reproductive and safety data (130), as well as potential associations of ketamine exposure with neurotoxicity during fetal development in preclinical studies and adverse neurodevelopmental outcomes in neonates with repeated exposure to ketamine anesthesia (131–133). The drug label for esketamine indicates that it is also not recommended during pregnancy owing to insufficient reproductive and neonatal safety data (134). As such, augmentation with ketamine or esketamine for the specific purpose of enhancing the efficacy of mECT or accelerating the time to remission with mECT in depressed pregnant patients cannot be recommended at this time.

Interestingly, eight of the reviewed reports described the indication for ECT as the preferred treatment for the specific context of pregnancy, motivated in some cases by the desire to minimize potentially teratogenic medication exposures and other adverse effects (34, 37, 39, 52, 54–56, 66, 70). However, the safety of ECT in pregnant patients has been the subject of debate. In prior reviews and for most cases in this review, ECT appeared to be relatively well tolerated. Frequently reported maternal and fetal adverse events from both prior reviews and ours included transient fetal arrhythmias/fetal bradycardia, uterine contractions, abdominal or pelvic pain, premature deliveries, placental abruption, threatened abortion, and vaginal bleeding. In most cases where adverse events were reported, a healthy term delivery was the ultimate outcome. On the other hand, only one observational study investigated a limited range of neonatal adverse events associated with ECT during 97 pregnancies (24), reporting 2 cases of stillbirth, 14 cases of premature delivery, and 9 cases of 5-minute Apgar scores <7 (with none in the range of 0-3). Individual case reports and case series also documented a variety of adverse fetal/neonatal outcomes including 2 cases of fetal death/stillbirth (33, 42) and one case of miscarriage (48); 7 cases of premature delivery between 31 and 35^+4^ weeks gestation due to pregnancy induced hypertension (57), preterm labor (65, 69), preterm premature rupture of membranes (72), fetal growth restriction (100), and recurrence of depressive symptoms (101); 6 reports of congenital malformations (37, 41, 69, 84, 97, 101), and one report of confirmed placental abruption (94). However, none of these reports can be considered comprehensive in terms of adverse event surveillance. And in the absence of valid denominator data, adverse events from individual case reports (e.g., congenital malformations) cannot be used to estimate event rates for comparisons against background rates [e.g., 3% background congenital malformations rate (135), even if all reported cases were true cases.

Prior reviews of safety data for ECT during pregnancy have documented similar adverse events in ECT-treated pregnancies but differed in their main conclusions. Four reviews supported the safety of ECT during pregnancy (12, 15, 17, 110, 111). However, two reviews suggested that ECT should be considered only when other treatment options are ineffective or infeasible based on low data quality (16) or high reported frequencies of adverse events in general (29%) and child fatalities in particular (7.1%) (14). The review by Leiknes and colleagues (14) has been criticized for including studies dating back to the early 1940s, decades before modified ECT was standard-of-care practice, and for counting adverse events that were not likely to be related to ECT (19, 136). In our review, only a limited number of papers included sufficient details on confounding factors such as maternal psychiatric and general medical comorbidities, pre-ECT obstetric complications, and medications taken prior to or during index courses of mECT, making it difficult in many instances to draw firm causal links between ECT and reported adverse events (137).

The ethical considerations pertaining to the inclusion of pregnant patients in clinical therapeutics research continue to be debated (138). Therefore, it is highly unlikely that clinical decisions pertaining to the effectiveness and safety of mECT for perinatal depression will be guided by evidence from randomized trials any time soon, especially when taking into account the added complexities of addressing issues of capacity and valid informed consent for research participation in highly-vulnerable patients who may require mECT for severe and often psychotic or catatonic illnesses (139). For the time being, we anticipate increasing use of data from linked national registers, such as the Swedish National Quality Registry for ECT, which includes information on patient diagnoses, symptom severities, ECT indications and treatment characteristics, treatment course, side-effects, concomitant medications, and other data elements (140). Linkages with other registries, including birth registries, enabled the construction of retrospective cohorts that include perinatally depressed patients in two of the larger-scale studies included in this review (24, 30). Similarly, we anticipate that electronic medical records-linkage systems will become increasingly important data sources for future studies of mECT for perinatal depression, especially with the continued development of integrated networks between multiple healthcare institutions and linkages with additional sources of structured and unstructured data, such as vital records and clinical symptom measures (141).

There are several limitations for this review in addition to those outlined above. First, the results of this review are current only through 12/31/2024, when the literature search was conducted. Second, a specific definition of the perinatal period or perinatal onset was seldom provided in the reviewed reports, particularly for the postpartum. Third, even though treatment resistance was a specific indication for mECT in several reports, detailed information was often not available regarding the quality of therapeutic trials preceding ECT, including information on previously used therapeutic interventions, their respective doses or frequencies of use, previous treatment durations, or estimated levels of adherence. When information was provided about pre-ECT treatments, it was often unclear if they were utilized during the current episode of depression, making it difficult to define the levels of therapeutic resistance ECT was being administered for. Fourth, even a relatively brief acute series mECT involves a complex set of interventions, co-interventions, and procedures that may each act as confounders or effect modifiers for questions related to its overall effectiveness or safety for perinatal depression. Regarding confounding, it was not possible to draw clear conclusions regarding causal links between ECT and several of the reported adverse events, as discussed earlier. Concerning effect modification, it was not possible to assess therapeutic or safety outcomes between subgroups defined by concomitant psychotropic or other types of medications, anesthetic agents, obstetric history, primary psychiatric diagnosis, psychiatric or general medical comorbidity, or ECT electrode placement, although controlled evidence has shown generally comparable response rates for ECT with BT, RUL, and BF lead placements in people with non-puerperal unipolar or bipolar depression (142–144). And finally, post-ECT follow-up periods were generally brief across the reviewed reports; thus, longer-term outcomes were unknown for most individual reports.

## 5 Conclusions

The current report presents an updated scoping review of the literature describing the broad effectiveness and key safety issues relevant to mECT for perinatal depression. Although registry studies have contributed meaningfully to our understanding of mECT effectiveness and safety in recent years, over 89% of the reviewed literature was still comprised of case reports and case series. No randomized trials were available for review. Although the maternal safety profile of mECT appears reassuring thus far, the available data are far from comprehensive. Fetal and neonatal safety risks are less-well-understood, and lactation information was often not included in reports of postpartum ECT. Therefore, many efficacy and safety questions remain, including for continuation or maintenance mECT and for important patient subgroups discussed in this review. Future reports of mECT for perinatal depression should focus on thoroughly describing treatment parameters, including frequencies of treatment sessions, initial doses and titration methods, and complete information on subsequent stimulation parameters (including changes), as has been recommended by others outside of the perinatal context (145).

Pragmatically, while mECT offers the prospect of rapid antidepressive benefit, patients with lower-acuity or less-impairing depression will not require–or even desire—a therapeutic procedure as intensive as mECT (146). For cases of perinatal depression with severe psychosis, catatonia (malignant and benzodiazepine-resistant catatonia, in particular), suicidality, other direct and serious threats to physical integrity (e.g., nutritional compromise, etc.), and marked treatment resistance, ECT is an indispensable therapeutic option that should not be withheld (11, 147). When these severe indications are absent, but ECT is still a therapeutic preference, the tilting of benefits over risks will usually be far less steep. In such cases, we recommend supportive, measured communication that considers the risks of untreated or undertreated depression, the possibility (though not a guarantee) of rapid benefit from mECT, a relatively reassuring maternal safety profile based on information to date, and potential fetal/neonatal risks–including from relatively rare but potentially severe complications such as prolonged seizures, prolonged uterine contractions, decreased uterine blood flow, and hypoxic damage—and precautions and strategies used to manage those risks. Given the high levels of acuity that are likely to be encountered in perinatally depressed patients being considered for mECT, the capacity for providing valid informed consent will require rigorous evaluation, including an assessment of patients’ ability to consider both their own needs and risks as well as those of the fetus or infant (148). Whenever possible, the assessment of ECT suitability and risk stratification should be initially conducted and periodically reviewed by a multidisciplinary team including psychiatry, obstetrics, anesthesiology, and pediatrics.

## Data Availability

The original contributions presented in the study are included in the article/**Supplementary Material**. Further inquiries can be directed to the corresponding author.

## References

[1] Gaynes BN Gavin N Meltzer-Brody S Lohr KN Swinson T Gartlehner G . Perinatal depression: Prevalence, screening accuracy, and screening outcomes. Evid Rep Technol Assess (Summ). (2005) 119:1–8. doi: 10.1037/e439372005-001, PMID: 15760246 PMC4780910

[2] Underwood L Waldie K D’Souza S Peterson ER Morton S . A review of longitudinal studies on antenatal and postnatal depression. Arch Womens Ment Health. (2016) . 19:711–20. doi: 10.1007/s00737-016-0629-1, PMID: 27085795

[3] Dagher RK Bruckheim HE Colpe LJ Edwards E White DB . Perinatal depression: challenges and opportunities. J Women’s Health. (2021) . 30:154–9. doi: 10.1089/jwh.2020.8862, PMID: 33156730 PMC7891219

[4] Grogoriadis S VanderPorten EH Mamisashvili L Tolminson G Dennis C-L Koren G . The impact of maternal depression during pregnancy on perinatal outcomes: a systematic review and meta-analysis. J Clin Psychiatry. (2013) . 74:e321–41. doi: 10.4088/JCP.12r07968, PMID: 23656857

[5] Lommerse K Knight M Nair M Deneux-Tharaux C van den Akker T . The impact of reclassifying suicides in pregnancy and in the postnatal period on maternal mortality ratios. Int J Obstet Gynaecol. (2019) . 126:1088–92. doi: 10.1111/1471-0528, PMID: 29528573

[6] Lommerse KM Merelle S Rietveld AL Berkelmans G van den Akker T . Netherlands Audit Committee Maternal Mortality and Morbidity. The contribution of suicide to maternal mortality: A nationwide population-based cohort study. BJOG. (2024) . 131:1392–8. doi: 10.1111/1471-0528.17784, PMID: 38344899

[7] Rodriguez-Cabezas L Clark C . Psychiatric emergencies in pregnancy and postpartum. Clin Obstet Gynecol. (2018) . 61:615–27. doi: 10.1097/GRF.0000000000000377, PMID: 29794819 PMC6143388

[8] Waqas A Nadeem M Rahman A . Exploring heterogeneity in perinatal depression: a comprehensive review. BMC Psychiatry. (2023) . 23:643. doi: 10.1186/s12888-023-05121-z, PMID: 37667216 PMC10478465

[9] Bobo WV . The diagnosis and management of bipolar I and II disorders: clinical practice update. Mayo Clin Proc. (2017) . 92:1532–51. doi: 10.1016/j.mayocp.2017.06.022, PMID: 28888714

[10] Kirov G Jauhar S Sienaert P Kellner CH McLoughlin DM . Electroconvulsive therapy for depression: 80 years of progress. Br J Psychiatry. (2021) . 219:594–7. doi: 10.1192/bjp.2021.37, PMID: 35048827

[11] Ward HB Fromson JA Cooper JJ De Oliveira G Almeida M . Recommendations for the use of ECT in pregnancy: literature review and proposed clinical protocol. Arch Women’s Ment Health. (2018) . 21:715–22. doi: 10.1007/s00737-018-0851-0, PMID: 29796968

[12] Cipolla S Catapano P Messina M Pezzella P Giordano GM . Safety of electroconvulsive therapy (ECT) in pregnancy: a systematic review of case reports and case series. Arch Women’s Ment Health. (2024) . 27:157–78. doi: 10.1007/s00737-023-01394-1, PMID: 37957411 PMC10933171

[13] Gressier F Rotenberg S Cazas O Hardy P . Postpartum electroconvulsive therapy: a systematic review and case report. Gen Hosp Psychiatry. (2015) . 37:310–4. doi: 10.1016/j.genhosppsych.2015.04.009, PMID: 25929986

[14] Leiknes KA Cooke MJ Jarosch-von Schweder L Harboe I Hoie B . Electroconvulsive therapy during pregnancy: a systematic review of case studies. Arch Women’s Ment Health. (2015) . 18:1–39. doi: 10.1007/s00737-013-0389-0, PMID: 24271084 PMC4305619

[15] Miller LJ . Use of electroconvulsive therapy during pregnancy. Hosp Comm Psychiatry. (1994) . 45:444–50. doi: 10.1176/ps.45.5.444, PMID: 8045538

[16] Pacheco F Guiomar R Brunoni AR Buhagiar R Evagorou O Roca-LOecumberri A . Efficacy of non-invasive brain stimulation in decreasing depression symptoms during the peripartum period: a systematic review. J Psychiatr Res. (2021) . 140:443–60. doi: 10.1016/j.jpsychires.2021.06.005, PMID: 34147932

[17] Pompili M Dominici G Giordano G Longo L Serafini G Lester D . Electroconvulsive treatment during pregnancy: a systematic review. Expert Rev Neurother. (2014) . 14:1377–90. doi: 10.1586/14737175.2014.972373, PMID: 25346216

[18] Saatcioglu O Tomruk NB . The use of electroconvulsive therapy in pregnancy: a review. Isr J Psychiatry Relat Sci. (2011) . 48:6–11. 21572236

[19] Sinha P Goyal P Andrade C . A meta-review of the safety of electroconvulsive therapy in pregnancy. J ECT. (2017) . 33:81–8. doi: 10.1097/YCT.0000000000000362, PMID: 28009621

[20] Spodniaková B Halmo M Nosáľová P . Electroconvulsive therapy in pregnancy—a review. J Obstet Gynaecol. (2015) . 35:659–62. doi: 10.3109/01443615.2014.990427, PMID: 25526509

[21] Tricco AC Lillie E Zarin W O’Brien KK Colquhoun H Levac D . PRISMA extension for scoping reviews (PRISMA-ScR): checklist and explanation. Ann Intern Med. (2018) . 169:467–73. doi: 10.7326/M18-0850, PMID: 30178033

[22] Andrade C . Active placebo, the parachute meta-analysis, the Nobel Prize, and the efficacy of electroconvulsive therapy. J Clin Psychiatry. (2021) 82:21f13992. doi: 10.4088/JCP.21f13992, PMID: 34000105

[23] Rasmussen KG . Sham electroconvulsive therapy studies in depressive illness: a review of the literature and consideration of the placebo phenomenon in electroconvulsive therapy practice. J ECT. (2009) 25:54–9. doi: 10.1097/YCT.0b013e3181719b23, PMID: 18580816

[24] Arnison T Rask O Nordenskjöld A Movahed Rad P . Safety of and response to electroconvulsive therapy during pregnancy: Results from population-based nationwide registries. Acta Psychiatr Scand. (2024) . 150:360–71. doi: 10.1111/acps.13623, PMID: 37852926

[25] Babu GN Thippeswamy H Chandra PS . Use of electroconvulsive therapy (ECT) in postpartum psychosis–a naturalistic prospective study. Arch Womens Ment Health. (2013) . 16:247–51. doi: 10.1007/s00737-013-0342-2, PMID: 23568390

[26] Hauge C Rohde C Østergaard SD . Treatment of postpartum psychotic- or mood disorder requiring admission: A nationwide study from Denmark. Acta Psychiatr Scand. (2024) .150:395–403. doi: 10.1111/acps.13585, PMID: 37339779

[27] Haxton C Kelly S Young D Cantwell R . The efficacy of electroconvulsive therapy in a perinatal population: A comparative pilot study. J ECT. (2016) . 32:113–5. doi: 10.1097/YCT.0000000000000278, PMID: 26479488

[28] Raghuraman BS Varshney P Sinha HTP Ganjekar S Desai G Chandra PS . Electroconvulsive therapy (ECT) for severe mental illness (SMI) during perinatal period: The role of bifrontal (BF) ECT. Brain Stimul. (2019) .12:388. doi: 10.1016/j.brs.2018.12.242

[29] Reed P Sermin N Appleby L Faragher B . A comparison of clinical response to electroconvulsive therapy in puerperal and non-puerperal psychoses. J Affect Disord. (1999) . 54:255–60. doi: 10.1016/s0165-0327(99)00012-9, PMID: 10467968

[30] Ronnqvist I Brus O Hammar A Landen M Lundberg J Nordanskog P . Rehospitalization of postpartum depression and psychosis after electroconvulsive therapy: A population-based study with a matched control group. J ECT. (2019) . 35:264–71. doi: 10.1097/YCT.0000000000000578, PMID: 31764450 PMC6903363

[31] Rundgren S Brus O Bave U Landen M Lundberg J Nordanskog P . Improvement of postpartum depression and psychosis after electroconvulsive therapy: A population-based study with a matched comparison group. J Affect Disord. (2018) 235:258–64. doi: 10.1016/j.jad.2018.04.043, PMID: 29660641

[32] Saluja S Cooter A Roberts S Branjerdporn G . Pharmacotherapy and electroconvulsive therapy prescription for women with depressive and anxiety disorders in a psychiatric mother-baby unit. Australas Psychiatry. (2024) . 32:573–81. doi: 10.1177/10398562241278856, PMID: 39209800

[33] Baki ED Akici OC Guzel HI Kokulu S Ela Y Sivaci RG . Our anesthesia experience during electroconvulsive therapy in pregnant patients. Braz J Anesth. (2016) . 66:555. doi: 10.1016/j.bjane.2014.07.001, PMID: 27591476

[34] Balki M Castro C Ananthanarayan C . Status epilepticus after electroconvulsive therapy in a pregnant patient. Int J Obstet Anesth. (2006) . 15:325–8. doi: 10.1016/j.ijoa.2006.01.005, PMID: 16774832

[35] Ballone NT . Clinical considerations for electroconvulsive therapy in a breastfeeding mother for postpartum depression without psychosis. J ECT. (2023) 39:e17.

[36] Bergink V Koorengevel KM . Treatment of postpartum depression with psychotic features. Arch Womens Ment Health. (2011) 14:S60.

[37] Bulut M Bez Y Kaya MC Copoglu US Bulbul F Savas HA . Electroconvulsive therapy for mood disorders in pregnancy. J ECT. (2013) . 29:e19–20. doi: 10.1097/YCT.0b013e318277cce2, PMID: 23519218

[38] Bhatia SC Baldwin SA Bhatia SK . Electroconvulsive therapy during the third trimester of pregnancy. J ECT. (1999) 15:270–4., PMID: 10614034

[39] Bozkurt A Karlidere T Isintas M Ozmenler NK Ozsahin A Yanarates O . Acute and maintenance electroconvulsive therapy for treatment of psychotic depression in a pregnant patient. J ECT. (2007) . 23:185–7. doi: 10.1097/YCT.0b013e31806db4dd, PMID: 17804997

[40] Brown NI Mack PF Mitera DM Dhar P . Use of the ProSeal laryngeal mask airway in a pregnant patient with a difficult airway during electroconvulsive therapy. Br J Anaesth. (2003) . 91:752–4. doi: 10.1093/bja/aeg227, PMID: 14570805

[41] Bulbul F Copoglu US Alpak G Unal A Demir B Tastan MF . Electroconvulsive therapy in pregnant patients. Gen Hosp Psychiatry. (2013) . 35:636–9. doi: 10.1016/j.genhosppsych.2013.06.008, PMID: 23890597

[42] Bulbul F Copoglu US Demir B Bulut M Alpak G Unal A . Sociodemographic characteristics and clinical follow-up results of pregnant patients hospitalized for psychiatric disorders. Dusunen Adam J Psychiatry Neurol Sci. (2014) . 27:21–6. doi: 10.5350/DAJPN2014270103

[43] Chase T Shah A Maines J Fusick A . Psychotic pregnancy denial: a review of the literature and its clinical considerations. J Psychosom Obstet Gynecol. (2012) 42:253–7. doi: 10.1080/0167482X.2020.1789584, PMID: 32729360

[44] Choi B-S Kim J-M Lee H-Y . A young woman who suffered a fractured vertebra during electroconvulsive therapy. Psychiatr Ann. (2018) 48:532–5. doi: 10.3928/00485713-20181010-01

[45] Cutajar P Wilson D Mukherjee T . ECT used in depression following childbirth, in a woman with learning disabilities. Br J Learn Disabil. (1998) 26:115–7. doi: 10.1111/j.1468-3156.1998.tb00062.x

[46] De Asis SJ Helgeson L Ostroff R . The use of propofol to prevent fetal deceleration during electroconvulsive therapy treatment. J ECT. (2013) . 29:e57–8. doi: 10.1097/YCT.0b013e318290f9e7, PMID: 23609517

[47] DeBattista C Cochran M Barry JJ Brock-Utne JG . Fetal heart rate decelerations during ECT-induced seizures: is it important?. Acta Anaesthesiol Scand. (2003) 47:101–3. doi: 10.1034/j.1399-6576.2003.470119.x, PMID: 12492807

[48] Echevarría Moreno M Martin Muñoz J Sanchez Valderrabanos J Vázquez Gutierrez T . Electroconvulsive therapy in the first trimester of pregnancy. J ECT. (1998) . 14:251–4. doi: 10.1097/00124509-199812000-00006, PMID: 9871846

[49] Erturk A Aktoz F Orgul G Mutlu E Demir B Tuncer ZS . Administration of electroconvulsive therapy for major depression during pregnancy: a case report. J Obstet Gynaecol. (2020) . 40:277–8. doi: 10.1080/01443615.2019.1628727, PMID: 31368383

[50] Forray A Ostroff RB . The use of electroconvulsive therapy in postpartum affective disorders. J ECT. (2007) . 23:188–93. doi: 10.1097/yct.0b013e318074e4b1, PMID: 17804998

[51] Gahr M Blacha C Connemann BJ Freudenmann RW Schönfeldt-Lecuona C . Successful treatment of major depression with electroconvulsive therapy in a pregnant patient with previous non-response to prefrontal rTMS. Pharmacopsychiatry. (2012) . 45:79–80. doi: 10.1055/s-0031-1297936, PMID: 22174028

[52] Gannon JM Gopalan P Solai LK Lim G Phillips JM Beck S . ECT for a pregnant patient with bipolar disorder in the COVID-19 Era: A clinical conundrum. Bipolar Disord. (2021) . 23:524–7. doi: 10.1111/bdi.13061, PMID: 33590703 PMC8013649

[53] Gonzales N Quinn DK Rayburn W . Perinatal catatonia: a case report and literature review. Psychosomatics. (2014) 55:708–14. doi: 10.1016/j.psym.2014.01.009, PMID: 25262045

[54] Griffiths EJ Lorenz RP Baxter S Talon NS . Acute neurohumoral response to electroconvulsive therapy during pregnancy. A Case Rep J Reprod Med. (1989) . 34:907–11., PMID: 2573726

[55] Grover S Sahoo S Chakrabarti S Basu D Singh SM Avasthi A . ECT in the postpartum period: A retrospective case series from a tertiary health care center in India. Indian J Psychol Med. (2018) . 40:562–7. doi: 10.4103/IJPSYM.IJPSYM_105_18, PMID: 30533953 PMC6241200

[56] Grover S Sharma P Chakrabarti S . Use of electroconvulsive therapy during postpartum: A retrospective chart review. Indian J Psychiatry. (2024) . 66:572–5. doi: 10.4103/Indianjpsychiatry.Indianjpsychiatry_165_24, PMID: 39100373 PMC11293785

[57] Grover S Sikka P Saini SS Shni N Chakrabarti S Dua D . Use of modified bilateral electroconvulsive therapy during pregnancy: A case series. Indian J Psychiatry. (2017) . 59:487–92. doi: 10.4103/psychiatry.IndianJPsychiatry_50_17, PMID: 29497193 PMC5806330

[58] Guillet C Didi Roy R Hussami A Girod JC . Electroconvulsive therapy and dopa-responsive dystonia: Improvements in neurological symptoms after electroconvulsive therapy treatment. J ECT. (2020) 36:E53–4. doi: 10.1097/YCT.0000000000000696, PMID: 32453189

[59] Gunduz T Yucel E Tan D Gercek A Ilter E Celik A Haliloglu B Ozekici U . Induction of preterm uterine contractions with electroconvulsive therapy in a 32 week pregnant woman: A case report. Turk Jinekoloji ve Obstetrik Dernegi Dergisi. (2010) 7:96.

[60] Herzog A Detre T . Postpartum psychoses. Dis Nerv Syst. (1974) 35:556–9. 17896755

[61] Howe GB Srinivasan M . A case study on the successful management of Cotard's syndrome in pregnancy. Int J Psychiatry Clin Pract. (1999) 3:293–5. doi: 10.3109/13651509909068399, PMID: 24921235

[62] Isik M Esin G . Temporomandibular dislocation secondary to modified electroconvulsive therapy. Anadolu Psikiyatri Derg. (2019) 20:336.

[63] Iwasaki K Sakamoto A Hoshino T Ogawa R . Electroconvulsive therapy with thiamylal or propofol during pregnancy. Can J Anesth. (2002) 49:324–5. doi: 10.1007/BF03020541, PMID: 11861360

[64] Reveles Jensen KH Pedersen ST Vinther Hansen M Jørgensen MB . Shocking colours - ECT temporarily improves colour perception in a colour-blind patient. Brain Stimul. (2020) 13:957–8. doi: 10.1016/j.brs.2020.04.018, PMID: 32360390

[65] Kasar M Saatcioglu O Kutlar T . Electroconvulsive therapy use in pregnancy. J ECT. (2007) 23:183–4. doi: 10.1097/yct.0b013e318065b12f, PMID: 17804996

[66] Kisa C Yildirim SG Aydemir C Cebeci S Goka E . Prolonged electroconvulsive therapy seizure in a patient taking ciprofloxacin. J ECT. (2005) 21:43–4. doi: 10.1097/00124509-200503000-00012, PMID: 15791178

[67] Leite D Antunes AF . Postpartum depression, catatonia and COVID-19 infection: One case, different clinical presentations. Eur Psychiatry. (2022) 65:S566. doi: 10.1192/j.eurpsy.2022.1450

[68] Levy Y Austin M-P Halliday G . Use of ultra-brief pulse electroconvulsive therapy to treat severe postnatal mood disorder. Australas Psychiatry. (2012) . 20:429–32. doi: 10.1177/1039856212458979, PMID: 23014119

[69] Livingston J Johnstone W Hadi H . Electroconvulsive therapy in a twin pregnancy: A case report. Am J Perinatol. (1994) .11:116–8. doi: 10.1055/s-2007-994569, PMID: 8198651

[70] Maletzky BM . The first-line use of electroconvulsive therapy in major affective disorders. J ECT. (2004) . 20:112–7. doi: 10.1097/00124509-200406000-00007, PMID: 15167428

[71] Malhotra N Vani Malhotra P Bhardwaj R . Modified electroconvulsive therapy during pregnancy. J Anaesth Clin Pharmacol. (2008) 24:351–2.

[72] Martinez-Sosa N Delaney J McLeod-Bryant S . A challenging case of catatonia during pregnancy. Pers Med Psychiatry. (2020) 23-24:100064. doi: 10.1016/j.pmip.2020.100064

[73] May MH Reynolds-May MF . Postpartum depression treated in private practice. In Taylor CB (Ed.), How to Practice Evidence-Based Psychiatry: Basic Principles and Case Studies. (2010), 287–305. Washington, DC: American Psychiatric Publishing, Inc.

[74] Morris B . Depression: the midwife who wanted to die. Nurs Mirror. (1979) 149:20–1., PMID: 260145

[75] Mynors-Wallis LM . Caution about sorcery. Br J Psychiatry. (1989) 155:570., PMID: 2635901

[76] O’Reardon JP Cristancho MA von Andreae CV Cristancho P Weiss D . Acute and maintenance electroconvulsive therapy for treatment of severe major depression during the second and third trimesters of pregnancy with infant follow-up to 18 months. J ECT. (2011) 27:e2–e26. doi: 10.1097/yct.0b013e3181e63160, PMID: 20562638

[77] Ozgul U Erdogan MA Sanli M Erdil F Begec Z Durmus M . Anaesthetic management in electroconvulsive therapy during early pregnancy. Turkish J Anesth Reanimation. (2014) . 42:145–7. doi: 10.5152/tjar.2014.73645, PMID: 27366409 PMC4894223

[78] Patel A Saucier AC Hobday C Chacko R . Safety and efficacy of ketamine-augmented electroconvulsive therapy in third trimester pregnancy complicated by covid-19. Baylor Univ Med Center Proc. (2022) . 35:874–5. doi: 10.1080/08998280.2022.2106415, PMID: 36304628 PMC9586677

[79] Pesiridou A Baquero G Cristancho P Wakil L Altinay M Kim D . A case of delayed onset of threatened premature labor in association with electroconvulsive therapy in the third trimester of pregnancy. J ECT. (2010) . 26:228–30. doi: 10.1097/yct.0b013e3181c3aef3, PMID: 20375702

[80] Pierre D Pericaud A Guerby P Castel A Schmitt L Yrondi A . Bitemporal electroconvulsive therapy during the second trimester of pregnancy in bipolar disroders: a case report. J ECT. (2020) 36:E14–5. doi: 10.1097/YCT.0000000000000634, PMID: 31913929

[81] Pinette MG Santarpio C Wax JR Blackstone J . Electroconvulsive therapy in pregnancy. Obstet Gynecol. (2007) . 110:465–6. doi: 10.1097/01.aog.0000265588.79929.98, PMID: 17666629

[82] Rabie N Shah R Ray-Griffith S Coker JL Magann EF Stowe ZN . Continuous fetal monitoring during electroconvulsive therapy: A prospective observation study. Int J Women’s Health. (2021) . 13:1–7. doi: 10.2147/ijwh.s290934, PMID: 33442300 PMC7797309

[83] Ratan DA Friedman T . Capgras syndrome in postpartum depression. Ir J Psychol Med. (1997) 14:117–8.

[84] Ray-Griffith SL Coker JL Rabie N Eads LA Golden KJ Stowe ZN . Pregnancy and electroconvulsive therapy. J ECT. (2016) . 32:104–12. doi: 10.1097/yct.0000000000000297, PMID: 26796501 PMC4877273

[85] Repke JT Berger NG . Electroconvulsive therapy in pregnancy. Obstet Gynecol. (1984) 63:39S–41S., PMID: 6700879

[86] Richardson AL Russai R Queenan K Murtagh J Whelan M Lucas DN . Electroconvulsive therapy for symptomatic bipolar disorder in the third trimester of pregnancy. Int J Obstetric Anesth. (2018) S61:s60.

[87] Rineh HM Khoshrang H Alavi CE Rimaz S Biazar G Rad RS Sani MK . Anesthesia management of electroconvulsive therapy at the late of pregnancy: A case report. Int J Womens Health Reprod Sci. (2020) 8:239–42. doi: 10.15296/ijwhr.2020.39

[88] Sahan E Zengin-Eroglu M . Negativism associated urinary bladder overdistension: A case report. Dusunen Adam: J Psychiatry Neurol Sci. (2017) 30:262–5. doi: 10.5350/dajpn2017300311

[89] Salzbrenner S Breeden A Jarvis S Rodriguez W . A 48-year-old woman primigravid via *in vitro* fertilization with severe bipolar depression and preeclampsia treated successfully with electroconvulsive therapy. J ECT. (2011) 27:e1–e3. doi: 10.1097/yct.0b013e3181ca4d22, PMID: 21343708

[90] Sandal G Cetin H . Electroconvulsive therapy during pregnancy as a possible cause of mobius syndrome: Additional clinical observation. Genet Couns. (2014) 25:357–61., PMID: 25804012

[91] Sarma S Quinn E Branjerdporn G . Safe delivery of electroconvulsive therapy in postpartum depression patient with type 1 Chari malformation: a case study. J ECT. (2024) 40:e10–11. doi: 10.1097/YCT.0000000000000999, PMID: 38530929

[92] Serim B Ulaş H Özerdem A Alkın T . Electroconvulsive therapy in an adolescent pregnant patient. Progr Neuropsychopharmacol Biol Psychiatry. (2010) . 34:546–7. doi: 10.1016/j.pnpbp.2009.11.014, PMID: 19931585

[93] Shea AK Wolfman W . The role of hormone therapy in the management of severe postpartum depression in patients with Turner syndrome. Menopause. (2017) 24:1309–12. doi: 10.1097/GME.0000000000000915, PMID: 28609392

[94] Sherer DM D’Amico ML Warshal DP Stern RA Grunert HF Abramowicz JS . Recurrent mild abruptio placentae occurring immediately after repeated electroconvulsive therapy in pregnancy. Am J Obstet Gynecol. (1991) . 65:652–3. doi: 10.1016/0002-9378(91)90302-8, PMID: 1892192

[95] Strain AK Meltzer-Brody S Bullard E Gaynes BN . Postpartum catatonia treated with electroconvulsive therapy: A case report. Gen Hosp Psychiatry. (2012) 34:436.e3–436.e4. doi: 10.1016/j.genhosppsych.2011.11.010, PMID: 22227030

[96] Takubo Y Nemoto T Obata Y Baba Y Yamaguchi T Katagiri N . Effectiveness of kangaroo care for a patient with postpartum depression and comorbid mother-infant bonding disorder. Case Rep Psychiatry. (2019) 2019:9157214. doi: 10.1155/2019/9157214, PMID: 30937206 PMC6413388

[97] Walker R . ECT and twin pregnancy. Convulsive Ther. (1992) 8:131–6., PMID: 11941159

[98] Watanabe A Ayani N Waratani M Hasegawa T Ishii M Matsuoka T . A case of fetal tachycardia after electroconvulsive therapy a possible effect of maternal hypoxia and uterine contractions. Case Rep Psychiatry. (2019) 2019:3709612. doi: 10.1155/2019/3709612, PMID: 31355037 PMC6637665

[99] Wise MG Ward SC Townsend Parchman W . Case report of ECT during high-risk pregnancy. Am J Psychiatry. (1984) .141:99–10. doi: 10.1176/ajp.141.1.99, PMID: 6691474

[100] Yamada T Sawa R Abe H Kikuti F Mine K Ishikawa G . Serious bipolar depression amalgamation pregnancy with severe IUGR would be caused by placenta thrombus formation after ECT repeating itself. Placenta. (2007) . 28:A4–4. doi: 10.1016/j.placenta.2007.08.001

[101] Yang HS Seo HJ Lee YK . Anesthetic care for electroconvulsive therapy during pregnancy. Korean J Anesthesiol. (2011) . 60:217–20. doi: 10.4097/kjae.2011.60.3.217, PMID: 21490826 PMC3071488

[102] Ying P Carlisle G Reubins G . Pseudocholinesterase deficiency in a pregnant woman receiving ECT resulting in prolonged intubation and possible fetal distress. J ECT. (2022) . 38:e46.

[103] Dorn JB . Electroconvulsive therapy with fetal monitoring in a bipolar pregnant patient. Convuls Ther. (1985) 1:217–21., PMID: 11940826

[104] Montgomery SA Asberg M . A new depression scale designed to be sensitivie to change. Br J Psychiatry. (1979) . 134:382–9. doi: 10.1192/bjp.134.4.382, PMID: 444788

[105] Bush G Fink M Petrides G Dowling F Francis A . Catatonia. I. Rating scale and standardized examination. Acta Psychiatr Scand. (1996) . 93:129–36. doi: 10.1111/j.1600-0447.1996.tb09814.x, PMID: 8686483

[106] Rush AJ Trivedi MH Ibrahim HM Carmody TJ Arnow B Klein DN . The 16-item Quick Inventory of Depressive Symptomatology (QIDS), clinician rating (QIDS-C), and self-report (QIDS-SR): a psychometric evaluation in patients with chronic major depression. Biol Psychiatry. (2003) . 54:573–83. doi: 10.1016/s0006-3223(02)01866-8, PMID: 12946886

[107] Kroenke K Spitzer RL Williams JBW . The PHQ-9: validity of a brief depression severity measure. J Gen Intern Med. (2001) . 16:606–13. doi: 10.1046/j.1525-1497.2001.016009606.x, PMID: 11556941 PMC1495268

[108] Coman A Bondevik H . The ethical imperative of trauma-sensitive care for electroconvulsive therapy (ECT): Recipients’ experiences with care. J Ment Health. (2024) 33:177–84. doi: 10.1080/09638237.2023.2210650, PMID: 37218175

[109] Asberg M Montgomery SA Perris C Schalling D Sedvall G . A comprehensive psychopathological rating scale. Acta Psychiatr Scand. (1978) . 57:5–27. doi: 10.1111/j.1600-0447.1978.tb02357.x, PMID: 277059

[110] Anderson EL Reti IM . ECT in pregnancy: a review of the literature from 1941 to 2007. Psychosom Med. (2009) . 71:235–42. doi: 10.1097/PSY.0b013e318190d7ca, PMID: 19073751

[111] Calaway K Coshal S Jones K Coverdale J Livingston R . A systematic review of the safety of electroconvulsive therapy use during the first trimester of pregnancy. J ECT. (2016) 32:230–5. doi: 10.1097/YCT.0000000000000330, PMID: 27327556

[112] Sinclair DJM Zhao S Qi F Yakyoma K Kwong JSW Adams DE . Electroconvulsive therapy for treatment-resistant schizophrenia. Schizophr Bull. (2019) . 45:730–2. doi: 10.1093/schbul/sbz037, PMID: 31150556 PMC6581135

[113] UK ECT Review Group . Efficacy and safety of electroconvulsive therapy in depressive disorders: a systematic review and meta-analysis. Lancet. (2003) . 361:799–808. doi: 10.1016/S0140-6736(03)12705-5, PMID: 12642045

[114] Jones DR Macias C Barreira PJ Fisher WH Hargreaves WA Harding CM . Prevalence, severity, and co-occurrence of chronic physical health problems of persons with serious mental illness. Psychiatr Serv. (2004) . 55:1250–7. doi: 10.1176/appi.ps.55.11.1250, PMID: 15534013 PMC2759895

[115] Nesvag R Knudsen GP Bakken IJ Hoye A Ystrom E Suren P . Substance use disorders in schizophrenia, bipolar disorder, and depressive illness: a registry-based study. Soc Psychiatry Psychiatr Epidemiol. (2015) . 50:1267–76. doi: 10.1007/s00127-015-1025-2, PMID: 25680837

[116] Fabre C Pauly V Baumstarck K Etchecopar-Etchart D Orleans V Llorca P-M . Pregnancy, delivery and neonatal complications in women with schizophrenia: a national population-based cohort study. Lancet Regional Health Europe. (2021) . 10:100209. doi: 10.1016/j.lanepe.2021.100209, PMID: 34806069 PMC8589714

[117] Judd F Komiti A Sheehan P Newman L Castle D Everall I . Adverse obstetric and neonatal outcomes in women with severe mental illness: To what extent can they be prevented? Schizophr Res. (2014) . 157:305–9. doi: 10.1016/j.schres.2014.05.030, PMID: 24934903

[118] Brown S Birtwistle J Roe L Thompson C . The unhealthy lifestyle of people with schizophrenia. Psychol Med. (1999) . 29:697–701. doi: 10.1017/s0033291798008186, PMID: 10405091

[119] Brown S Inskip H Barraclough B . Causes of the excess mortality of schizophrenia. Br J Psychiatry. (2000) . 177:212–7. doi: 10.1192/bjp.177.3.212, PMID: 11040880

[120] Fleischhacker WW Cetkovich-Bakmas M De Hert M Hennekens CH Lambert M Leucht S . Comorbid somatic illnesses in patients with severe mental disorders: clinical, policy, and research challenges. J Clin Psychiatry. (2008) . 69:514–9. doi: 10.4088/jcp.v69n0401, PMID: 18370570

[121] Seeman MV . Clinical interventions for women with schizophrenia: pregnancy. Acta Psychiatr Scand. (2012) . 127:12–22. doi: 10.1111/j.1600-0447.2012.01897.x, PMID: 22715925

[122] Betcher HK Montiel C Clark CT . Use of antipsychotic drugs during pregnancy. Curr Treat Options Psych. (2019) . 6:17–31. doi: 10.1007/s40501-019-0165-5, PMID: 32775146 PMC7410162

[123] Ye W Luo C Huang J Li C Liu Z Liu F . Gestational diabetes mellitus and adverse pregnancy outcomes: systematic review and meta-analysis. BMJ. (2022) . 377:e067946. doi: 10.1136/bmj-2021-067946, PMID: 35613728 PMC9131781

[124] Jelovac A Kolshus E McLoughlin DM . Relapse following successful electroconvulsive therapy for major depression: a meta-analysis. Neuropsychopharmacol. (2013) . 38:2467–74. doi: 10.1038/npp.2013.149, PMID: 23774532 PMC3799066

[125] Rowland T Mann R Azeem S . The efficacy and tolerability of continuation and maintenance electroconvulsive therapy for depression: a systematic review of randomized and observational studies. J ECT. (2023) . 39:141–50. doi: 10.1097/YCT.0000000000000914, PMID: 36961277

[126] Kellner CH Iosifescu DV . Ketamine and ECT: better alone than together? Lancet Psychiatry. (2017) . 4:348–9. doi: 10.1016/S2215-0366(17)30099-8, PMID: 28359863

[127] Ding Z White PF . Anesthesia for electroconvulsive therapy. Anesth Analgesia. (2002) . 94:1351–64. doi: 10.1097/00000539-200205000-00057, PMID: 11973219

[128] McIntyre RM Rosenblat JD Nemeroff CB Sanacora G Murrough JW Berk M . Synthesizing the evidence for ketamine and esketamine in treatment-resistant depression: an international expert opinion on the available evidence and implementation. Am J Psychiatry. (2021) . 178:383–99. doi: 10.1176/appi.ajp.2020.20081251, PMID: 33726522 PMC9635017

[129] McGirr A Berlim MT Bond DJ Chan PY Yatham LN Lam RW . Adjunctive ketamine in electroconvulsive therapy: Updated systematic review and meta-analysis. Br J Psychiatry. (2017) . 210:403–7. doi: 10.1192/bjp.bp.116.195826, PMID: 28385704

[130] Pacilio RM Lopez JF Parikh SV Patel PD Geller JA . Safe ketamine use in pregnancy: a nationwide survey and retrospective review of informed consent, counseling, and testing practices. J Clin Psychiatry. (2024) 85:24m15293. doi: 10.4088/JCP.24m15293, PMID: 39196890

[131] Dong C Rovnaghi CR Anand KJ . Ketamine exposure during embryogenesis inhibits cellular proliferation in rat fetal cortical neurogenic regions. Acta Anaesthesiol Scand. (2016) . 60:579–87. doi: 10.1111/aas.12689, PMID: 26822861 PMC4821784

[132] Yan J Li T-R Zhang Y Lu Y Jiang H . Repeated exposure to anesthetic ketamine can negatively impact neurodevelopment in infants: a prospective preliminary clinical study. J Child Neurol. (2014) . 29:1333–8. doi: 10.1177/0883073813517508, PMID: 24659739

[133] Zhao T Li Y Wei W Savage S Zhou L Ma D . Ketamine administered to pregnant rats in the second trimester causes long-lasting behavioral disorders in offspring. Neurobiol Dis. (2014) . 68:145–55. doi: 10.1016/j.nbd.2014.02.009, PMID: 24780497

[134] Spravato package label . (2024). Available online at: https://www.accessdata.fda.gov/drugsatfda_docs/label/2024/211243s015lbl.pdf (Accessed April 25, 2025).

[135] Centers for Disease Control and Prevention (CDC) . Update on overall prevalence of major birth defects--Atlanta, Georgia, 1978–2005. MMWR Morb Mortal Wkly Rep. (2008) 57:1–5. Available online at: https://www.cdc.gov/mmwr/preview/mmwrhtml/mm5701a2.htm (Accessed August 6, 2025)., PMID: 18185492

[136] Coshal S Jones K Coverdale J Livingston R . An overview of reviews on the safety of electroconvulsive therapy administered during pregnancy. J Psychiatr Prac. (2019) . 25:2–6. doi: 10.1097/PRA.0000000000000359, PMID: 30633726

[137] Joseph KS Mehrabadi A Lisonkova S . Confounding by indication and related concepts. Curr Epidemol Rep. (2014) . 1:1–8. doi: 10.1007/s40471-013-0004-y

[138] Rubin R . Addressing barriers to inclusion of pregnant women in clinical trials. JAMA. (2018) . 320:742–4. doi: 10.1001/jama.2018.9989, PMID: 30090925

[139] Biros M . Capacity, vulnerability, and informed consent for research. J Law Med Ethics. (2018) . 46:72–8. doi: 10.1177/1073110518766021, PMID: 29991882 PMC6035898

[140] Nordanskog P Hulten M Landen M Lundberg J von Knorring L Nordenskjold A . Electroconvulsive therapy in Sweden 2013: data from the National Quality Register for ECT. J ECT. (2015) . 31:263–7. doi: 10.1097/YCT.0000000000000243, PMID: 25973769 PMC4652632

[141] Casey JA Schwartz BS Stewart WF Adler NE . Using electronic health records for population health research: a review of methods and applications. Annu Rev Public Health. (2016) . 37:61–81. doi: 10.1146/annurev-publhealth-032315-021353, PMID: 26667605 PMC6724703

[142] Bailine SH Rifkin A Kayne E Selzer JA Vital-Herne J Blieka M . Comparison of bifrontal and bitemporal ECT for major depression. Am J Psychiatry. (2000) . 157:121–3. doi: 10.1176/ajp.157.1.121, PMID: 10618025

[143] Kellner CH Knapp R Husain MM Rasmussen K Sampson S Cullum M . Bifrontal, bitemporal and right unilateral electrode placement in ECT: randomised trial. Br J Psychiatry. (2010) . 196:226–34. doi: 10.1192/bjp.bp.109.066183, PMID: 20194546 PMC2830057

[144] Semkovska M Landau S Dunne R Kolshus E Kavanagh A Jelovac A . Bitemporal versus high-dose unilateral twice-weekly electroconvulsive therapy for depression (EFFECT-Dep): a pragmatic, randomized, non-inferiority trial. Am J Psychiatry. (2016) . 173:408–17. doi: 10.1176/appi.ajp.2015.15030372, PMID: 26892939

[145] Peterchev AV Rosa MA Deng Z-D Preudic J Lisanby SH . ECT stimulus parameters: rethinking dosage. J ECT. (2010) . 26:159–74. doi: 10.1097/YCT.0b013e3181e48165, PMID: 20805726 PMC2933093

[146] Rosenquist PB Dunn A Rapp S Gaba A McCall WV . What predicts patients’ expressed likelihood of choosing electroconvulsive therapy as a future treatment? J ECT. (2006) . 22:33–7. doi: 10.1097/00124509-200603000-00007, PMID: 16633204

[147] Xiao H Meng Y Liu S Cao Y Sun H Deng G . Non-invasive brain stimulation for treating catatonia: a systematic review. Front Psychiatry. (2023) . 14:1135583. doi: 10.3389/fpsyt.2023.1135583, PMID: 37260758 PMC10227525

[148] Rabheru K . The use of electroconvulsive therapy in special patient populations. Can J Psychiatry. (2001) . 46:710–9. doi: 10.1177/070674370104600803, PMID: 11692973

