# Supplementary material for: Modified electroconvulsive therapy for perinatal depression: scoping review
Source: Front Psychiatry. 2025 Aug 18;16:1619098. doi: 10.3389/fpsyt.2025.1619098 (PMC12400821; doi:10.3389/fpsyt.2025.1619098)
Supplement: Supplementary file 1 [file SupplementaryFile1.docx]

Supplemental Tables.

Suppl Table 1. Electronic databases used to identify relevant reports on the effects of electroconvulsive therapy (ECT) for perinatal depression*

| For published reports: | For unpublished/grey literature: |
| --- | --- |
| Ovid MEDLINE, 1946-present  Embase (Elsevier), 1947-current  Cochrane Database of Systematic Reviews (CDSR)  Cochrane Central Register of Controlled Trials (CENTRAL)  American Psychological Association PsychINFO (ProQuest), 1894-current  Web of Science (Science Citation Index Expanded [SCI-EXPANDED]), 1900-present  Web of Science (Social Sciences Citation Index [SCCI]), 1956-present  Web of Science Conference Proceedings Citation Index-Science [CPCI-S]), 1993-present  Web of Science Conference Proceedings Citation Index-Social Science & Humanities [CPCI-SSH]), 1993-present  Web of Science (BIOSIS Citation Index), 1969-present  CINAHL Ultimate (EBSCO), 1937-current | ClinicalTrials.gov  World Health Organization (WHO) International Clinical Trials Registry Platform (ICTRP)  Global Index Medicus |
| * All databases and registries were searched on 12/31/2024. | |

Suppl Table 2. Data elements abstracted from individual reports of modified electroconvulsive therapy (mECT) for perinatal depression

| Broad category: | Data element: | Definition/commentary: |
| --- | --- | --- |
| Study characteristics | Publication year | Year of publication |
|  | Authors | Last name of lead author |
|  | Study design/report type | RCT, prospective (non-randomized), retrospective cohort, case-control, case series, case report, other (specify) |
|  | Treatment setting | Specialized mother-baby unit, specialized mood disorder(s) unit, delivery suite, post-acute care unit, psychiatric hospital, general hospital, outpatient/ambulatory, other (specify), unspecified |
|  | Presence/absence of control group(s) | As specified in the individual report |
|  | Sample size | Total N, N by diagnosis |
|  | Number of ECT treatments | Total N, N by diagnosis |
|  | Duration of follow-up | As specified in the individual report |
| Enrollee details | Qualifying diagnos(es) | MDD, prenatal; MDD, postpartum; bipolar depression, prenatal; bipolar depression, postpartum; other unipolar depression, prenatal; other unipolar depression, postpartum; mood disorder diagnosis unspecified but clinical evidence of depression; other (specify) |
|  | Indication(s) for ECT | Treatment-resistant illness*, high suicide risk, psychotic symptoms/features, catatonia resistant to benzodiazepines, other catatonia, patient preference, other (specify), unspecified |
|  | * Criteria for treatment resistance | As specified in the individual report |
|  | mECT treatment phase | Acute depression, maintenance, both, other (specify), unspecified |
|  | Age | Mean or median age, exact age (case reports), or stratum specific proportions (if only age ranges reported), as specified in the individual report |
|  | Multiple gestation status | Singleton pregnancy, multiple gestation pregnancy, unspecified |
|  | Gestational weeks/trimester of pregnancy (or number of weeks postpartum) at mECT start | As specified in the individual report |
|  | Gestational weeks/trimester of pregnancy (or weeks post-partum) when mECT ended | As specified in the individual report |
|  | Pregnancy complications before the initiation of mECT | As specified in the individual report |
|  | General medical comorbid-ities present on or before mECT initiation^a^ | As specified in the individual report |
|  | Medications taken before mECT initiation | As specified in the individual report |
|  | Assisted reproductive technology | Yes (specify), no, unspecified |
| Treatment details | ECT electrode placement | Bitemporal (BT), bifrontal (BF), right unilateral (RUL), left unilateral (LUL), left anterior right temporal (LART), other |
|  | Pulse width | Brief pulse (0.5-2.0 ms), ultrabrief pulse (<0.5 ms), other (specify), unspecified |
|  | Frequency of mECT | As specified in the individual report |
|  | mECT dose | As specified in the individual report |
|  | ECT mode | As specified in the individual report |
| Anesthesia technique | Anesthetic induction agents | Including agent, dose, and route, as specified in the individual report |
|  | Neuromuscular blocking agent(s) | As specified in the individual report, including unspecified and not applied |
|  | Drugs used to manage cardiovascular response to ECT | As specified in the individual report, including unspecified and not applied |
|  | Non-anesthesia medications taken during ECT | As specified in the individual report, excluding anesthesia induction agents and neuromuscular blockers |
|  | Medications taken after ECT | As specified in the individual report |
|  | Airway management | Including mask airway, endotracheal intubation with direct laryngoscopy, endotracheal intubation with video laryngoscopy, supraglottic airway/LMA, other (specify), unspecified |
| Adaptations to ECT technique | Fetal surveillance methods | As specified in the individual report, including pre-, intra-, and post-procedure monitoring |
|  | Adaptations for maternal monitoring | As specified in the individual report, including pre-, intra-, and post-procedure monitoring |
|  | Specific adaptations | Right hip elevation, tilt positioning, cricoid pressure, prehydration, other (specify), unspecified |
|  | Other adaptation(s) | As specified in the individual report, including avoidance of certain medications that are part of standard procedural technique (e.g., avoidance of NSAID use at >20 weeks EGA, etc.) |
| Efficacy/effectiveness | Categorical | Response (or partial response), remission, other |
|  | Time to response/remission | As specified in the individual report, including not reported or not applicable |
|  | Duration of response/remission | As specified in the individual report, including not reported or not applicable |
|  | Maintenance effectiveness | As specified in the individual report |
| Acceptability/safety | Acceptability | As estimated by all-cause dropout rate, dropout owing to adverse effects, and dropout owing to inefficacy, as specified in the individual report |
|  | Adverse maternal events (including severity and time course) | None, preterm vaginal bleeding, contractions without preterm labor/delivery, abdominal/pelvic pain, premature labor, miscarriage, status epilepticus/prolonged seizure, placental abruption, hematuria, pre-eclampsia, cesarean section, maternal mortality, other (specify, including post-ECT headache and cognitive effects), unspecified |
|  | Adverse fetal/neonatal events (including severity and time course) | None, fetal bradycardia, other fetal arrhythmia, preterm delivery, abortion/ stillbirth/neonatal death, congenital malformations/anomalies, fetal growth restriction, fetal/ neonatal respiratory distress, low birth-weight, low APGAR scores (1 minute, 5 minutes), Other fetal/neonatal adverse events (specify), unspecified |
|  | Longer-term adverse events in offspring (including severity and time course) | Delayed unspecified neurocognitive development/ mental retardation, adverse emotional/ neurobehavioral events, motor neuro-developmental delay/abnormalit(ies), specified intellectual disability/disorder, other growth problems, specified psychiatric disorder(s), other (specify), unspecified |
|  |  |  |
| Key: ECT = electroconvulsive therapy; LMA = laryngeal mask airway; MDD = major depressive disorder; mECT = modified electroconvulsive therapy.  ^a^ Specific general medical conditions for abstraction will include myocardial infarction, arrhythmias, heart failure, brain tumors, stroke, arterial hypertension, aneurysm, increased intracranial pressure, chronic obstructive pulmonary disease, pseudocholinesterase deficiency, retinal detachment, glaucoma, and myasthenia gravis. | | |

Supplementary Table 3. Overview of adaptations to standard electroconvulsive therapy (ECT) technique in selected reviewed reports^a^

|  | Obstetrician attendance | Plasmacholinesterase levels | Non-stress testing | Level 2 ultrasound (18-22 wks) | Avoidance of NSAIDs | Pre-ECT antacid(s)/motility drug(s) | Pre-hydration | Re-oxygenation (post-procedure) | Right hip elevation (esp. at 20+ wks) | Other uterine displacement | External FHR monitoring | Continuous FHR monitoring | US for fetal wellbeing | Biophysical profiling | Intubation (esp. in 2^nd^/3^rd^ trimest.) | Supraglottic airway | Pre-oxygenation | Post-stimulus oxygenation | Maternal arterial blood gas | Uterine tocography/dynamometry | Tocolytic therapy | Tocolytic prophylaxis | Anti-nausea/anti-emetic therapy | US verification of empty stomach | Cricoid pressure | Other adatptation(s) |  |
| --- | --- | --- | --- | --- | --- | --- | --- | --- | --- | --- | --- | --- | --- | --- | --- | --- | --- | --- | --- | --- | --- | --- | --- | --- | --- | --- | --- |
| Bak et al. (2016) |  | ● |  |  |  | ● |  |  |  |  | ● |  |  |  |  |  | ● |  |  |  |  |  |  |  |  |  |  |
| Balki et al. (2006) |  |  |  |  |  | ● |  |  | ● |  | ● |  |  |  | ● |  | ● |  |  |  |  |  |  |  | ● |  |  |
| Bez et al. (2013) |  |  |  |  |  |  |  |  | ● |  |  |  |  |  | ● |  |  |  |  |  |  |  |  |  |  |  |  |
| Bhatia et al. (1999) |  |  |  |  |  |  |  |  |  |  | ● |  |  | ● | ● |  |  |  | ● | ● | ● |  |  |  |  |  |  |
| Brown et al. (2003) |  |  |  |  |  | ● |  |  |  |  |  |  |  |  | ● | ● | ● |  |  |  |  |  |  |  |  |  |  |
| DeAsis et al. (2013) |  |  |  |  |  |  |  |  |  |  | ● |  |  |  |  |  |  |  |  |  |  |  |  |  |  | ●^b^ |  |
| DeBattista et al. (2003)^c^ |  |  |  |  |  | ● |  |  |  |  | ● |  |  |  |  |  |  |  |  |  |  |  |  |  | ● |  |  |
| Dorn et al. (1985) | ● |  |  |  |  |  |  |  |  |  | ● |  |  |  |  |  |  |  | ● |  |  |  |  |  |  |  |  |
| EchevarriaMoreno et al. (1998) |  |  |  |  |  |  |  |  |  |  |  |  | ● |  |  |  | ● |  |  |  |  |  |  |  |  |  |  |
| Erturk et al. (2020) |  |  |  |  |  |  |  |  |  |  |  | ● |  |  |  |  |  |  |  |  |  |  |  |  |  |  |  |
| Gahr et al. (2012) |  |  |  |  |  |  |  |  |  |  |  |  | ● |  |  |  |  |  |  |  |  |  |  |  |  |  |  |
| Gannon et al. (2021) |  |  |  |  |  |  |  |  | ● |  | ● | ● |  |  | ●^d^ |  |  |  |  |  |  |  | ● |  |  | ●^b^ |  |
| Gonzalez et all (2014) |  |  |  |  |  |  |  |  |  |  |  | ● |  |  |  |  |  |  |  | ● |  |  |  |  |  |  |  |
| Griffiths et al. (1989) |  |  |  |  |  |  |  |  |  |  |  | ● |  |  |  |  |  |  |  | ● |  |  |  |  |  |  |  |
| Gunduz et al. (2010) |  |  |  |  |  |  | ● |  | ● |  |  | ● |  |  |  |  |  |  |  |  |  |  |  |  |  |  |  |
| Iwasaki et al. (2002) |  |  | ● |  |  |  |  |  |  |  |  |  |  |  |  |  |  |  |  |  |  |  |  |  |  | ●^b^ |  |
| Livingston et al. (1994) |  |  | ● |  |  |  | ● |  |  | ● | ● |  |  | ● |  |  |  |  |  | ● |  |  |  |  |  | ●^e^ |  |
| Malhotra et al. (2008) |  |  |  |  |  | ● | ● |  | ● |  |  |  |  |  | ● |  | ● |  |  |  |  | ● |  |  |  | ●^f^ |  |
| O’Reardon et al. (2011) |  |  | ● |  |  | ● | ● |  | ● |  | ● |  |  |  |  |  |  |  |  | ● |  |  | ● |  | ● |  |  |
| Patel et al. (2022) |  |  |  |  |  |  |  |  | ● |  | ● |  |  |  | ● |  |  |  |  |  |  |  |  |  |  |  |  |
| Pesiridou et al. (2010) |  |  | ● |  |  |  |  |  | ● |  |  |  |  |  |  |  |  |  |  | ● |  |  |  |  |  |  |  |
| Pierre et al. (2020) |  |  |  | ● |  | ● |  |  |  |  | ● |  |  |  |  |  |  |  |  |  |  |  |  | ● |  |  |  |
| Pinette et al. (2007) |  |  |  |  |  |  |  |  |  |  | ● |  |  |  |  |  |  |  |  |  |  |  |  |  |  |  |  |
| Rabie et al. (2021) |  |  |  |  |  |  |  |  |  |  | ● | ● |  |  | ● |  |  |  |  |  |  |  |  |  |  |  |  |
| Ray-Griffith et al. (2016) |  |  |  |  |  | ● |  |  |  |  | ● |  |  |  |  |  |  |  |  |  |  |  | ● |  |  |  |  |
| Repke et al. (1984) |  |  |  |  |  |  |  |  |  |  |  | ● | ● |  | ● |  |  |  |  |  |  |  |  |  |  |  |  |
| Richardson et al. (2018) | ● |  |  |  |  |  |  |  | ● |  |  |  |  |  | ● |  |  |  |  |  |  |  |  |  |  | ●^b^ |  |
| Rineh et al. (2020) | ● |  |  |  |  | ● | ● |  |  | ● | ● |  |  |  |  |  | ● |  |  |  |  | ● |  |  |  |  |  |
| Salzbrenner et al. (2011) |  |  |  |  |  | ● |  |  |  |  | ● |  |  |  |  |  |  |  |  |  |  |  | ● |  |  | ●^b^ |  |
| Serim et al. (2010) |  | ● |  |  |  |  |  |  | ● |  | ● |  |  |  |  |  |  |  |  |  | ● |  |  |  |  |  |  |
| Sherer et al. (1991) |  |  |  |  |  |  | ● |  |  |  |  | ● |  |  | ● |  |  |  |  | ● | ● |  |  |  |  | ●^e^ |  |
| Walker et al. (1992) |  |  |  |  |  | ● |  |  |  | ● | ● |  |  |  |  |  |  |  |  | ● | ● |  |  |  | ● |  |  |
| Watanabe et al. (2019) | ● |  |  |  |  |  |  | ● |  |  | ● |  | ● |  |  |  |  |  |  |  |  |  |  |  |  |  |  |
| Wise et al. (1984) | ● |  |  |  |  |  |  |  |  |  | ● |  |  |  | ● |  |  |  |  | ● |  |  |  |  |  | ●^b^ |  |
| Yang et al. (2011) |  |  |  |  |  |  |  |  | ● |  | ● |  | ● |  |  |  |  |  |  | ● | ● |  |  |  |  |  |  |
| Key: EGA = gestational age/estimated gestational age; US = ultrasound.  ^a^ Table 3 table summarizes information on documented pre-, peri-, and post-procedural adaptations to standard ECT technique (beyond elements of the standard pre-ECT workup, pre-procedure NPO status and routine electroencephalogram, electrocardiogram, external blood pressure, oxygen saturation, and end-tidal CO_2_ monitoring procedures). Only reports in which such procedures were clearly described are included. ^b^ Emergent cesarean section capabilities were immediately available. ^c^ Hyperventilation was used before delivering the ECT stimulus for a treatment series starting at 17 weeks EGA. ^d^ Additional precautions were taken owing to COVID-19 infection, including the use of N95 masks and eye protection in addition to standard protective equipment procedures, as well as video laryngoscopy for added aerosolization exposure risk reduction. ^e^ Periodic Doppler flow velocimetry of the uterine and umbilical arteries. ^f^ Fetal cardiometry. | | | | | | | | | | | | | | | | | | | | | | | | | | | |

Appendix. Final Search Strategies

**Database:** Ovid MEDLINE ALL (1946-present)

**Search Date:** December 31, 2024

**Limits:** English language; human studies

**Number of Results:** 316

1 (pregnan* or parous or parity or parities or primipar* or multiparit* or multiparous or multipara or primigravid* or multigravid* or gestat* or gravidit* or parturi* or puerper* or perinatal* or peri natal* or prenatal* or pre natal* or antenatal* or ante natal* or postnatal* or post natal* or peripartum or peri partum or prepartum or pre partum or antepartum or ante partum or intrapartum or intra partum or postpartum or post partum or fertility or fertile or obstetric* or reproduction or reproductive or maternit* or maternal or parturient* or gravidit* or perinatal* or peri natal* or (expectant* adj2 mother*) or mother-to-be or mothers-to-be or (child adj1 bear*) or childbear* or periconception* or peri conception* or ((before or after or vaginal or abdominal) adj2 delivery) or childbirth* or child-birth* or (child adj5 (birth or delivery))).tw,kf,ot. 1602015

2 (embryo* or fetus* or foetus* or fetal* or foetal* or placenta* or transplacenta* or trans-placenta* or utero* or uterus* or ((preterm or pre-term or premature or term or live or still) adj (birth or births or childbirth* or child-birth* or infant* or newborn* or new-born* or neonate* or neonatal* or neo-natal* or baby or babies or labor* or labour* or parturition* or parturient* or delivery or deliveries)) or prematurity or stillbirth* or still-birth* or stillborn* or still-born* or cesarean* or caesarean* or cesarian* or caesarian* or cesarien* or caesarien* or C-section* or abortion* or aborted or miscarr* or birthweight* or birth-weight* or "gestational age" or "gestational hypertension" or eclampsia* or preeclampsia* or pre eclampsia* or Apgar or ((infant* or newborn* or new-born* or neonate* or baby or babies or mother* or intrauterine or intra-uterine) adj1 (mortality or mortalities or dead or death* or loss or lost or demise* or viability))).ti,ab. 1221258

3 (((congenital* or birth* or development*) adj1 (disorder* or abnormalit* or defect* or malform* or deform* or disease* or syndrom*)) or foetopath* or fetopath* or foeto-path* or feto-path* or ((mental or growth or psychomotor) adj1 retardation) or amniocentes* or chorion* vill* or tocoly*).ti,ab. 162418

4 (breastfe* or breast-fe* or breastmilk* or breast-milk* or "breast pumping*" or (milk adj1 (express* or excret* or releas* or secret*)) or lactat* or ((human or breast or mother or woman or women or maternal) adj milk*)).ti,ab. 266602

5 Pregnant Women/ or exp Perinatal Care/ or Postpartum Period/ or Prenatal Care/ or Pregnancy/ or Gravidity/ or Parity/ or exp Parturition/ or Pregnancy in Adolescence/ or Maternal-Fetal Exchange/ or exp Pregnancy Outcome/ or Pregnancy, High-Risk/ or exp Pregnancy, Multiple/ or Pregnancy, Unplanned/ or Pregnancy, Unwanted/ or Prenatal Nutritional Physiological Phenomena/ or Pseudopregnancy/ or Peripartum Period/ or exp Pregnancy Trimesters/ or exp Pregnancy Complications/ or exp Delivery, Obstetric/ or exp Labor, Obstetric/ or Prenatal Education/ or Perinatology/ or Maternal Exposure/ or Placental Circulation/ or Breast Milk Expression/ or Milk, Human/ or Congenital Abnormalities/ or Prenatal Exposure Delayed Effects/ or Maternal Health/ or Maternal Health Services/ or exp Labor Pain/ or exp Analgesia, Obstetrical/ or exp Anesthesia, Obstetrical/ or exp Obstetric Surgical Procedures/ or exp Infant, Newborn/ or exp Prenatal Diagnosis/ or Obstetrics/ 1641268

6 or/1-5 3070573

7 exp "Depression, Postpartum"/ 8258

8 (depress* or dysphor* or dysthymi* or melanchol*).tw,kf,ot. 625263

9 (season* adj2 (affective or mood* or depress*)).tw,kf. 1830

10 ((bipolar or bi-polar or mood* or affective or manic-depress* or manic) adj2 (disorder* or psychosis or psychoses or psychotic or symptom*)).tw,kf. 88653

11 (involutional adj2 (psychosis or psychoses or psychotic or paraphreni*)).tw,kf. 274

12 exp Depression/ or "Depressive Disorder"/ or "Depressive Disorder, Major"/ or "Depressive Disorder, Treatment-Resistant"/ or "Dysthymic Disorder"/ or "Seasonal Affective Disorder"/ or "Mood Disorders"/ or exp "Bipolar and Related Disorders"/ or "Affective Disorders, Psychotic"/ 315373

13 or/8-12 722264

14 6 and 13 61542

15 7 or 14 62210

16 exp Electroconvulsive Therapy/ 14665

17 (electroconvuls* or electro-convuls* or electrovuls* or electro-vuls* or electroshock* or ECT or (electr* adj2 (convuls* or shock*))).tw,kf,ot. 29018

18 16 or 17 32829

19 15 and 18 389

20 exp animals/ not humans.sh. 5292681

21 19 not 20 365

22 21 and english.lg. 316

**Database:** Embase.com (Elsevier) (1947-Current)

**Search Date:** December 31, 2024

**Limits:** English language; human studies

**Number of Results:** 1,109

1 (pregnan* OR parous OR parity OR parities OR primipar* OR multiparit* OR multiparous OR multipara OR primigravid* OR multigravid* OR gestat* OR gravidit* OR parturi* OR puerper* OR perinatal* OR "peri natal*" OR prenatal* OR "pre natal*" OR antenatal* OR "ante natal*" OR postnatal* OR "post natal*" OR peripartum OR "peri partum" OR prepartum OR "pre partum" OR antepartum OR "ante partum" OR intrapartum OR "intra partum" OR postpartum OR "post partum" OR fertility OR fertile OR obstetric* OR reproduction OR reproductive OR maternit* OR maternal OR parturient* OR gravidit* OR perinatal* OR "peri natal*" OR (expectant* NEAR/2 mother*) OR mother-to-be OR mothers-to-be OR (child NEAR/1 bear*) OR childbear* OR periconception* OR "peri conception*" OR ((before OR after OR vaginal OR abdominal) NEAR/2 delivery) OR childbirth* OR child-birth* OR (child NEAR/2 (birth OR delivery))):TI,AB,KW

2 (embryo* OR fetus* OR foetus* OR fetal* OR foetal* OR placenta* OR transplacenta* OR trans-placenta* OR utero* OR uterus* OR ((preterm OR pre-term OR premature OR term OR live OR still) NEAR (birth OR births OR childbirth* OR child-birth* OR infant* OR newborn* OR new-born* OR neonate* OR neonatal* OR neo-natal* OR baby OR babies OR labor* OR labour* OR parturition* OR parturient* OR delivery OR deliveries)) OR prematurity OR stillbirth* OR still-birth* OR stillborn* OR still-born* OR cesarean* OR caesarean* OR cesarian* OR caesarian* OR cesarien* OR caesarien* OR C-section* OR abortion* OR aborted OR miscarr* OR birthweight* OR birth-weight* OR "gestational age" OR "gestational hypertension" OR eclampsia* OR preeclampsia* OR pre eclampsia* OR Apgar OR ((infant* OR newborn* OR new-born* OR neonate* OR baby OR babies OR mother* OR intrauterine OR intra-uterine) NEAR/1 (mortality OR mortalities OR dead OR death* OR loss OR lost OR demise* OR viability))):TI,AB

3 (((congenital* OR birth* OR development*) NEAR/1 (disorder* OR abnormalit* OR defect* OR malform* OR deform* OR disease* OR syndrom*)) OR foetopath* OR fetopath* OR foeto-path* OR feto-path* OR ((mental OR growth OR psychomotor) adj1 retardation) OR amniocentes* OR chorion* vill* OR tocoly*):TI,AB

4 (breastfe* OR breast-fe* OR breastmilk* OR breast-milk* OR "breast pumping*" OR (milk adj1 (express* OR excret* OR releas* OR secret*)) OR lactat* OR ((human OR breast OR mother OR woman OR women OR maternal) NEAR milk*)):TI,AB

5 "pregnant woman"/exp OR "perinatal care"/exp OR "puerperium"/exp OR "prenatal care"/exp OR "pregnancy"/exp OR "intrapartum care"/de OR "parity"/de OR "birth"/de OR "fetomaternal transfusion"/de OR "parameters concerning the fetus, newborn and pregnancy"/exp OR "pregnancy disorder"/exp OR "pseudopregnancy"/de OR "perinatal period"/de OR "obstetric delivery"/exp OR "obstetric procedure"/de OR "childbirth"/exp OR "prenatal development"/exp OR "childbirth education"/de OR "prenatal diagnosis"/exp OR "prenatal exposure"/exp OR "maternal exposure"/de OR "placenta circulation"/de OR "fetus circulation"/de OR "breast feeding"/exp OR "infant feeding"/de OR "breast milk"/de OR 'congenital disorder'/exp OR "maternal child health care"/de OR "maternal health service"/de OR "labor pain"/de OR "obstetric anesthesia"/exp OR "obstetric operation"/exp OR 'infant'/exp OR "obstetrics"/exp

6 #1 OR #2 OR #3 OR #4 OR #5

7 "perinatal depression"/exp OR "puerperal psychosis"/de

8 (depress* OR dysphor* OR dysthymi* OR melanchol*):TI,AB,KW

9 (season* NEAR/2 (affective OR mood* OR depress*)):TI,AB,KW

10 ((bipolar OR bi-polar OR mood* OR affective OR manic-depress* OR manic) NEAR/2 (disorder* OR psychosis OR psychoses OR psychotic OR symptom*)):TI,AB,KW

11 (involutional NEAR/2 (psychosis OR psychoses OR psychotic OR paraphreni*)):TI,AB,KW

12 "mood disorder"/de OR "affective neurosis"/de OR "affective psychosis"/de OR "blunted affect"/de OR "major affective disorder"/de OR "mania"/exp OR "minor affective disorder"/de OR "depression"/exp OR "depressive psychosis"/de OR "catatonia"/de

13 #8 OR #9 OR #10 OR #11 OR #12

14 #6 AND #13

15 #7 OR #14

16 "electroconvulsive therapy"/exp

17 (electroconvuls* OR electro-convuls* OR electrovuls* OR electro-vuls* OR electroshock* OR ECT OR ((electric* OR electrode*) NEAR/2 (convuls* OR shock*))):TI,AB,KW

18 #16 OR #17

19 #15 AND #18

20 #19 AND english:la

21 ((rat:ti,tt OR rats:ti,tt OR mouse:ti,tt OR mice:ti,tt OR swine:ti,tt OR porcine:ti,tt OR murine:ti,tt OR sheep:ti,tt OR lambs:ti,tt OR pigs:ti,tt OR piglets:ti,tt OR rabbit:ti,tt OR rabbits:ti,tt OR cat:ti,tt OR cats:ti,tt OR dog:ti,tt OR dogs:ti,tt OR cattle:ti,tt OR bovine:ti,tt OR monkey:ti,tt OR monkeys:ti,tt OR trout:ti,tt OR marmoset*:ti,tt) AND 'animal experiment'/de)

22 ('animal experiment'/de NOT ('human experiment'/de OR 'human'/de))

23 #21 OR #22

24 #20 NOT #23

**Database:** Cochrane Library (Cochrane Database of Systematic Reviews [CDSR]; Cochrane Central Register of Controlled Trials [CENTRAL]) (Wiley)

**Search Date:** December 31, 2024

**Limits:** English language

**Number of Results:** 114

1 (pregnan* OR parous OR parity OR parities OR primipar* OR multiparit* OR multiparous OR multipara OR primigravid* OR multigravid* OR gestat* OR gravidit* OR parturi* OR puerper* OR perinatal* OR peri natal* OR prenatal* OR pre natal* OR antenatal* OR ante natal* OR postnatal* OR post natal* OR peripartum OR peri partum OR prepartum OR pre partum OR antepartum OR ante partum OR intrapartum OR intra partum OR postpartum OR post partum OR fertility OR fertile OR obstetric* OR reproduction OR reproductive OR maternit* OR maternal OR parturient* OR gravidit* OR perinatal* OR peri natal* OR (expectant* NEAR/2 mother*) OR mother-to-be OR mothers-to-be OR (child NEAR/1 bear*) OR childbear* OR periconception* OR peri conception* OR ((before OR after OR vaginal OR abdominal) NEAR/2 delivery) OR childbirth* OR child-birth* OR (child NEAR/5 (birth OR delivery))):TI,AB,KW

2 (embryo* OR fetus* OR foetus* OR fetal* OR foetal* OR placenta* OR transplacenta* OR trans-placenta* OR utero* OR uterus* OR ((preterm OR pre-term OR premature OR term OR live OR still) adj (birth OR births OR childbirth* OR child-birth* OR infant* OR newborn* OR new-born* OR neonate* OR neonatal* OR neo-natal* OR baby OR babies OR labor* OR labour* OR parturition* OR parturient* OR delivery OR deliveries)) OR prematurity OR stillbirth* OR still-birth* OR stillborn* OR still-born* OR cesarean* OR caesarean* OR cesarian* OR caesarian* OR cesarien* OR caesarien* OR C-section* OR abortion* OR aborted OR miscarr* OR birthweight* OR birth-weight* OR "gestational age" OR "gestational hypertension" OR eclampsia* OR preeclampsia* OR pre eclampsia* OR Apgar OR ((infant* OR newborn* OR new-born* OR neonate* OR baby OR babies OR mother* OR intrauterine OR intra-uterine) NEAR/1 (mortality OR mortalities OR dead OR death* OR loss OR lost OR demise* OR viability))):TI,AB

3 (((congenital* OR birth* OR development*) adj1 (disorder* OR abnormalit* OR defect* OR malform* OR deform* OR disease* OR syndrom*)) OR foetopath* OR fetopath* OR foeto-path* OR feto-path* OR ((mental OR growth OR psychomotor) NEAR/1 retardation) OR amniocentes* OR chorion* vill* OR tocoly*):TI,AB

4 (breastfe* OR breast-fe* OR breastmilk* OR breast-milk* OR breast pumping* OR (milk NEXT (express* OR excret* OR releas* OR secret*)) OR lactat* OR ((human OR breast OR mother OR woman OR women OR maternal) NEXT milk*)):TI,AB

5 [mh ^"Pregnant Women"] OR [mh "Perinatal Care"] OR [mh ^"Postpartum Period"] OR [mh ^"Prenatal Care"] OR [mh ^Pregnancy] OR [mh ^Gravidity] OR [mh ^Parity] OR [mh Parturition] OR [mh ^"Pregnancy in Adolescence"] OR [mh ^"Maternal-Fetal Exchange"] OR [mh "Pregnancy Outcome"] OR [mh ^"Pregnancy, High-Risk"] OR [mh "Pregnancy, Multiple"] OR [mh ^"Pregnancy, Unplanned"] OR [mh ^"Pregnancy, Unwanted"] OR [mh ^"Prenatal Nutritional Physiological Phenomena"] OR [mh ^Pseudopregnancy] OR [mh ^"Peripartum Period"] OR [mh "Pregnancy Trimesters"] OR [mh "Pregnancy Complications"] OR [mh "Delivery, Obstetric"] OR [mh "Labor, Obstetric"] OR [mh ^"Prenatal Education"] OR [mh ^Perinatology] OR [mh ^"Maternal Exposure"] OR [mh ^"Placental Circulation"] OR [mh ^"Breast Feeding"] OR [mh ^"Breast Milk Expression"] OR [mh ^"Milk, Human"] OR [mh ^"Congenital Abnormalities"] OR [mh ^"Prenatal Exposure Delayed Effects"] OR [mh ^"Maternal Health"] OR [mh ^"Maternal Health Services"] OR [mh "Labor Pain"] OR [mh "Analgesia, Obstetrical"] OR [mh "Anesthesia, Obstetrical"] OR [mh "Obstetric Surgical Procedures"] OR [mh "Infant, Newborn"] OR [mh "Prenatal Diagnosis"] OR [mh ^Obstetrics]

6 #1 OR #2 OR #3 OR #4 OR #5

7 [mh "Depression, Postpartum"]

8 (depress* OR dysphor* OR dysthymi* OR melanchol*):TI,AB,KW

9 (season* NEAR/2 (affective OR mood* OR depress*)):TI,AB,KW

10 ((bipolar OR bi-polar OR mood* OR affective OR manic-depress* OR manic) NEAR/2 (disorder* OR psychosis OR psychoses OR psychotic OR symptom*)):TI,AB,KW

11 (involutional NEAR/2 (psychosis OR psychoses OR psychotic OR paraphreni*)):TI,AB,KW

12 [mh Depression] OR [mh ^"Depressive Disorder"] OR [mh ^"Depressive Disorder, Major"] OR [mh ^"Depressive Disorder, Treatment-Resistant"] OR [mh ^"Dysthymic Disorder"] OR [mh ^"Seasonal Affective Disorder"] OR [mh ^"Mood Disorders"] OR [mh "Bipolar and Related Disorders"] OR [mh ^"Affective Disorders, Psychotic"]

13 #8 OR #9 OR #10 OR #11 OR #12

14 #6 AND #13

15 #7 OR #14

16 [mh "Electroconvulsive Therapy"]

17 (electroconvuls* OR electro-convuls* OR electrovuls* OR electro-vuls* OR electroshock* OR ECT OR (electr* NEAR/2 (convuls* OR shock*))):TI,AB,KW

18 #16 OR #17

19 #15 AND #18

20 #19 AND English:la

**Database:** APA PsycINFO (ProQuest) (1894–Current)

**Search Date:** December 31, 2024

**Limits:** English language; human studies

**Number of Results:** 267

1 TI,AB,IF(pregnan* OR parous OR parity OR parities OR primipar* OR multiparit* OR multiparous OR multipara OR primigravid* OR multigravid* OR gestat* OR gravidit* OR parturi* OR puerper* OR perinatal* OR "peri natal*" OR prenatal* OR "pre natal*" OR antenatal* OR "ante natal*" OR postnatal* OR "post natal*" OR peripartum OR "peri partum" OR prepartum OR "pre partum" OR antepartum OR "ante partum" OR intrapartum OR "intra partum" OR postpartum OR "post partum" OR fertility OR fertile OR obstetric* OR reproduction OR reproductive OR maternit* OR maternal OR parturient* OR gravidit* OR perinatal* OR "peri natal*" OR (expectant* NEAR/2 mother*) OR mother-to-be OR mothers-to-be OR (child NEAR/1 bear*) OR childbear* OR periconception* OR peri conception* OR ((before OR after OR vaginal OR abdominal) NEAR/2 delivery) OR childbirth* OR child-birth* OR (child NEAR/5 (birth OR delivery)))

2 TI,AB(embryo* OR fetus* OR foetus* OR fetal* OR foetal* OR placenta* OR transplacenta* OR trans-placenta* OR utero* OR uterus* OR ((preterm OR pre-term OR premature OR term OR live OR still) NEAR (birth OR births OR childbirth* OR child-birth* OR infant* OR newborn* OR new-born* OR neonate* OR neonatal* OR neo-natal* OR baby OR babies OR labor* OR labour* OR parturition* OR parturient* OR delivery OR deliveries)) OR prematurity OR stillbirth* OR still-birth* OR stillborn* OR still-born* OR cesarean* OR caesarean* OR cesarian* OR caesarian* OR cesarien* OR caesarien* OR C-section* OR abortion* OR aborted OR miscarr* OR birthweight* OR birth-weight* OR "gestational age" OR "gestational hypertension" OR eclampsia* OR preeclampsia* OR "pre eclampsia*" OR Apgar OR ((infant* OR newborn* OR new-born* OR neonate* OR baby OR babies OR mother* OR intrauterine OR intra-uterine) NEAR (mortality OR mortalities OR dead OR death* OR loss OR lost OR demise* OR viability)))

3 TI,AB(((congenital* OR birth* OR development*) NEAR (disorder* OR abnormalit* OR defect* OR malform* OR deform* OR disease* OR syndrom*)) OR foetopath* OR fetopath* OR foeto-path* OR feto-path* OR ((mental OR growth OR psychomotor) NEAR retardation) OR amniocentes* OR chorion* vill* OR tocoly*)

4 TI,AB(breastfe* OR breast-fe* OR breastmilk* OR breast-milk* OR "breast pumping" OR (milk NEAR/1 (express* OR excret* OR releas* OR secret*)) OR lactat* OR ((human OR breast OR mother OR woman OR women OR maternal) NEAR/1 milk*))

5 MAINSUBJECT.EXACT.EXPLODE("Pregnancy" OR "Prenatal Care" OR "Mother Child Relations" OR "Prenatal Exposure" OR "Prenatal Diagnosis" OR "Perinatal Period" OR "Placenta" OR "Pseudocyesis" OR "Breast Feeding" OR "Congenital Disorders" OR "Expectant Mothers" OR "Obstetrics" OR "Infant Development")

6 [S1] OR [S2] OR [S3] OR [S4] OR [S5]

7 MAINSUBJECT.EXACT("Postpartum Depression")

8 TI,AB,IF(depress* OR dysphor* OR dysthymi* OR melanchol*)

9 TI,AB,IF(season* NEAR/2 (affective OR mood* OR depress*))

10 TI,AB,IF((bipolar OR bi-polar OR mood* OR affective OR manic-depress* OR manic) NEAR/2 (disorder* OR psychosis OR psychoses OR psychotic OR symptom*))

11 TI,AB,IF(involutional NEAR/2 (psychosis OR psychoses OR psychotic OR paraphreni*))

12 MAINSUBJECT.EXACT.EXPLODE("Affective Disorders" OR "Bipolar Disorder" OR "Affective Psychosis" OR "Postpartum Psychosis" )

13 [S8] OR [S9] OR [S10] OR [S11] OR [S12]

14 [S6] AND [S13]

15 [S7] OR [S14]

16 MAINSUBJECT.EXACT.EXPLODE("Electroconvulsive Shock")

17 TI,AB,IF(electroconvuls* OR electro-convuls* OR electrovuls* OR electro-vuls* OR electroshock* OR ECT OR (electr* NEAR/2 (convuls* OR shock*)))

18 [S16] OR [S17]

19 [S15] AND [S18]

20 LA("English")

21 MAINSUBJECT.EXACT.EXPLODE("animals")

22 MAINSUBJECT.EXACT.EXPLODE("animal research")

23 MAINSUBJECT.EXACT.EXPLODE("animal models")

24 MAINSUBJECT("nonhuman")

25 MAINSUBJECT.EXACT.EXPLODE("vertebrates")

26 POP("animal")

27 [S21] OR [S22] OR [S23] OR [S24] OR [S25] OR [S26]

28 MAINSUBJECT("humans")

29 MAINSUBJECT("human experimentation" OR "human experiment")

30 POP("human")

31 [S28] OR [29] OR [S30]

32 [S27] NOT [S31]

33 [S19] AND [S20]

34 [S33] NOT [S32]

**Database:** Web of Science - Science Citation Index Expanded (SCI-EXPANDED) –1900-present, Social Sciences Citation Index (SSCI)—1956-present, Conference Proceedings Citation Index – Science (CPCI-S)—1993-present, Conference Proceedings Citation Index – Social Science & Humanities (CPCI-SSH)—1993-present, BIOSIS Citation Index—1969-present

**Search Date:** December 31, 2024

**Limits:** English language; human studies

**Number of Results:** 435 (SCI-EXPANDED, SSCI, CPCI-S, CPCI-SSH: 297; BIOSIS: 138 BIOSIS)

1 TS=(pregnan* OR parous OR parity OR parities OR primipar* OR multiparit* OR multiparous OR multipara OR primigravid* OR multigravid* OR gestat* OR gravidit* OR parturi* OR puerper* OR perinatal* OR peri natal* OR prenatal* OR pre natal* OR antenatal* OR ante natal* OR postnatal* OR post natal* OR peripartum OR peri partum OR prepartum OR pre partum OR antepartum OR ante partum OR intrapartum OR intra partum OR postpartum OR post partum OR fertility OR fertile OR obstetric* OR reproduction OR reproductive OR maternit* OR maternal OR parturient* OR gravidit* OR perinatal* OR peri natal* OR (expectant* NEAR/2 mother*) OR mother-to-be OR mothers-to-be OR (child NEAR/1 bear*) OR childbear* OR periconception* OR peri conception* OR ((before OR after OR vaginal OR abdominal) NEAR/2 delivery) OR childbirth* OR child-birth* OR (child NEAR/5 (birth OR delivery)))

2 TI=(embryo* OR fetus* OR foetus* OR fetal* OR foetal* OR placenta* OR transplacenta* OR trans-placenta* OR utero* OR uterus* OR ((preterm OR pre-term OR premature OR term OR live OR still) NEAR (birth OR births OR childbirth* OR child-birth* OR infant* OR newborn* OR new-born* OR neonate* OR neonatal* OR neo-natal* OR baby OR babies OR labor* OR labour* OR parturition* OR parturient* OR delivery OR deliveries)) OR prematurity OR stillbirth* OR still-birth* OR stillborn* OR still-born* OR cesarean* OR caesarean* OR cesarian* OR caesarian* OR cesarien* OR caesarien* OR C-section* OR abortion* OR aborted OR miscarr* OR birthweight* OR birth-weight* OR "gestational age" OR "gestational hypertension" OR eclampsia* OR preeclampsia* OR pre eclampsia* OR Apgar OR ((infant* OR newborn* OR new-born* OR neonate* OR baby OR babies OR mother* OR intrauterine OR intra-uterine) NEAR/1 (mortality OR mortalities OR dead OR death* OR loss OR lost OR demise* OR viability))) OR AB=(embryo* OR fetus* OR foetus* OR fetal* OR foetal* OR placenta* OR transplacenta* OR trans-placenta* OR utero* OR uterus* OR ((preterm OR pre-term OR premature OR term OR live OR still) NEAR (birth OR births OR childbirth* OR child-birth* OR infant* OR newborn* OR new-born* OR neonate* OR neonatal* OR neo-natal* OR baby OR babies OR labor* OR labour* OR parturition* OR parturient* OR delivery OR deliveries)) OR prematurity OR stillbirth* OR still-birth* OR stillborn* OR still-born* OR cesarean OR caesarean OR cesarian OR caesarian OR cesarien OR caesarien* OR C-section* OR abortion* OR aborted OR miscarr* OR birthweight* OR birth-weight* OR "gestational age" OR "gestational hypertension" OR eclampsia* OR preeclampsia* OR pre eclampsia* OR Apgar OR ((infant* OR newborn* OR new-born* OR neonate* OR baby OR babies OR mother* OR intrauterine OR intra-uterine) NEAR/1 (mortality OR mortalities OR dead OR death* OR loss OR lost OR demise* OR viability)))

3 TI=(((congenital* OR birth* OR development*) NEAR/1 (disorder* OR abnormalit* OR defect* OR malform* OR deform* OR disease* OR syndrom*)) OR foetopath* OR fetopath* OR foeto-path* OR feto-path* OR ((mental OR growth OR psychomotor) NEAR/1 retardation) OR amniocentes* OR chorion* vill* OR tocoly*) OR AB=(((congenital* OR birth* OR development*) NEAR/1 (disorder* OR abnormalit* OR defect* OR malform* OR deform* OR disease* OR syndrom*)) OR foetopath* OR fetopath* OR foeto-path* OR feto-path* OR ((mental OR growth OR psychomotor) NEAR/1 retardation) OR amniocentes* OR chorion* vill* OR tocoly*)

4 TI=(breastfe* OR breast-fe* OR breastmilk* OR breast-milk* OR "breast pumping*" OR (milk NEAR/1 (express* OR excret* OR releas* OR secret*)) OR lactat* OR ((human OR breast OR mother OR woman OR women OR maternal) NEAR milk*)) OR AB=(breastfe* OR breast-fe* OR breastmilk* OR breast-milk* OR "breast pumping*" OR (milk NEAR/1 (express* OR excret* OR releas* OR secret*)) OR lactat* OR ((human OR breast OR mother OR woman OR women OR maternal) NEAR milk*))

5 #1 OR #2 OR #3 OR #4

6 TS=(depress* OR dysphor* OR dysthymi* OR melanchol*)

7 TS=(season* NEAR/2 (affective OR mood* OR depress*))

8 TS=((bipolar OR bi-polar OR mood* OR affective OR manic-depress* OR manic) NEAR/2 (disorder* OR psychosis OR psychoses OR psychotic OR symptom*))

9 TS=(involutional NEAR/2 (psychosis OR psychoses OR psychotic OR paraphrenia*))

10 #6 OR #7 OR #8 OR #9

11 TS=(electroconvuls* OR electro-convuls* OR electrovuls* OR electro-vuls* OR electroshock* OR ECT OR (electr* NEAR/2 (convuls* OR shock*)))

12 #5 AND #10 AND #11

13 TS=((animal* OR rat OR rats OR mouse OR mice OR murine OR dog OR dogs OR canine OR cat OR cats OR feline OR rabbit OR cow OR cows OR bovine OR rodent* OR sheep OR ovine OR pig OR swine OR porcine OR veterinar* OR chick* OR zebrafish* OR baboon* OR nonhuman* OR primate* OR cattle* OR goose OR geese OR duck OR macaque* OR avian* OR bird* OR fish*) NOT (human* OR patient* OR women OR woman OR men OR man))

14 #12 NOT #13

15 LA=(English)

16 #14 AND #15

**Database:** CINAHL Ultimate (EBSCO) (1937–Current)

**Search Date:** December 31, 2024

**Limits:** English language; human studies

**Number of Results:** 139

1 TI (pregnant OR pregnants OR pregnancy OR pregnancies OR parous OR parities OR parities OR primiparity OR primiparities OR primiparous OR multiparity OR multiparities OR multiparous OR multipara OR primigravidity OR primigravidities OR multigravidity OR multigravidities OR gestation OR gestate OR gestates OR gestated OR gestating OR gestational OR gravidity OR gravidities OR parturition OR parturitions OR parturitional OR parturient OR puerperal OR puerperally OR puerperant OR puerperants OR perinatal OR perinatally OR perinatals OR peri natal OR peri natally OR peri natals OR prenatal OR prenatally OR prenatals OR pre natal OR pre natally OR pre natals OR antenatal OR antenatally OR antenatals OR ante natal OR ante natally OR ante natals OR postnatal OR postnatally OR postnatals OR post natal OR post natally OR post natals OR peripartum OR peri partum OR prepartum OR pre partum OR antepartum OR ante partum OR intrapartum OR intra partum OR postpartum OR post partum OR fertility OR fertile OR obstetric OR obstetrically OR obstetrics OR obstetrical OR reproduction OR reproductive OR maternity OR maternities OR maternal OR maternally OR parturient OR parturients OR gravidity OR gravidities OR perinatal OR perinatally OR perinatals OR peri natal OR peri natally OR peri natals OR (expectant N2 mother) OR (expectant N2 mothers) OR mother-to-be OR mothers-to-be OR (child N1 bearing) OR (child N1 bearer) OR (child N1 bearers) OR childbearing OR childbearer OR childbearers OR periconception OR periconceptional OR periconceptionally OR peri conception OR peri conceptional OR peri conceptionally OR ((before OR after OR vaginal OR abdominal) N2 delivery) OR childbirth OR childbirths OR child-birth OR child-births OR (child N5 (birth OR delivery))) OR AB (pregnant OR pregnants OR pregnancy OR pregnancies OR parous OR parities OR parities OR primiparity OR primiparities OR primiparous OR multiparity OR multiparities OR multiparous OR multipara OR primigravidity OR primigravidities OR multigravidity OR multigravidities OR gestation OR gestate OR gestates OR gestated OR gestating OR gestational OR gravidity OR gravidities OR parturition OR parturitions OR parturitional OR parturient OR puerperal OR puerperally OR puerperant OR puerperants OR perinatal OR perinatally OR perinatals OR peri natal OR peri natally OR peri natals OR prenatal OR prenatally OR prenatals OR pre natal OR pre natally OR pre natals OR antenatal OR antenatally OR antenatals OR ante natal OR ante natally OR ante natals OR postnatal OR postnatally OR postnatals OR post natal OR post natally OR post natals OR peripartum OR peri partum OR prepartum OR pre partum OR antepartum OR ante partum OR intrapartum OR intra partum OR postpartum OR post partum OR fertility OR fertile OR obstetric OR obstetrically OR obstetrics OR obstetrical OR reproduction OR reproductive OR maternity OR maternities OR maternal OR maternally OR parturient OR parturients OR gravidity OR gravidities OR perinatal OR perinatally OR perinatals OR peri natal OR peri natally OR peri natals OR (expectant N2 mother) OR (expectant N2 mothers) OR mother-to-be OR mothers-to-be OR (child N1 bearing) OR (child N1 bearer) OR (child N1 bearers) OR childbearing OR childbearer OR childbearers OR periconception OR periconceptional OR periconceptionally OR peri conception OR peri conceptional OR peri conceptionally OR ((before OR after OR vaginal OR abdominal) N2 delivery) OR childbirth OR childbirths OR child-birth OR child-births OR (child N5 (birth OR delivery)))

2 TI ("embryo s" OR embryos OR embryonic OR embryo OR fetus OR fetuses OR "fetus s" OR foetu OR foetus OR foetuses OR "foetus s" OR fetal OR fetale OR fetally OR fetals OR foetal OR foetally OR foetals OR placenta OR placentas OR "placenta s" OR placentae" OR transplacenta OR transplacentas OR "transplacenta s" OR transplacentae OR trans-placenta OR trans-placentas OR "trans-placenta s" OR trans-placentae OR utero OR uterus OR uteruses OR ((preterm OR pre-term OR premature OR term OR live OR still) N1 (birth OR births OR childbirth OR childbirths OR child-birth OR child-births OR infant OR infants OR "infant s" OR newborn OR newborns OR "newborn s" OR new-born OR new-borns OR "new-born s" OR neonate OR neonates OR neonatality OR neonatals OR "neonate s" OR neonatal OR neo-natal OR neo-natals OR "neo-natal s" OR neo-natality OR neo-nate OR neo-nates OR baby OR babies OR labor OR "labor s" OR labored OR laboring OR labors OR laboured OR labouring OR labours OR labour OR parturition OR parturitions OR parturitional OR parturient OR parturients OR delivery OR deliveries)) OR prematurity OR stillbirth OR stillbirths OR still-birth OR still-births OR stillborn OR stillborns OR still-born OR still-borns OR cesarean OR caesarean OR cesarian OR caesarian OR cesarien OR caesarien OR cesareans OR caesareans OR cesarians OR caesarians OR cesariens OR caesariens OR C-section OR C-sections OR abortion OR abortions OR aborted OR miscarry OR miscarries OR miscarried OR miscarriage OR miscarriages OR birthweight OR birthweights OR birth-weights OR "gestational age" OR "gestational hypertension" OR eclampsia OR eclampsias OR preeclampsia OR preeclampsias OR "pre eclampsia" OR "pre eclampsias" OR Apgar OR ((infant OR infants OR newborn OR newborns OR new-born OR new-borns OR neonate OR neonates OR baby OR babies OR mother OR mothers OR intrauterine OR intra-uterine) N1 (mortality OR mortalities OR dead OR death OR deaths OR loss OR losses OR lost OR demise OR demises OR viability))) OR AB ("embryo s" OR embryos OR embryonic OR embryo OR fetus OR fetuses OR "fetus s" OR foetu OR foetus OR foetuses OR "foetus s" OR fetal OR fetale OR fetally OR fetals OR foetal OR foetally OR foetals OR placenta OR placentas OR "placenta s" OR placentae" OR transplacenta OR transplacentas OR "transplacenta s" OR transplacentae OR trans-placenta OR trans-placentas OR "trans-placenta s" OR trans-placentae OR utero OR uterus OR uteruses OR ((preterm OR pre-term OR premature OR term OR live OR still) N1 (birth OR births OR childbirth OR childbirths OR child-birth OR child-births OR infant OR infants OR "infant s" OR newborn OR newborns OR "newborn s" OR new-born OR new-borns OR "new-born s" OR neonate OR neonates OR neonatality OR neonatals OR "neonate s" OR neonatal OR neo-natal OR neo-natals OR "neo-natal s" OR neo-natality OR neo-nate OR neo-nates OR baby OR babies OR labor OR "labor s" OR labored OR laboring OR labors OR laboured OR labouring OR labours OR labour OR parturition OR parturitions OR parturitional OR parturient OR parturients OR delivery OR deliveries)) OR prematurity OR stillbirth OR stillbirths OR still-birth OR still-births OR stillborn OR stillborns OR still-born OR still-borns OR cesarean OR caesarean OR cesarian OR caesarian OR cesarien OR caesarien OR cesareans OR caesareans OR cesarians OR caesarians OR cesariens OR caesariens OR C-section OR C-sections OR abortion OR abortions OR aborted OR miscarry OR miscarries OR miscarried OR miscarriage OR miscarriages OR birthweight OR birthweights OR birth-weights OR "gestational age" OR "gestational hypertension" OR eclampsia OR eclampsias OR preeclampsia OR preeclampsias OR "pre eclampsia" OR "pre eclampsias" OR Apgar OR ((infant OR infants OR newborn OR newborns OR new-born OR new-borns OR neonate OR neonates OR baby OR babies OR mother OR mothers OR intrauterine OR intra-uterine) N1 (mortality OR mortalities OR dead OR death OR deaths OR loss OR losses OR lost OR demise OR demises OR viability)))

3 TI (((congenital OR congenitally OR birth OR births OR development OR developments OR developmental) N1 (disorder OR disorders OR disordered OR abnormality OR abnormalities OR abnormal OR defect OR defects OR defected OR malform OR malforms OR malformed OR malforming OR malformation OR malformations OR deform OR deforms OR deforming OR deformity OR deformities OR disease OR diseases OR syndrome OR syndromes)) OR foetopath OR foetopaths OR foetopathic OR fetopath OR fetopaths OR fetopathic OR foeto-path OR foeto-paths OR foeto-pathic OR feto-path OR feto-paths OR feto-pathic OR ((mental OR growth OR psychomotor) N1 retardation) OR amniocentesis OR amniocenteses OR chorionic villi OR tocolysis OR tocolyses OR tocolytic) OR AB (((congenital OR congenitally OR birth OR births OR development OR developments OR developmental) N1 (disorder OR disorders OR disordered OR abnormality OR abnormalities OR abnormal OR defect OR defects OR defected OR malform OR malforms OR malformed OR malforming OR malformation OR malformations OR deform OR deforms OR deforming OR deformity OR deformities OR disease OR diseases OR syndrome OR syndromes)) OR foetopath OR foetopaths OR foetopathic OR fetopath OR fetopaths OR fetopathic OR foeto-path OR foeto-paths OR foeto-pathic OR feto-path OR feto-paths OR feto-pathic OR ((mental OR growth OR psychomotor) N1 retardation) OR amniocentesis OR amniocenteses OR chorionic villi OR tocolysis OR tocolyses OR tocolytic)

4 TI (breastfeed OR breastfed OR breastfeeding OR breastfeeds OR breast-feed OR breast-feeds OR breast-feeding OR breast-fed OR breastmilk OR breastmilks OR breastmilking OR breast-milk OR breast-milks OR breast-milking OR "breast pumping" OR (milk N1 (express OR expressed OR expresses OR expressing OR excrete OR excreted OR excreting OR excretion OR excretions OR release OR releases OR released OR releasing OR secrete OR secreted OR secretes OR secreting OR secretion OR secretions)) OR lactation OR lactations OR lactate OR lactated OR lactates OR lactating OR ((human OR breast OR mother OR woman OR women OR maternal) N1 (milk OR milks OR milking)) OR AB (breastfeed OR breastfed OR breastfeeding OR breastfeeds OR breast-feed OR breast-feeds OR breast-feeding OR breast-fed OR breastmilk OR breastmilks OR breastmilking OR breast-milk OR breast-milks OR breast-milking OR "breast pumping" OR (milk N1 (express OR expressed OR expresses OR expressing OR excrete OR excreted OR excreting OR excretion OR excretions OR release OR releases OR released OR releasing OR secrete OR secreted OR secretes OR secreting OR secretion OR secretions)) OR lactation OR lactations OR lactate OR lactated OR lactates OR lactating OR ((human OR breast OR mother OR woman OR women OR maternal) N1 (milk OR milks OR milking))

5 (MH "Expectant Mothers") OR (MH "Multiparas") OR (MH "Primiparas") OR (MH "Maternal-Child Care+") OR (MH "Postnatal Period+") OR (MH "Pregnancy+") OR (MH "Parity") OR (MH "Pregnancy Outcomes") OR (MH "Pregnancy, Multiple+") OR (MH "Pregnancy, Unplanned") OR (MH "Pregnancy, Unwanted") OR (MH "Maternal Nutritional Physiology+") OR (MH "Pseudopregnancy") OR (MH "Pregnancy Trimesters+") OR (MH "Pregnancy Complications+") OR (MH "Childbirth Education") OR (MH "Neonatology") OR (MH "Perinatology") OR (MH "Maternal Exposure") OR (MH "Breast Feeding+") OR (MH "Milk, Human") OR (MH "Infant, Newborn, Diseases+") OR (MH "Fetal Diseases+") OR (MH "Fetal Abnormalities") OR (MH "Prenatal Exposure Delayed Effects") OR (MH "Maternal-Child Health") OR (MH "Maternal Health Services+") OR (MH "Analgesia, Obstetrical") OR (MH "Anesthesia, Obstetrical") OR (MH "Obstetrics") OR (MH "Infant+") OR (MH "Prenatal Diagnosis+") OR (MH "Fetal Development+") OR (MH "Maternal Age+") OR (MH "Postnatal Period+")

6 S1 OR S2 OR S3 OR S4 OR S5

7 (MH "Depression, Postpartum")

8 TI (depress OR depressed OR depression OR depressions OR "depression s" OR depressive OR depressivity OR depressivities OR depressively OR depressiveness OR depressives OR dysphoria OR dysphorias OR dysphoric OR dysthymias OR dysthymic OR dysthymia OR melancholia OR melancholia OR melancholic) OR AB (depress OR depressed OR depression OR depressions OR "depression s" OR depressive OR depressivity OR depressivities OR depressively OR depressiveness OR depressives OR dysphoria OR dysphorias OR dysphoric OR dysthymias OR dysthymic OR dysthymia OR melancholia OR melancholia OR melancholic)

9 TI ((season OR seasons OR seasonal) N2 (affective OR mood OR moods OR moodiness OR moody OR depress OR depressed OR depression OR depressions OR "depression s" OR depressive OR depressivity OR depressivities OR depressively OR depressiveness OR depressives)) OR AB ((season OR seasons OR seasonal) N2 (affective OR mood OR moods OR moodiness OR moody OR depress OR depressed OR depression OR depressions OR "depression s" OR depressive OR depressivity OR depressivities OR depressively OR depressiveness OR depressives))

10 TI ((bipolar OR bi-polar OR mood OR moods OR moody OR moodiness OR affective OR manic-depression OR manic-depressive OR manic-depressives OR manic-depressed OR manic-depressivity OR manic-depressivities OR manic) N2 (disorder OR disorders OR psychosis OR psychoses OR psychotic OR symptom OR symptoms OR symptomatic)) OR AB ((bipolar OR bi-polar OR mood OR moods OR moody OR moodiness OR affective OR manic-depression OR manic-depressive OR manic-depressives OR manic-depressed OR manic-depressivity OR manic-depressivities OR manic) N2 (disorder OR disorders OR psychosis OR psychoses OR psychotic OR symptom OR symptoms OR symptomatic))

11 TI (involutional N2 (psychosis OR psychoses OR psychotic OR paraphrenia OR paraphrenias OR paraphrenic)) OR AB (involutional N2 (psychosis OR psychoses OR psychotic OR paraphrenia OR paraphrenias OR paraphrenic))

12 (MH "Affective Disorders") OR (MH "Depression+") OR (MH "Perinatal Mood and Anxiety Disorders+") OR (MH "Seasonal Affective Disorder") OR (MH "Affective Disorders, Psychotic+") OR (MH "Postpartum Psychosis")

13 S8 OR S9 OR S10 OR S11 OR S12

14 S6 AND S13

15 S7 OR S14

16 (MH "Electroshock+")

17 TI (electroconvulsive OR electroconvulsant OR electroconvulsion OR electroconvulsions OR electro-convulsive OR electro-convulsant OR electro-convulsion OR electro-convulsions OR electrovulsive OR electrovulsant OR electrovulsion OR electrovulsions OR electro-vulsive OR electro-vulsives OR electro-vulsant OR electro-vulsants OR electroshock OR electroshocks OR ECT OR ((electro OR electric OR electrode OR electrodes) N2 (convulsive OR shock OR shocks))) OR AB (electroconvulsive OR electroconvulsant OR electroconvulsion OR electroconvulsions OR electro-convulsive OR electro-convulsant OR electro-convulsion OR electro-convulsions OR electrovulsive OR electrovulsant OR electrovulsion OR electrovulsions OR electro-vulsive OR electro-vulsives OR electro-vulsant OR electro-vulsants OR electroshock OR electroshocks OR ECT OR ((electro OR electric OR electrode OR electrodes) N2 (convulsive OR shock OR shocks)))

18 S16 OR S17

19 S15 AND S18

20 ((MH "Vertebrates+") NOT MH Human)

21 S19 NOT S20

22 S21 AND LA (English)

**Database:** ClinicalTrials.gov

**Search Date:** December 31, 2024

**Limits:** N/A

**Number of Results:** 14

(electroconvulsive OR electro-convulsive OR "electro convulsive" OR electrovulsive OR electro-vulsive OR "electro vulsive" OR electroshock OR electro-shock OR "electro shock" OR electroconvulsant OR electroconvulsants OR electro-convulsant OR electro-convulsants OR "electro convulsant" OR " electro convulsants" OR electrovulsant OR electrovulsants OR electro-vulsant OR electro-vulsants OR "electro vulsant" OR "electro vulsants" OR ECT) AND (pregnant OR pregnancy OR pregnancies OR puerperium OR puerperal OR perinatal OR perinatals OR perinatally OR peri-natal OR peri-natals OR peri-natally OR prenatal OR prenatals OR prenatally OR pre-natal OR pre-natals OR pre-natally OR antenatal OR antenatals OR antenatally OR ante-natal OR ante-natals OR ante-natally OR postnatal OR postnatals OR postnatally OR post-natal OR post-natals OR post-natally OR peripartum OR peri-partum OR prepartum OR pre-partum OR antepartum OR ante-partum OR intrapartum OR intra-partum OR postpartum OR post-partum OR obstetric OR obstetrics OR obstetrically OR maternity OR maternities OR maternal OR parturient OR parturients OR perinatal OR perinatals OR perinatally OR peri-natal OR peri-natals OR peri-natally OR "expectant mother" OR "expectant mothers" OR childbirth OR childbirths OR childbirthing OR child-birth OR child-births OR child-birthing) AND (depress OR depressed OR depression OR depressions OR "depression s" OR depressive OR depressivity OR depressivities OR depressively OR depressiveness OR depressives OR dysphoria OR dysphorias OR dysphoric OR dysthymias OR dysthymic OR dysthymia OR melancholia OR melancholia OR melancholic OR affective OR mood OR bipolar OR bi-polar)

**Database:** World Health Organization (WHO) International Clinical Trials Registry Platform (ICTRP)

**Search Date:** December 31, 2024

**Limits:** N/A

**Number of Results:** 1

Intervention: (electroconvulsive OR electro-convulsive OR "electro convulsive" OR electrovulsive OR electro-vulsive OR "electro vulsive" OR electroshock OR electro-shock OR "electro shock" OR electroconvulsant OR electroconvulsants OR electro-convulsant OR electro-convulsants OR "electro convulsant" OR " electro convulsants" OR electrovulsant OR electrovulsants OR electro-vulsant OR electro-vulsants OR "electro vulsant" OR "electro vulsants" OR ECT)

Condition: (pregnant OR pregnancy OR pregnancies OR puerperium OR puerperal OR perinatal OR perinatals OR perinatally OR peri-natal OR peri-natals OR peri-natally OR prenatal OR prenatals OR prenatally OR pre-natal OR pre-natals OR pre-natally OR antenatal OR antenatals OR antenatally OR ante-natal OR ante-natals OR ante-natally OR postnatal OR postnatals OR postnatally OR post-natal OR post-natals OR post-natally OR peripartum OR peri-partum OR prepartum OR pre-partum OR antepartum OR ante-partum OR intrapartum OR intra-partum OR postpartum OR post-partum OR obstetric OR obstetrics OR obstetrically OR maternity OR maternities OR maternal OR parturient OR parturients OR perinatal OR perinatals OR perinatally OR peri-natal OR peri-natals OR peri-natally OR "expectant mother" OR "expectant mothers" OR childbirth OR childbirths OR childbirthing OR child-birth OR child-births OR child-birthing)

**Database:** Global Index Medicus

**Search Date:** December 31, 2024

**Limits:** English language

**Number of Results:** 29

((TW:electroconvuls* OR TW:electro-convuls* OR TW:electrovuls* OR TW:electro-vuls* OR TW:electroshock OR TW:electro-shock OR TW:"ECT") AND (TW:perinatal* OR TW:postnatal* OR TW:prenatal* OR TW:antenatal* OR TW:postpartum OR TW:prepartum OR TW:intrapartum OR TW:pregnan* OR TW:puerper*)) AND LA:en

**Seed Articles**

PMID: 37852926

Arnison T, Rask O, Nordenskjöld A, Movahed Rad P. Safety of AND response to electroconvulsive therapy during pregnancy: Results from population-based nationwide registries. Acta Psychiatr Scand. 2024 Nov;150(5):360-371. doi: 10.1111/acps.13623. Epub 2023 Oct 18. PMID: 37852926.

PMID: 29660641

Rundgren S, Brus O, Båve U, Landén M, Lundberg J, Nordanskog P, Nordenskjöld A. Improvement of postpartum depression AND psychosis after electroconvulsive therapy: A population-based study with a matched comparison group. J Affect Disord. 2018 Aug 1;235:258-264. doi: 10.1016/j.jad.2018.04.043. Epub 2018 Apr 9. PMID: 29660641.

PMID: 26479488

Haxton C, Kelly S, Young D, Cantwell R. The Efficacy of Electroconvulsive Therapy in a Perinatal Population: A Comparative Pilot Study. J ECT. 2016 Jun;32(2):113-5. doi: 10.1097/YCT.0000000000000278. PMID: 26479488.

PMID: 20562638

O'Reardon JP, Cristancho MA, von Andreae CV, Cristancho P, Weiss D. Acute AND maintenance electroconvulsive therapy for treatment of severe major depression during the second AND third trimesters of pregnancy with infant follow-up to 18 months: case report AND review of the literature. J ECT. 2011 Mar;27(1):e23-6. doi: 10.1097/YCT.0b013e3181e63160. PMID: 20562638.

PMID: 20375702

Pesiridou A, Baquero G, Cristancho P, Wakil L, Altinay M, Kim D, O'Reardon JP. A case of delayed onset of threatened premature labor in association with electroconvulsive therapy in the third trimester of pregnancy. J ECT. 2010 Sep;26(3):228-30. doi: 10.1097/YCT.0b013e3181c3aef3. PMID: 20375702.

PMID: 17804997

Bozkurt A, Karlidere T, Isintas M, Ozmenler NK, Ozsahin A, Yanarates O. Acute AND maintenance electroconvulsive therapy for treatment of psychotic depression in a pregnant patient. J ECT. 2007 Sep;23(3):185-7. doi: 10.1097/YCT.0b013e31806db4dd. PMID: 17804997.

PMID: 8198651

Livingston JC, Johnstone WM Jr, Hadi HA. Electroconvulsive therapy in a twin pregnancy: a case report. Am J Perinatol. 1994 Mar;11(2):116-8. doi: 10.1055/s-2007-994569. PMID: 8198651.
